# Dynamic genetic regulation of CD4^+^ T cells in obstructive sleep apnea: integrating context-specific eQTL, Mendelian randomization, single-cell sequencing, and experimental validation

**DOI:** 10.3389/fimmu.2025.1691347

**Published:** 2025-12-17

**Authors:** Xinyue Zhang, Lingfei Ren

**Affiliations:** 1 Department of Respiratory and Critical Care Medicine, Sichuan Provincial People’s Hospital, University of Electronic Science and Technology of China, Chengdu, China; 2 School of Computing and Artificial Intelligence, Southwestern University of Finance and Economics, Chengdu, China

**Keywords:** CD4^+^ T cells, context-specific eQTL, genetic colocalization, Mendelian randomization, obstructive sleep apnea, single-cell sequencing

## Abstract

**Objective:**

To investigate the dynamic genetic regulatory mechanisms of CD4^+^ T cells in the pathogenesis of obstructive sleep apnea (OSA), particularly in the immune and inflammatory response induced by intermittent hypoxia (IH).

**Methods:**

This study integrated context-specific expression quantitative trait locus analysis, Mendelian randomization, colocalization analysis, single-cell RNA sequencing, and qPCR experimental validation. A systematic investigation was conducted on gene expression and genetic variation in CD4^+^ T cells obtained from 119 donors of European descent across multiple activation time points including zero hours, sixteen hours, forty hours, and five days. Functional validation was performed using an IH mouse model.

**Results:**

The study identified multiple genes demonstrating a causal relationship with OSA risk, such as *MAST3*, *FNBP4*, *SPNS1*, and *AKIRIN1*. Thirteen expression quantitative trait loci showed significant colocalization with OSA genome-wide association study signals, with a posterior probability of shared causal variants exceeding zero point eight five. Experimental validation in the IH mouse model demonstrated significantly upregulated mRNA expression levels of *Fnbp4* and *Mast3*, alongside downregulated expression of *Sgf29*, *Sh3yl1*, and *Tufm* within CD4^+^ T cells.

**Conclusion:**

The immune regulation mediated by CD4^+^ T cells demonstrates significant temporal dynamics and cell type specificity in OSA pathogenesis. Key genes including *TUFM*, *MAST3*, *FNBP4*, *SGF29*, and *SH3YL1* participate in the pathological process by regulating mitochondrial function, cell migration, transcriptional regulation, and inflammatory responses. These findings provide a novel theoretical foundation and reveal potential targets for personalized therapeutic strategies and biomarker development in OSA.

## 1 Introduction

The primary characteristic of obstructive sleep apnea (OSA) is the recurrent collapse of the upper airway and disruption of airflow during sleep, leading to intermittent hypoxia (IH) and sleep fragmentation. Approximately 1 billion individuals globally are impacted by OSA, with 425 million experiencing moderate to severe manifestations of the condition. The incidence of OSA is increasing due to demographic aging and the obesity epidemic (1). Despite the advancements in home-based portable monitoring and continuous positive airway pressure therapy that have enhanced illness screening and treatment accessibility, the misdiagnosis rate can reach 80%, and low treatment compliance continues to pose substantial public health concerns globally.

OSA is not only a respiratory disorder but also a catalyst for multisystem impairment. It has been identified as an independent risk factor for cardiovascular diseases, including hypertension, coronary heart disease, and heart failure (2), and is significantly associated with metabolic and neurological diseases as well as end-organ damage exemplified by conditions such as erectile dysfunction (3). The pathophysiological processes of OSA are significantly more intricate than mere mechanical obstruction of the upper airway. Systemic chronic inflammation caused by IH is regarded as a primary mechanism facilitating multi-organ damage in OSA. Frequent variations in oxygen saturation during sleep can stimulate hypoxia-inducible factors, initiate oxidative stress responses, and perpetuate inflammatory pathways, resulting in markedly elevated levels of several pro-inflammatory factors, including CRP, IL-6, and TNF-α. The coupling mechanism of hypoxia and inflammation not only worsens functional deficits in upper airway structures (4), but also significantly contributes to the development of cardiovascular problems associated with OSA.

In inflammatory reactions, CD4^+^ T cells act as primary regulators of the adaptive immune system, and their functional state experiences substantial alterations in an IH environment. Prior research has demonstrated that IH can prompt CD4^+^ T cells to adopt a pro-inflammatory phenotype and intensify their interactions with other immune cells, including monocytes and B cells, thus aggravating systemic inflammatory responses. The activation state of CD4^+^ T lymphocytes is acutely responsive to temporal fluctuations and microenvironmental factors, with their gene expression regulation patterns demonstrating considerable dynamic plasticity under varying stimulatory settings. The genetic mechanisms governing the regulation of inflammatory phenotype transitions in CD4^+^ T cells, especially regarding how genetic regulation contributes to immunological dysfunction in the pathophysiological context of OSA, remain inadequately elucidated.

In recent years, genome-wide association studies (GWAS) have progressively identified several genetic susceptibility loci linked to OSA (5). Nonetheless, these investigations have predominantly concentrated on statistical correlations between static genetic variants and illness risk, complicating the elucidation of the dynamic regulatory mechanisms of gene expression. In inflammation-related disorders, the dynamic alterations in cellular functional states profoundly affect gene expression, and conventional GWAS fail to capture the intricacies of regulatory processes. Moreover, existing immunogenetic research on OSA remains constrained, and the genetic foundation underlying the imbalance of CD4^+^ T cell subsets and the modulation of pro-inflammatory variables necessitates comprehensive investigation.

Expression Quantitative Trait Loci (eQTL) analysis serves as a crucial instrument for the functional annotation of genetic variation in non-coding areas, establishing a causal relationship between genetic variation and gene expression levels (6). Traditional eQTL investigations predominantly utilize cells or tissues in resting states, thereby complicating the elucidation of dynamic alterations in cellular function throughout illness progression. Context-specific eQTL approaches may examine the temporal patterns of gene regulation under specific stimuli, particularly effective in elucidating the temporal responses of immune cells in inflammatory microenvironments (7), thereby offering a novel perspective for modeling the mechanisms of OSA.

Mendelian randomization (MR) employs genetic variation as an instrumental variable (IV) to circumvent confounding biases and reverse causality in conventional observational studies, thereby inferring causal relationships between exposure and result (8). Integrating context-specific eQTL with MR identifies pivotal genes with causal impacts on disease risk during specific activation phases and captures the temporal variations in their regulatory effects, thus providing a theoretical foundation for targeted interventions on disease-associated genes (9).

This study generated single-cell transcriptomic and genotypic data for CD4^+^ T cells at four distinct activation time points (0 h, 16 h, 40 h, and 5 d) and employed context-specific eQTL analysis, MR, multi-omics integration, and experimental validation techniques (qPCR) to comprehensively investigate the dynamic genetic regulatory network of CD4^+^ T cells in the pathogenesis of OSA. Our objective is to elucidate how CD4^+^ T cell-specific eQTLs facilitate immunological dysregulation generated by IH, understand their mechanisms in the multi-organ damage associated with OSA, and identify therapeutic targets with potential for intervention.

## 2 Materials and methods

### 2.1 Context-specific eQTL identification in CD4^+^ T cells

**Figure 1:**
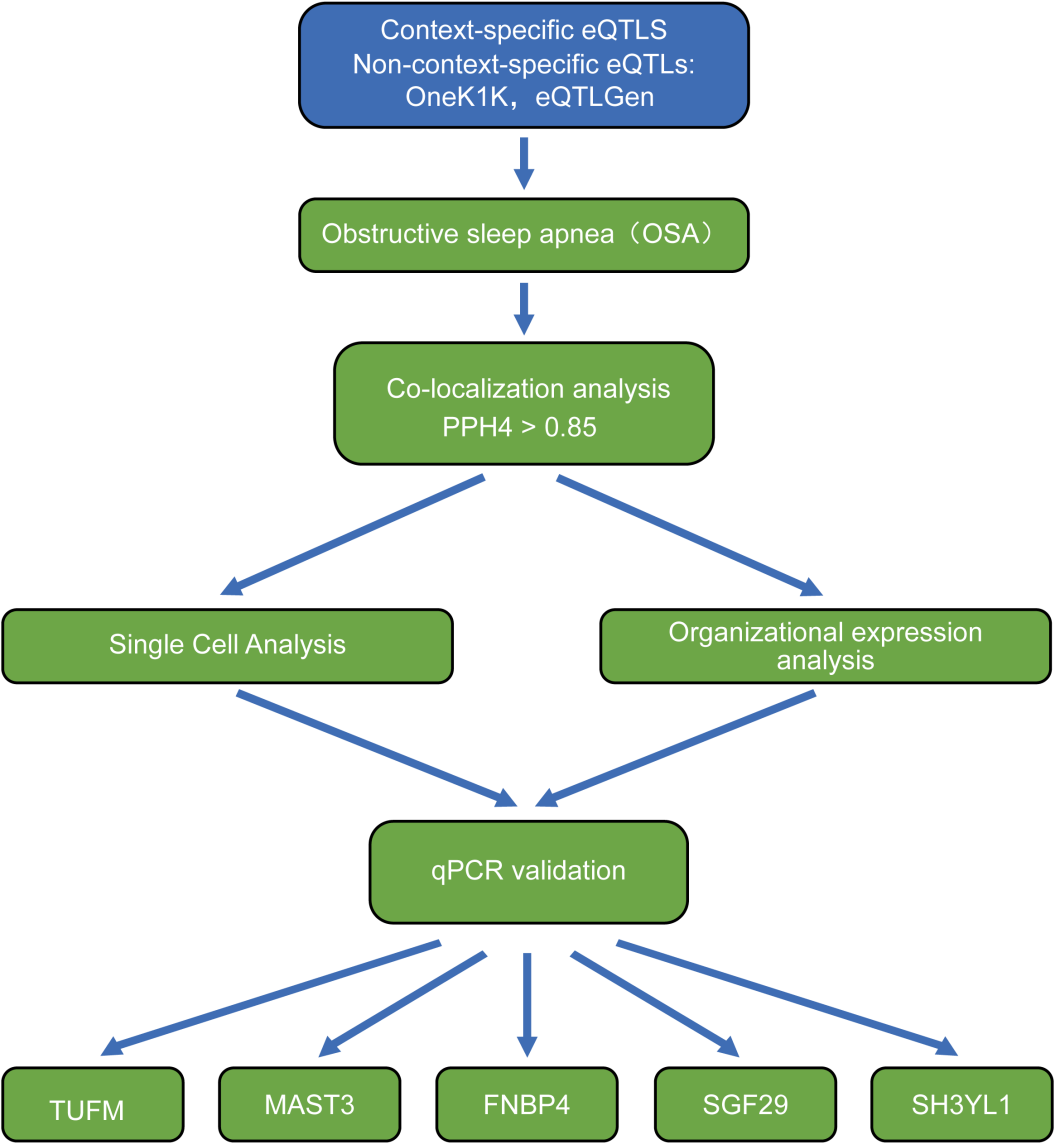
The workflow for causal inference of context-specific CD4^+^ T cell eQTLs and OSA.

We employed scRNA-seq GWAS data from 119 donors of European descent to identify cis-eQTLs in CD4^+^ T cells in both resting and active states. These time points were selected to capture key phases of the T cell response, including the resting state (0h), early activation (16h), intermediate metabolic shifts (40h), and late effector differentiation (5d) (10). A total of 482,971 cis-eQTLs were discovered within an action window of ±1 Mb. Subsequently, MR experiments were performed with OSA, and their expression was identified in IH mouse single-cell sequencing, as illustrated in **Figure 1**.

The IV screening procedure has three stages: 1) To identify cis-eQTLs independent of selection conditions, we utilized PLINK 1.9 software to conduct linkage disequilibrium (LD) clustering based on the 1000 Genomes European population reference panel (LD threshold r² < 0.001, window size = 10 Mb); 2) We calculated the F statistic for each SNP (F = β²/SE²) and retained only robust IVs with F values exceeding 10; 3) We employed the Steiger directionality test to eliminate SNPs that might indicate reverse causality. Context-specific eQTLs were defined as genotype-expression associations that were significantly detected (FDR < 0.05) in specific activation states (e.g., 16h, 40h, or 5d) but showed differential patterns compared to the resting state.

### 2.2 Acquisition and processing of static eQTL data

To maintain consistency, we applied the identical IVs screening methodology, comprising LD clustering, F > 10 filter, and the Steiger test) on static blood eQTL resources. We acquired the following data: resting state cis-eQTLs from the OneK1K database (covering 14 immune cell subtypes); whole blood cis-eQTLs from the eQTLGen database (a large-scale study comprising 31,684 individuals across 37 cohorts); and immune cell-specific eQTLs from the DICE database.

### 2.3 OSA GWAS results data

The summary statistics for OSA were obtained from the GWAS Catalog database (https://www.ebi.ac.uk/gwas/). The sample, comprised predominantly of individuals of European ancestry, included 126,695 cases of OSA and 303,630 control subjects.

### 2.4 MR analysis

We employed the Wald ratio method to evaluate the causal effect for exposure factors characterized by a single SNP. When multiple SNPs functioned as IVs for a gene, we applied inverse variance-weighted (IVW) method. The strength of all IVs was assessed using the F-statistic (F = β²/SE²), with only strong instruments (F > 10) retained; the complete list of IVs and their F-statistics is provided in **Supplementary Material 1**. To further ensure robustness, all IVs were cross-referenced against the GWAS Catalog to exclude SNPs associated with known confounders. For exposures instrumented by three or more SNPs, we performed a suite of sensitivity analyses to detect potential horizontal pleiotropy, including the MR-Egger intercept test, Cochran’s Q test for heterogeneity, and the MR-PRESSO test for outlier detection, with full results available in **Supplementary Material 2**. P-values were adjusted for multiple comparisons via the Benjamini-Hochberg FDR method, with an FDR threshold of < 0.05 deemed statistically significant.

### 2.5 Co-localization analysis

To determine if the observed relationships were influenced by a common causal variation, we conducted Bayesian colocalization analysis between eQTL and GWAS signals utilizing the coloc package in R. We delineated colocalization regions as ± 500 kb windows surrounding significant eQTL dominant variant loci. This method tests five mutually exclusive hypotheses: H0 (no association with either trait), H1 (association with the eQTL only), H2 (association with the GWAS trait only), H3 (both traits are associated but with distinct causal variants), and H4 (both traits are associated and share a single common causal variant). Using the default prior probabilities (p1 = 1×10⁻^4^, p2 = 1×10⁻^4^, and p12 = 1×10⁻^5^), we calculated posterior probabilities for each hypothesis. A posterior probability for H4 (PPH4) > 0.85 was considered strong evidence for a shared causal variant.

### 2.6 Immune cell-specific expression profile analysis

We utilized data from the DICE Database Consortium, encompassing five principal immune cell types: CD4^+^ T cells, CD8^+^ T cells, B cells, natural killer (NK) cells, and monocytes, to assess the expression levels of potential genes across various cell types.

### 2.7 Single-cell RNA sequencing analysis of IH mouse models

Raw scRNA-seq data acquired from the GEO database (accession number: GSE145435) were processed with the Seurat software package as follows: 1) Cell filtering: Cells exhibiting fewer than 4,000 detected genes per cell (nFeature_RNA) or fewer than 15,000 detected molecules per cell (nCount_RNA) were omitted. 2) Data normalization and identification of highly variable genes were conducted. 3) Dimensionality reduction and clustering: Principal component analysis (PCA) was conducted. Cells were clustered utilizing the Louvain clustering technique, with the resolution parameter established at 0.3, based on the PCA results. 4) Visualization and cell type annotation: Employ the UMAP approach to visualize the clustering outcomes (11). Utilize the SingleR software package, in conjunction with the ImmGen reference dataset, to annotate the clustering clusters with cell types. 5) Cell communication and developmental trajectory inference: Utilize CellChat v1.1.3 software to deduce cell-cell communication networks. Utilize Monocle 2 software to reconstruct the pseudo-temporal developmental pathways of cells.

### 2.8 Establishment of IH mouse model

Six-week-old pathogen-free male C57BL/6 mice were acclimated to a 12-hour light/12-hour dark cycle for one week. The mice were thereafter divided into two groups of five each at random: 1) Control group: subjected to ambient oxygen; 2) IH group: mice in this cohort were in a specially engineered chamber where the flow rates of nitrogen and oxygen were regulated by a timed electromagnetic valve system. In each 80-second cycle of intermittent hypoxic exposure, nitrogen was administered for the initial 40 seconds at a flow rate that decreased the inspired oxygen concentration to below 10% within the first 10 seconds, succeeded by a hypoxic period where FiO_2_ was sustained at an average of 6–8%; subsequently, air was provided for the following 40 seconds at a flow rate that reinstated FiO_2_ to 21%. Mice were confined in this chamber for 8 hours daily over a duration of 6 weeks. In the control group exposure, the incoming gas consistently consisted of room air, with noise and airflow disturbance levels matching those of the IH exposure group. All animal research received approval from the Ethics Committee for Basic and Clinical Research of Sichuan Academy of Medical Sciences and Sichuan Provincial People’s Hospital (No: 2025-672).

### 2.9 Isolation of CD4^+^ T cells from the spleen

Spleens were extracted from C57BL/6 mice under aseptic conditions, mechanically homogenized, and erythrocytes were eliminated using ACK lysis buffer. Magnetic bead sorting was conducted utilizing a mouse CD4^+^ T cell negative selection kit. The single-cell culture was incubated at 4°C for 15 minutes with a combination of biotinylated antibodies and anti-biotin magnetic beads and thereafter separated using an LS sorting column. CD4^+^ T cells were isolated from the flow-through liquid using negative selection. The final cells were resuspended in PBS buffer with 0.5% BSA and 2 mM EDTA, and purity was confirmed by flow cytometry, with CD3^+^CD4^+^ double-positive cells exceeding 95%.

### 2.10 Real-time fluorescent quantitative PCR

Total RNA was isolated from CD4^+^ T cells with the UNIQ-10 Column-Based Trizol Total RNA Extraction Kit (Shengong Biotechnology Co., Ltd., China, catalog number B511321). The quality of the extracted RNA was evaluated using a microvolume spectrophotometer (Thermo Scientific, USA, model NanoDrop 1000). cDNA was produced with the cDNA Synthesis Kit (Shengong Biotechnology Co., Ltd., China, catalog number B532435). qPCR was conducted utilizing the 2 × SYBR Green Fast qPCR Mix (Biomarker, China, catalog number RK02001) on a real-time fluorescent qPCR apparatus (Bio-Rad, USA, model CFX Conne). The qPCR reaction parameters were 3 minutes of pre-denaturation at 95°C, succeeded by 40 cycles of 5 seconds of denaturation at 95°C and 30 seconds of annealing/extension at 60°C. The expression levels of each target gene were determined using the 2^-ΔΔCt^ method. The primer sequences utilized for qPCR are enumerated in **Table 1**.

**Table 1:** Primer sequences for qPCR.

Gene name	Forward	Reverse
Sh3yl1	ggaaacctgacccttggagg	cagacagcttccttccaggg
Pabpc4	gaccaaagctgtcaccgaga	ttaagatggcactggcaggg
Mast3	catggcacgcatgtacttcg	catgctcatgaggccgatct
Fnbp4	gaggaggaagaggaggagca	ggctgatgtcagaacgtgga
Fubp1	cccctggctttcatcatggt	cagcaccagtgttttgaggc
Pbrm1	tcatgccctatacaccccca	tgctgagctccccaaaagag
Nt5dc2	agatgagcggcttctatggc	cctttacatgcacatcgcgg
Ndufs5	ctataagaacgccgctcggt	cttgcactcctttttcgccc
Spns1	tgaaagacgtggctggagac	gtgccagtgccttcagatct
Akirin1	gtcttccaactcccgagcaa	acaggcttcgctttgactga
Sgf29	atatccccagaccacctgct	ctccttacaagccaccacgt
Plec	gactcaagctgacggtggaa	atggcctggaagagggagat
Tufm	gccccatgtgaatgtgggta	tgggctgcattgatggtgat
Hprt1	cagtcccagcgtcgtgatta	ggcctcccatctccttcatg

### 2.11 Prediction of protein-compound binding

Data were obtained from the Comparative Toxicogenomics Database (CTD). Compound interaction data for the target genes TUFM, MAST3, and FNBP4 (downregulated group) and SH3YL1 and SGF29 (upregulated group) were evaluated based on the following criteria: 1) Interaction behavior should be distinctly categorized as “decreases^expression” (downregulated group) or “increases^expression” (upregulated group); 2) Redundant entries for the same compound (e.g., bisphenol A at the mRNA level/protein level) have been consolidated. Distinct modes of action, such as methylation and protein-level inhibition, have been individually annotated and elucidated.

### 2.12 Statistical analysis

The utilized software packages comprised TwoSampleMR, COLOC, Seurat, Monocle, and CellChat. All data are presented as mean ± standard deviation (mean ± SD). Differences between the two groups were evaluated using a student’s t-test in GraphPad Prism 9.5.1 software. *P* < 0.05 was deemed statistically significant.

## 3 Results

### 3.1 IVs selection

We utilized context-specific eQTL data to develop IVs for MR trials. Through a single-cell transcriptomics study of CD4^+^ T cells across 46 cell type/time point combinations, we preliminarily identified 482,971 cis-eQTLs associated with 13,379 genes. This procedure ultimately filtered 9,644 independent SNPs encompassing 1,538 genes (**Supplementary Material 3**).

To evaluate the additional value of context-specific eQTLs, we employed identical screening criteria on two static blood eQTL resources-OneK1K (sample size n = 982) and eQTLGen (sample size n = 1,418-31,684)-yielding 9,723 and 65,904 IVs, respectively (**Supplementary Materials 4**, **5**). A comparative investigation revealed several context-specific regulatory regions that are only active during T cell activation conditions. This comparison of IV counts is for descriptive purposes to illustrate the scale of each resource and does not influence the subsequent MR causal inference.

### 3.2 Causal effects of context-specific CD4^+^ T cell eQTLs on OSA

**Figure 2:**
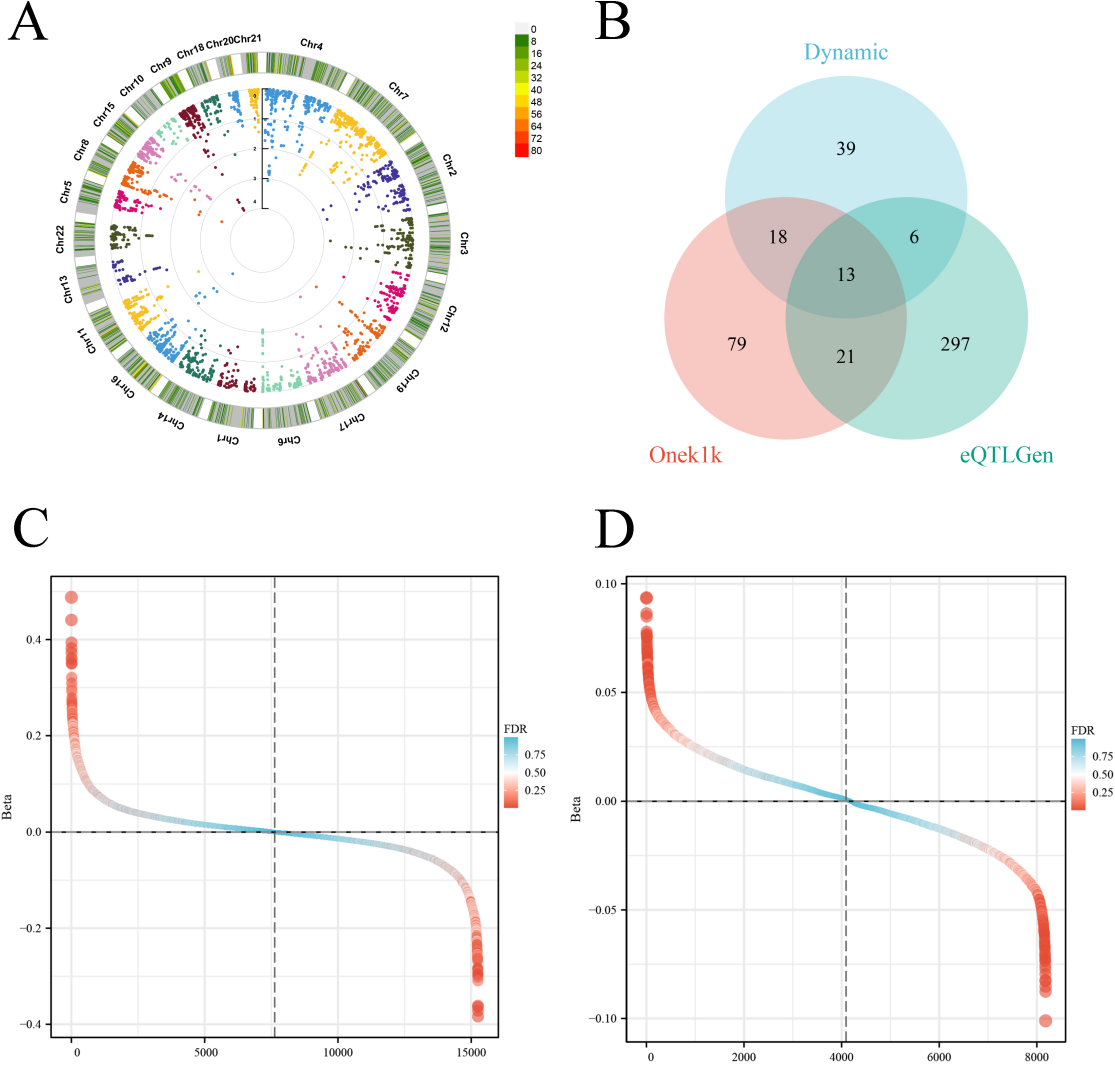
The results of causal inference of context-specific CD4^+^ T cell eQTL and OSA. **(A)**. The circular Manhattan plot displays the genome-wide distribution of significant eQTLs from context-specific CD4^+^ T cells associated with OSA and T cell status. **(B)**. The Venn diagram illustrates the intersection of genes showing significant MR-inferred causal associations with OSA, identified using context-specific eQTL and two static eQTL datasets (OneK1K and eQTLGen). The differential ranking diagram illustrates the causal relationship between plasma eQTL **(C)** and OneK1K **(D)** single-cell eQTL and OSA.

We employed MR methods to evaluate the causal impacts of context-specific CD4^+^ T cell eQTLs on the risk of OSA. The research identified multiple genes with consistently directional effects in response to cellular activation states: MAST3, FNBP4, SPNS1, and AKIRIN1 genes were associated with an increased risk of OSA, whereas NT5DC2, SH3YL1, PABPC4, FUBP1, PBRM1, NDUFS5, SGF29, and PLEC genes demonstrated a protective effect against OSA (**Figure 2A**, **Supplementary Figures S1**-**S6**, and **Supplementary Material 6**). The Steiger directionality test verified that none of the detected associations exhibited a reverse causal link (**Supplementary Material 7**. In total, after applying FDR correction (q < 0.05), 437 genes demonstrated a significant causal association with OSA risk.

### 3.3 MR analysis of OSA for non-context-specific eQTLs

The MR method was employed to evaluate the causal impact of static CD4+ T cell eQTLs on the risk of OSA. Analysis of the OneK1K and eQTLGen datasets identified 290 and 337 significant causal genes after FDR correction, respectively, with 13 genes being shared across all three datasets (**Figure 2B**). Notably, C2orf47 (OR = 0.681; 95% CI: 0.593-0.782; FDR = 7.84 × 10^-5^) and ERBB2IP (OR = 0.689; 95% CI: 0.577-0.823; FDR = 0.005) exhibited significant protective effects, whereas C15orf27 (OR = 1.553; 95% CI: 1.302-1.853; FDR = 0.0006) and CADM2 (OR = 1.628; 95% CI: 1.394-1.902; FDR = 3.54 × 10^-6^) demonstrated a positive association, indicating an increased risk (**Figure 2C**, **Supplementary Figures S7**-**S11**, **Supplementary Material 8**). The Steiger test eliminated the possibility of reverse causation (**Supplementary Material 9**).

MR analysis focused on specific cell types indicated that the L3MBTL3 gene confers a protective effect in monocytes, memory B cells (B-memory), naive B cells (B-naïve), and naive CD8⁻ T cells (CD8⁻ naïve T cells); conversely, in CD8⁻ effector T cells (CD8⁻ effector), CD4^+^ effector T cells (CD4^+^ effector), and natural killer cells (NK cells), this gene elevates the risk of OSA (**Figure 2D**, **Supplementary Figures S12**-**S15**, **Supplementary Materials 10**, **11**). This discovery indicates the regulation of OSA risk is exclusive to certain cell types via the L3MBTL3 gene.

### 3.4 Co-localization analysis of CD4^+^ T cell eQTLs and OSA GWAS signals

To enhance the validation of causal linkages and identify common genetic variations, we conducted a colocalization analysis of context-specific CD4^+^ T cell eQTLs with OSA GWAS results. This analysis identified 16 eQTLs across various immunological subtypes and stimulation circumstances, exhibiting a markedly high posterior probability of shared causative variants with OSA signals (PPH4 > 0.85): AKIRIN1 (in naïve CD4 cells at 16 hours, PPH4 = 0.911; in central memory T cells (TCM) at 16 hours, PPH4 = 0.988), FNBP4 (in unstimulated CD4 memory cells, PPH4 = 0.855), FUBP1 (in central memory T cells (TCM) at 16 hours, PPH4 = 0.861), MAST3, NDUFS5 (in natural regulatory T cells (nTreg) at 40 hours, PPH4 = 0.919), NT5DC2, PABPC4, PBRM1, PLEC, SGF29, SH3YL1, SPNS1, and TUFM (see **Supplementary Material 12** for comprehensive analysis). Regional association plots for the representative loci MAST3 and FNBP4 provide visual support for this shared causality (**Supplementary Material 13**). Non-coding RNAs (AC004148.1, NCK1-DT, and ASB16-AS1) were omitted.

### 3.5 Temporal causal structure of resting state and activated state

**Figure 3:**
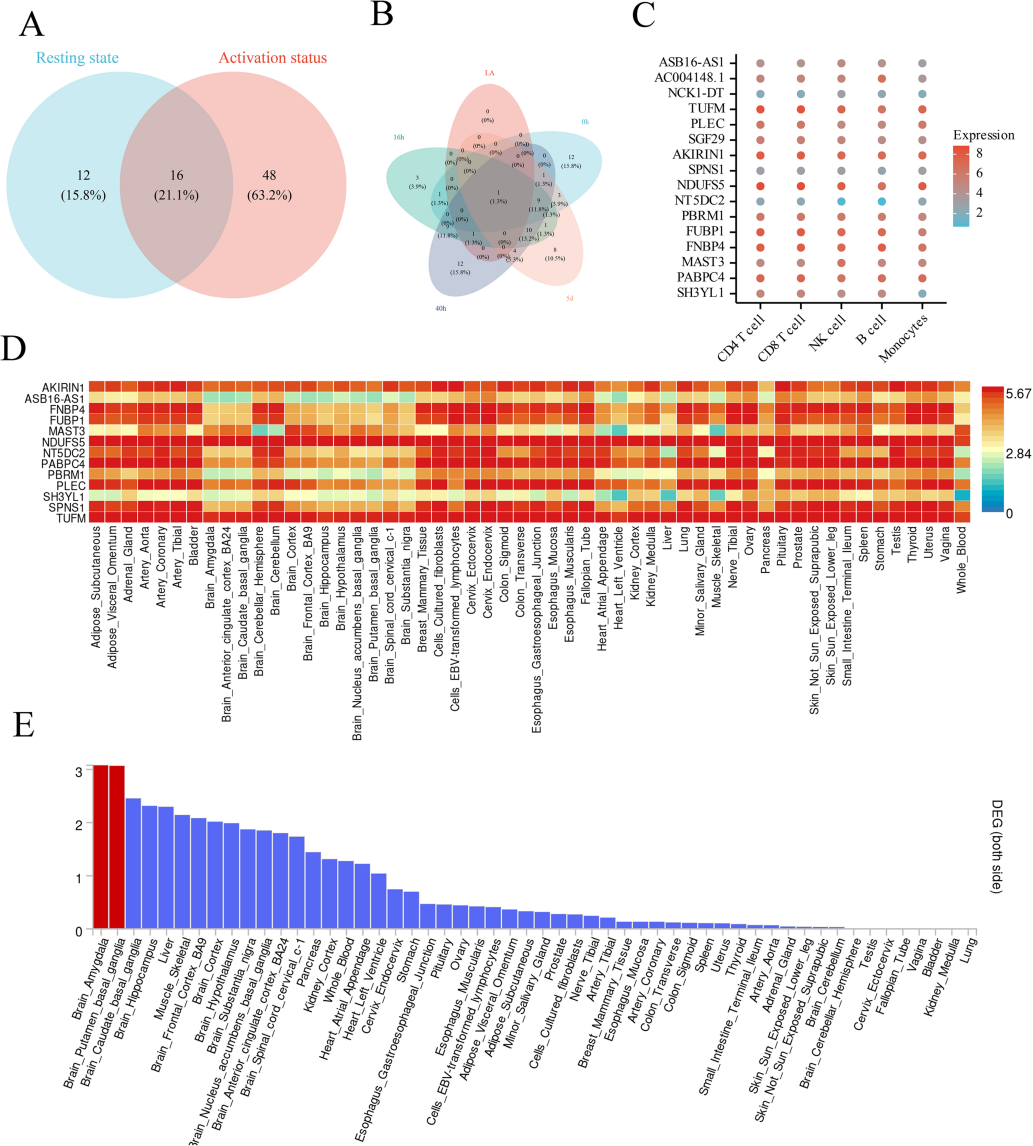
The intersection of causal relationships between context-specific and static eQTLs and the differential expression of co located genes in cells and tissues for OSA. **(A)**. The Venn diagram illustrates the intersection relationship between context-specific eQTL and static eQTL. **(B)**. The Venn diagram illustrates the intersection relationship between different time periods in context-specific eQTL. **(C)**. The dot plot shows the expression of genes with strong co localization evidence in single cells in the DICE database. **(D)**. The heatmap displays the expression relationships of genes with strong co localization evidence in different tissues. The color scale represents log-transformed gene expression levels. **(E)**. The heatmap displays the differential expression of genes with strong co localization evidence in different tissues, with red representing P<0.05.

By merging MR and colocalization analysis, we assessed the causal eQTL signal patterns of CD4^+^ T cells in various activation states. Among the colocalized genes, 12 (15.8%) were exclusively active in the resting state, 48 (63.2%) were exclusively active in the activated state, and 16 (21.1%) were active in both states (**Figure 3A**). Time-stratified analysis elucidated distinct causative gene sets for each activation stage (0h, 16h, 40h, 5d), with a solitary gene common to all time points (**Figure 3B**), underscoring the pronounced temporal specificity of gene regulation. Analysis of expression profiling for potential genes in the DICE database revealed that most genes displayed peak expression levels in CD4^+^ and CD8^+^ T cells (**Figure 3C**). Heatmaps from the GTEx database corroborated elevated expression levels in central nervous system regions (e.g., anterior cingulate cortex, hippocampus) and immunological tissues (spleen, blood) (**Figure 3D**). Tissue enrichment analysis demonstrated substantial enrichment of these genes in the amygdala and nucleus accumbens (**Figure 3E**), indicating a synergistic regulation mechanism between the neurological and immune systems.

### 3.6 Single-cell expression analysis of co-localized genes in the IH model

**Figure 4:**
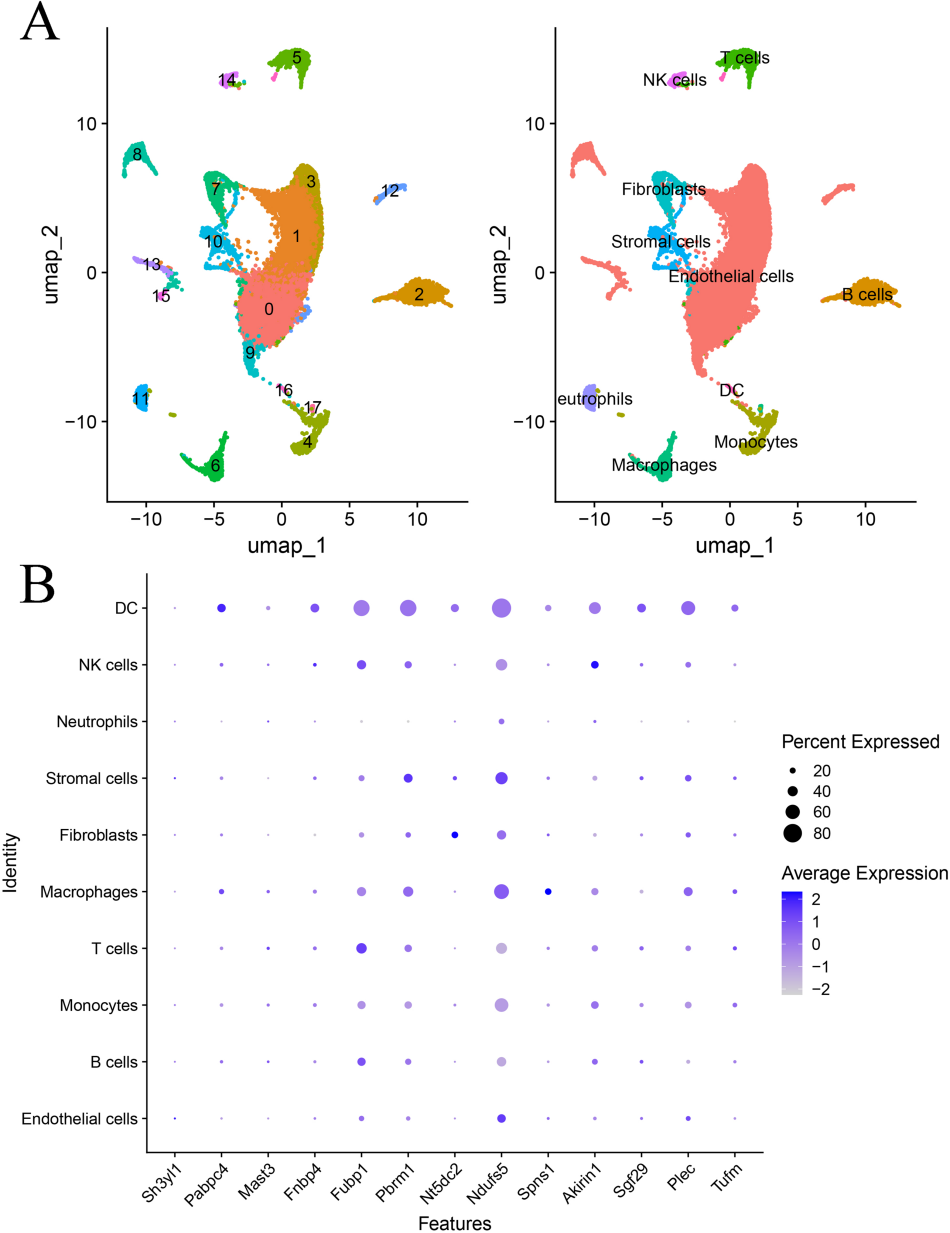
Annotation of single-cell data and expression of genes with strong co-localization evidence in single cells. **(A)**. Annotation of single-cell data. **(B)**. Single-cell gene expression with strong colocalization evidence.

**Figure 5:**
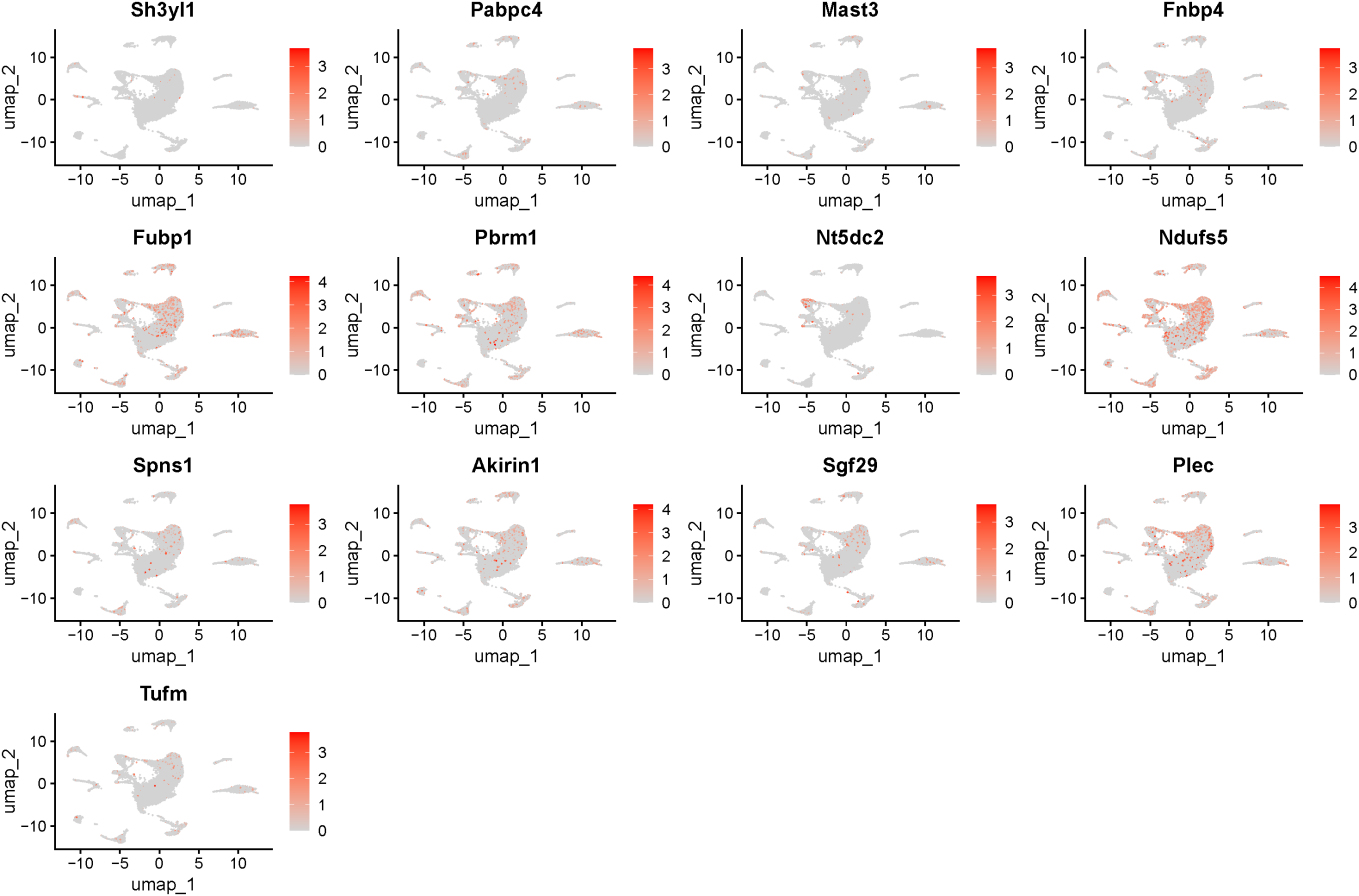
Expression location of genes with strong co localization evidence in single-cell data.

Utilizing single-cell RNA sequencing (scRNA-seq) data from the IH mouse model (GEO database number: GSE145435), we conducted unsupervised clustering (UMAP dimensionality reduction visualization) and accurately identified 10 principal cell types (see **Figure 4A**). Co-localized genes exhibited distinct expression patterns in each cell type and demonstrated elevated detection rates (**Figures 4B** and **5**).

### 3.7 Pseudo-time series analysis and T cell communication networks

**Figure 6:**
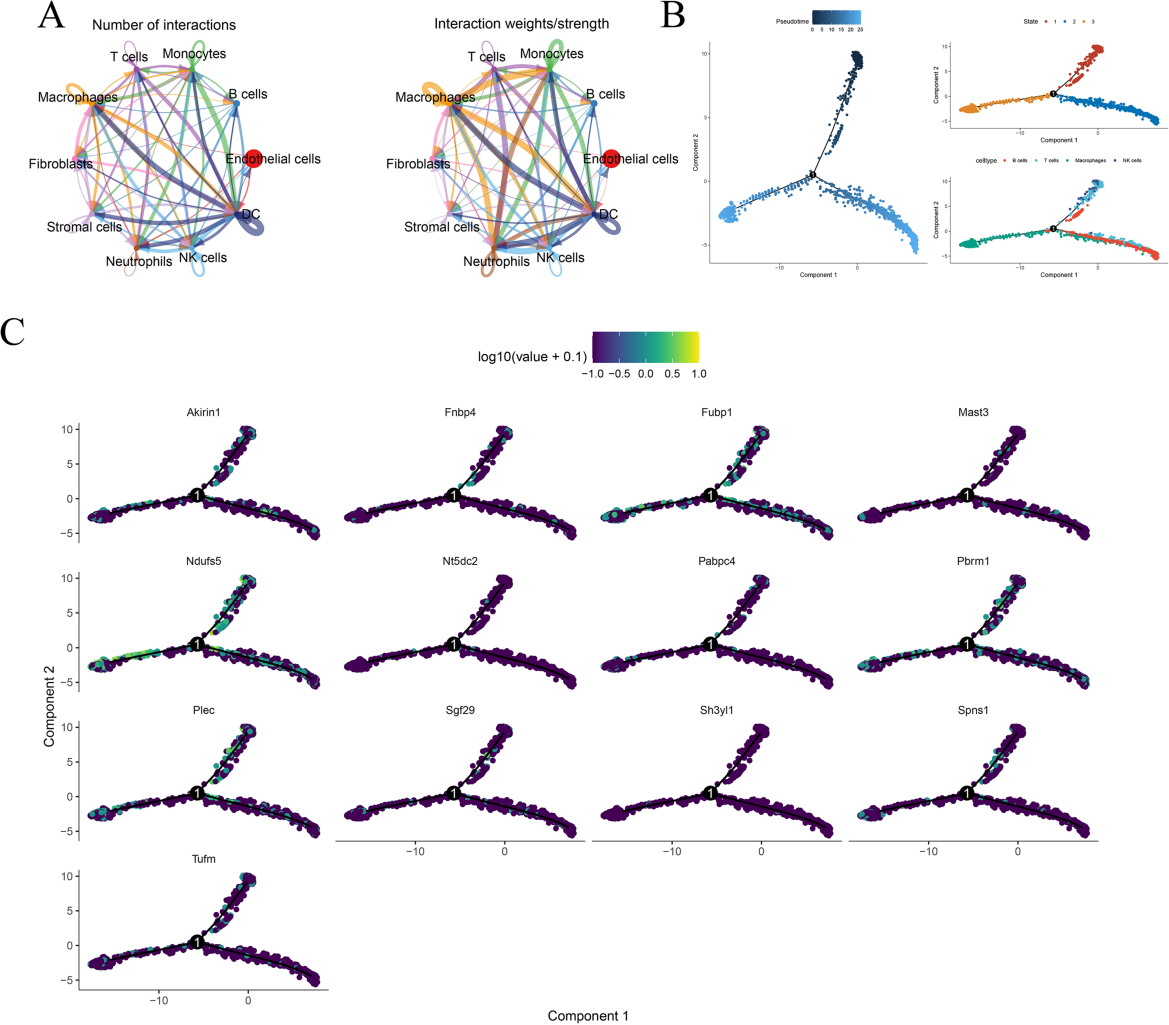
Cell communication network and pseudo temporal analysis. **(A)**. The intercellular ligand receptor interaction network is represented by the number of interactions on the left and the strength of interactions on the right. **(B)**. Pseudo temporal trajectory reconstructed by Monocle2: colored by pseudo time in the upper left corner, labeled with branch status in the upper right corner, and displayed with the distribution of major cell types in the trajectory in the lower right corner. **(C)**. The expression trajectories of 13 key genes (such as Akirin1, Fubp1, etc.) on pseudo temporal (Component 1 vs. Component 2) were mapped using color mapping log_10_ (p-value+0.1), with arrows indicating the starting point.

The CellChat study of 10 cell types demonstrated extensive ligand-receptor interactions, particularly within the communication networks among macrophages and T cells, dendritic cells and endothelial cells, as well as fibroblasts and stromal cells (**Figure 6A**). Monocle 3 pseudo-time analysis delineated immune cell developmental pathways into three branches: T cells/NK cells, B cells, and macrophages (**Figure 6B**). Analysis of the expression dynamics of 12 pivotal transcription factors along the pseudo-time trajectory demonstrated that Akirin1/Fnip1 was upregulated during the initial phases of inflammation onset, whereas Sh3yl1/Tufm attained its expression peak in the later stages of tissue remodeling (**Figure 6C**), illustrating the temporal characteristics of immune activation.

### 3.8 IH causes differences in the expression of co-localized genes and predicts their binding with compounds

**Figure 7:**
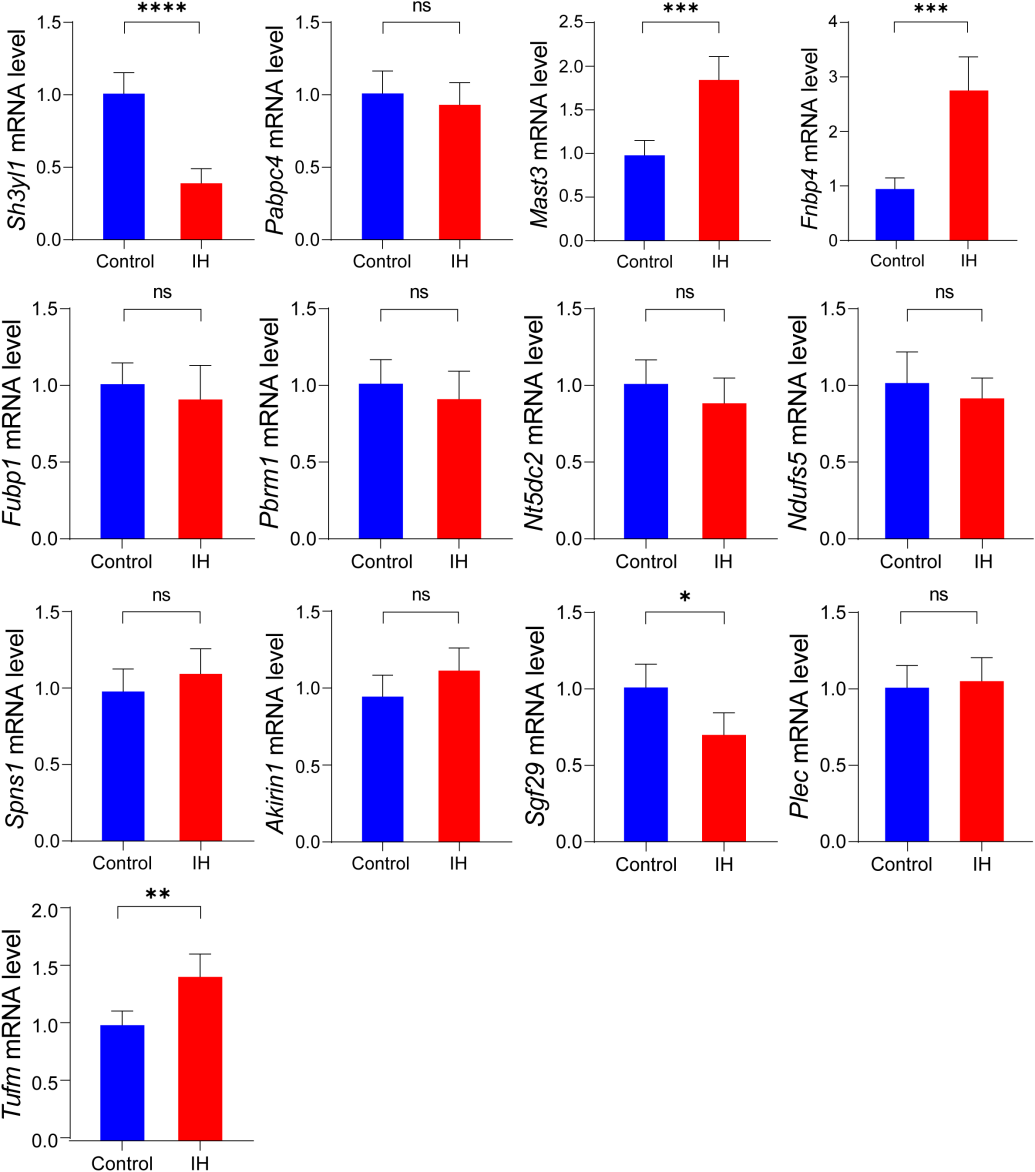
qPCR experimental verification of 13 significantly co-localized genes. Data are presented as mean ± SD (n = 5 mice per group). Statistical significance was determined by Student’s t-test. ^*^p < 0.05, ^**^p < 0.01, ^***^p < 0.001, ^****^p < 0.0001.

We confirmed the expression alterations of the highest priority genes identified in the colocalization study using the IH mouse model. **Figure 7** illustrates that, in comparison to the control group, the mRNA expression levels of FNBP4, MAST3, and TUFM were elevated in CD4^+^ T cells from IH mice, while those of SGF29 and SH3YL1 were diminished. The findings validate that FNBP4, MAST3, SGF29, SH3YL1, and TUFM constitute the fundamental molecular network governing immune modulation in OSA. Analysis of the CTD database revealed chemicals that interact with these target genes, and the full protein-compound prediction results for each gene are available in the **Supplementary Materials** (**Supplementary Materials 14**-**18**). For the upregulated genes TUFM (**Supplementary Material 14**), MAST3 (**Supplementary Material 15**), and FNBP4 (**Supplementary Material 16**), we identified 15, 31, and 39 compounds that respectively inhibited their expression. Conversely, for the downregulated genes SH3YL1 (**Supplementary Material 17**) and SGF29 (**Supplementary Material 18**), we found 30 and 29 compounds that respectively stimulated their expression. Significantly, bisphenol A stimulated the downregulated gene SH3YL1 at both mRNA and protein levels, whereas rotenone demonstrated dual effects by stimulating the downregulated gene SGF29 and inhibiting the upregulated genes TUFM/FNBP4. Distinct pathways encompassed titanium dioxide-mediated methylation (MAST3) and ivermectin-induced protein inhibition (FNBP4) (**Supplementary Materials 14**-**18**). These chemicals present promising tools for precise gene manipulation.

## 4 Discussion

This work comprehensively evaluated the potential causative impact of gene expression during CD4+ T cell activation on the risk of OSA. Our integrated approach identified several candidate genes, with five major genes-TUFM, MAST3, FNBP4, SGF29, and SH3YL1-showing consistent causal evidence from both MR analysis and experimental validation. This convergence suggests that OSA risk may operate through interconnected pathways crucial for T cell activation, including mitochondrial regulation (TUFM), cytoskeletal dynamics (MAST3, FNBP4), and transcriptional control (SGF29, SH3YL1). These findings offer significant insights into the immune regulatory systems and prospective treatment targets of OSA, while also paving the way for new research avenues to comprehend the molecular pathophysiological mechanisms of sleep-disordered breathing.

Recent research indicates that the immune system significantly influences the incidence and progression of OSA. Patients with OSA demonstrate marked systemic inflammatory responses, characterized by elevated levels of pro-inflammatory substances, including C-reactive protein, interleukin-6 (IL-6), and tumor necrosis factor-alpha (TNF-α), alongside diminished levels of the anti-inflammatory marker IL-10 (12, 13). CD4^+^ T cells, integral to adaptive immunity, significantly contribute to the inflammatory process in OSA. IH stimulates CD4^+^ T cells and monocytes, resulting in the overproduction of proinflammatory cytokines (14, 15). Our research findings further investigate the function of CD4 This study generated single-cell transcriptomic and genotypic data^+^ T cells in OSA and affirm their significant role in the pathogenesis of OSA through gene regulatory mechanisms.

The TUFM gene encodes the mitochondrial translation elongation factor Tu, an essential regulatory component in mitochondrial protein synthesis (16). Mitochondrial dysfunction is regarded as a primary mechanism contributing to cellular damage induced by IH. IH results in diminished activity of mitochondrial respiratory chain complexes and an overproduction of reactive oxygen species (17), alterations that are intimately linked to systemic inflammatory responses in individuals with OSA. Variations in TUFM expression levels may be intricately related to mitochondrial autophagy and susceptibility to hypoxia stress (18). Our data indicate that alterations in TUFM expression in CD4^+^ T cells may provide an important biological foundation for immunological dysfunction in OSA patients, offering a theoretical framework for the development of therapeutic options targeting mitochondrial function in OSA.

MAST3 is a member of the MAST kinase family and participates in various physiological signaling pathways. This kinase family is essential for the regulation of cell proliferation, differentiation, and stress responses (19). The precise roles of MAST3 in the immune system remain unclear; nevertheless, alterations in its expression during T cell activation imply its potential role in regulating immunological responses (20). This study is the inaugural investigation to associate MAST3 with OSA, thus paving the way for additional research into the gene’s involvement in sleep-disordered breathing. MAST3 may engage in OSA-related inflammatory processes by modulating the activation state and activity of T cells.

FNBP4 regulates the actin cytoskeleton and is essential for preserving cell shape and migration (21). The formin protein family is crucial for the migration and polarization of immune cells (22), which is vital for the initiation and sustenance of inflammatory reactions. In the pathological process of OSA, the recruitment and infiltration of inflammatory cells into respiratory tract tissues are significant pathogenic characteristics (23). Patients with OSA demonstrate extensive inflammatory cell infiltration in the tissues of the upper respiratory tract (24), which correlates with the severity of the condition. Our findings indicate that FNBP4 may be involved in the inflammatory process of OSA by modulating the migratory ability of CD4^+^ T cells.

SGF29 is a component of the SAGA transcription co-activator complex, participating in histone modification and transcriptional regulation. This protein is involved in the activation of gene transcription by detecting histone H3K4me2/3 modifications (25) and is essential in cellular stress responses and the regulation of inflammatory gene expression. SH3YL1 possesses an SH3 domain, integral to protein-protein interactions and significant in signal transduction and inflammatory responses (26, 27). The precise activities of these two genes in OSA are ambiguous; nonetheless, their involvement in transcriptional regulation and signal transduction implies a potential role in the modulation of OSA-related gene expression programs.

Conventional eQTL studies predominantly utilize cells or tissues in a quiescent state, failing to capture the dynamic alterations in cellular function during disease states. The eQTL landscape of immune cells under stimulatory settings is distinctive, featuring numerous eQTLs identifiable solely under specific stimulatory circumstances. The context-specific CD4^+^ T cell eQTL methodology utilized can detect temporal variations in gene regulation during cellular activation (10), which is very pertinent for comprehending disorders like OSA that are characterized by persistent inflammatory activation. Our investigation identified genes associated with OSA and highlighted their differential expression patterns at various time points during T cell activation. This time-resolved research elucidates the molecular mechanisms behind the clinical progression of OSA, spanning from acute hypoxia episodes to chronic inflammatory injury.

The five key genes we identified present novel opportunities for individualized management of OSA. Utilizing a patient’s genotype and expression profile may enable the prediction of their response to various treatment regimens. Patients exhibiting diminished TUFM expression levels may be more susceptible to mitochondrial dysfunction and, hence, may derive greater benefit from antioxidant therapy. This gene-targeted therapeutic approach has been extensively utilized in oncology, and its implementation in sleep medicine presents encouraging potential. Small-molecule compounds that target the MAST3 signaling system may aid in regulating inflammation associated with OSA. Research indicates that specific anti-inflammatory medications may alleviate symptoms in patients with OSA (28, 29); however, the processes involved remain ambiguous. Our research offers genetic evidence corroborating the mechanism of action for these medications. The identified genes may function as both therapeutic targets and biomarkers for the diagnosis and prognosis of OSA. Currently, the diagnosis of OSA predominantly relies on polysomnography (30), which is accurate but expensive and impractical for large-scale screening. Establishing blood-based detection approaches utilizing gene expression or protein levels would markedly enhance the early detection rate of OSA.

A key limitation of our study is its reliance on data from individuals of predominantly European ancestry, which may limit the generalizability of our findings. Therefore, future research should concentrate on the following domains: first, it is imperative to validate the correlation between the five key genes identified and OSA across diverse racial populations to evaluate their universality; second, functional experiments must be undertaken to elucidate the specific regulatory mechanisms and interaction networks of these genes under IH conditions; third, OSA animal models should be developed based on these molecular targets to evaluate the therapeutic efficacy of targeted interventions; Finally, the clinical significance of these genes as diagnostic biomarkers and therapeutic targets should be confirmed in extensive clinical cohorts.

## Data Availability

The original contributions presented in the study are included in the article/**Supplementary Material**. Further inquiries can be directed to the corresponding author.
