# Supplementary material for: Dynamic genetic regulation of CD4+ T cells in obstructive sleep apnea: integrating context-specific eQTL, Mendelian randomization, single-cell sequencing, and experimental validation
Source: Front Immunol. 2025 Dec 17;16:1691347. doi: 10.3389/fimmu.2025.1691347 (PMC12753881; doi:10.3389/fimmu.2025.1691347)

Trait	Method	nSNP	pval	FDR	OR (95% CI)
MAD1L1_TEM_5d	Wald ratio	1	<0.001	0.002	1.040 (1.022 − 1.057)
SLC4A7_TN_IFN_5d	Wald ratio	1	0.002	0.041	0.976 (0.960 − 0.991)
SH3YL1_CD4_Memory_stim_16h	Wald ratio	1	<0.001	0.018	0.976 (0.963 − 0.989)
SH3YL1_CD4_Memory_stim_40h	Wald ratio	1	<0.001	0.007	0.975 (0.963 − 0.988)
SH3YL1_CD4_Memory_stim_5d	Wald ratio	1	<0.001	0.018	0.983 (0.973 − 0.992)
SH3YL1_CD4_Memory_uns_0h	Wald ratio	1	<0.001	0.018	0.987 (0.980 − 0.994)
SH3YL1_CD4_Naive_stim_16h	Wald ratio	1	<0.001	0.018	0.979 (0.967 − 0.990)
SH3YL1_CD4_Naive_stim_40h	Wald ratio	1	<0.001	0.007	0.977 (0.965 − 0.988)
SH3YL1_CD4_Naive_stim_5d	Wald ratio	1	<0.001	0.007	0.982 (0.972 − 0.991)
SH3YL1_CD4_Naive_uns_0h	Wald ratio	1	<0.001	0.018	0.987 (0.980 − 0.994)
SH3YL1_nTreg_40h	Wald ratio	1	<0.001	0.018	0.977 (0.964 − 0.990)
SH3YL1_T_ER−stress_5d	Wald ratio	1	<0.001	0.003	0.971 (0.957 − 0.984)
SH3YL1_TCM_0h	Wald ratio	1	<0.001	0.018	0.986 (0.979 − 0.994)
SH3YL1_TCM_16h	Wald ratio	1	<0.001	0.014	0.979 (0.968 − 0.990)
SH3YL1_TCM_40h	Wald ratio	1	<0.001	0.007	0.977 (0.966 − 0.989)
SH3YL1_TCM_5d	Wald ratio	1	<0.001	0.018	0.982 (0.973 − 0.992)
SH3YL1_TCM_LA	Wald ratio	1	<0.001	0.018	0.971 (0.956 − 0.987)
SH3YL1_TEM_0h	Wald ratio	1	<0.001	0.018	0.985 (0.976 − 0.993)
SH3YL1_TEM_40h	Wald ratio	1	<0.001	0.007	0.970 (0.955 − 0.985)
SH3YL1_TEM_5d	Wald ratio	1	<0.001	0.006	0.981 (0.972 − 0.991)
SH3YL1_TN_0h	Wald ratio	1	<0.001	0.018	0.987 (0.980 − 0.994)
SH3YL1_TN_16h	Wald ratio	1	<0.001	0.004	0.970 (0.956 − 0.984)
SH3YL1_TN_40h	Wald ratio	1	<0.001	0.006	0.981 (0.971 − 0.990)
SH3YL1_TN_5d	Wald ratio	1	<0.001	0.007	0.982 (0.972 − 0.991)
SH3YL1_TN_cycling_5d	Wald ratio	1	<0.001	0.005	0.972 (0.959 − 0.986)
SH3YL1_TN_HSP_5d	Wald ratio	1	<0.001	0.007	0.983 (0.975 − 0.992)
SH3YL1_TN_IFN_40h	Wald ratio	1	<0.001	0.007	0.976 (0.964 − 0.988)
SH3YL1_TN_IFN_5d	Wald ratio	1	<0.001	0.007	0.975 (0.963 − 0.988)
SH3YL1_TN_LA	Wald ratio	1	0.001	0.022	0.971 (0.954 − 0.987)
GPC1_CD4_Memory_stim_16h	Wald ratio	1	0.001	0.027	0.980 (0.969 − 0.992)
GPC1_CD4_Memory_stim_40h	Wald ratio	1	0.001	0.019	0.978 (0.966 − 0.991)
GPC1_CD4_Memory_stim_5d	Wald ratio	1	0.001	0.032	0.977 (0.963 − 0.991)
GPC1_TCM_16h	Wald ratio	1	0.001	0.032	0.979 (0.967 − 0.992)
GPC1_TCM_40h	Wald ratio	1	0.001	0.030	0.973 (0.957 − 0.989)
GPC1_TCM_5d	Wald ratio	1	0.001	0.032	0.979 (0.966 − 0.992)
GPC1_TEM_40h	Wald ratio	1	0.001	0.019	0.975 (0.960 − 0.989)
GPC1_TEM_5d	Wald ratio	1	0.001	0.027	0.974 (0.959 − 0.989)
GPC1_TN_cycling_40h	Wald ratio	1	0.001	0.027	0.978 (0.965 − 0.991)
MAST2_TN_40h	Wald ratio	1	<0.001	0.001	1.024 (1.014 − 1.035)
PABPC4_CD4_Memory_stim_16h	Wald ratio	1	<0.001	0.001	0.944 (0.922 − 0.966)
PABPC4_CD4_Memory_stim_5d	Wald ratio	1	<0.001	0.003	0.953 (0.932 − 0.975)
PABPC4_CD4_Naive_stim_40h	Wald ratio	1	<0.001	0.001	0.916 (0.883 − 0.949)
IL12RB1_TN_cycling_5d	Wald ratio	1	0.001	0.027	0.983 (0.972 − 0.993)
MAST3_CD4_Memory_stim_40h	Wald ratio	1	<0.001	<0.001	1.049 (1.030 − 1.068)
MAST3_CD4_Memory_uns_0h	Wald ratio	1	<0.001	<0.001	1.027 (1.017 − 1.037)
MAST3_CD4_Naive_stim_5d	Wald ratio	1	<0.001	<0.001	1.031 (1.019 − 1.043)
MAST3_CD4_Naive_uns_0h	Wald ratio	1	<0.001	<0.001	1.021 (1.013 − 1.030)
MAST3_TCM_40h	Wald ratio	1	<0.001	<0.001	1.035 (1.021 − 1.049)
MAST3_TCM_5d	Wald ratio	1	<0.001	<0.001	1.029 (1.019 − 1.040)
MAST3_TN_0h	Wald ratio	1	<0.001	<0.001	1.024 (1.015 − 1.033)
MAST3_TN_40h	Wald ratio	1	<0.001	<0.001	1.037 (1.023 − 1.051)
GID8_TN_40h	Wald ratio	1	<0.001	0.009	1.036 (1.017 − 1.054)
ZC3HAV1_TN_5d	Wald ratio	1	0.001	0.019	1.025 (1.011 − 1.039)
ZC3HAV1_TN_HSP_5d	Wald ratio	1	0.001	0.019	1.040 (1.017 − 1.064)
PLEKHA1_CD4_Naive_uns_0h	Wald ratio	1	0.001	0.032	0.973 (0.957 − 0.989)
FNBP4_CD4_Memory_uns_0h	Wald ratio	1	<0.001	<0.001	1.044 (1.027 − 1.062)
FNBP4_CD4_Naive_uns_0h	Wald ratio	1	<0.001	<0.001	1.052 (1.031 − 1.072)
FNBP4_TN_0h	Wald ratio	1	<0.001	0.002	1.043 (1.024 − 1.063)
CARS1_TN_IFN_5d	Wald ratio	1	0.002	0.044	0.959 (0.934 − 0.985)
NIT2_TCM_5d	Wald ratio	1	0.001	0.027	0.962 (0.940 − 0.984)
DARS1_CD4_Memory_stim_40h	Wald ratio	1	0.001	0.029	0.913 (0.864 − 0.964)
DARS1_CD4_Memory_stim_5d	Wald ratio	1	0.001	0.027	0.969 (0.952 − 0.987)
DARS1_CD4_Naive_stim_16h	Wald ratio	1	0.001	0.029	0.976 (0.961 − 0.990)
DARS1_CD4_Naive_stim_40h	Wald ratio	1	<0.001	0.017	0.967 (0.949 − 0.985)
DARS1_TCM_40h	Wald ratio	1	<0.001	0.017	0.968 (0.951 − 0.985)
DARS1_TEM_40h	Wald ratio	1	<0.001	0.017	0.961 (0.940 − 0.982)
DARS1_TN_16h	Wald ratio	1	0.001	0.029	0.976 (0.962 − 0.990)
DARS1_TN_40h	Wald ratio	1	0.001	0.027	0.977 (0.964 − 0.991)
DARS1_TN_IFN_40h	Wald ratio	1	0.001	0.034	0.978 (0.964 − 0.991)
RAB29_CD4_Memory_stim_5d	Wald ratio	1	0.001	0.024	0.970 (0.953 − 0.987)
RAB29_CD4_Naive_stim_40h	Wald ratio	1	<0.001	0.014	0.966 (0.949 − 0.984)
RAB29_CD4_Naive_stim_5d	Wald ratio	1	<0.001	0.017	0.971 (0.956 − 0.987)
RAB29_TN_40h	Wald ratio	1	<0.001	0.014	0.968 (0.951 − 0.985)
PTBP2_CD4_Naive_stim_16h	Wald ratio	1	<0.001	0.014	0.974 (0.960 − 0.988)
PTBP2_CD4_Naive_stim_5d	Wald ratio	1	<0.001	0.015	0.974 (0.960 − 0.988)

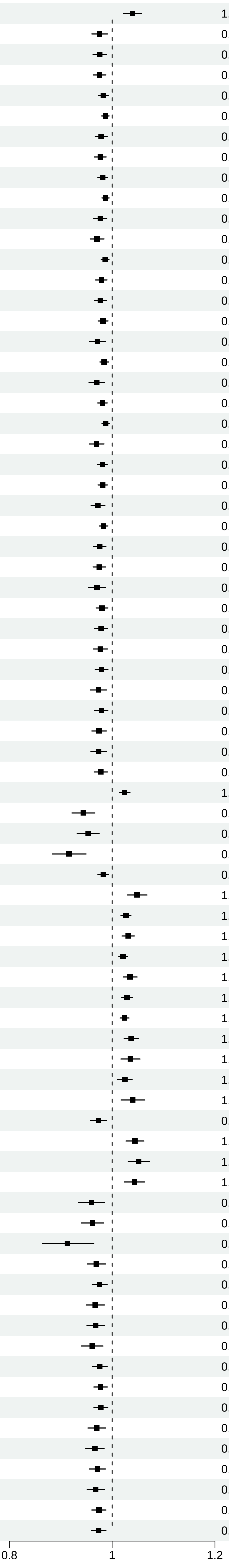

Supplement: Supplementary file 1 [file Supplementaryfile1.zip › Supplementary files/S1.pdf]
