# Supplementary material for: Dynamic genetic regulation of CD4+ T cells in obstructive sleep apnea: integrating context-specific eQTL, Mendelian randomization, single-cell sequencing, and experimental validation
Source: Front Immunol. 2025 Dec 17;16:1691347. doi: 10.3389/fimmu.2025.1691347 (PMC12753881; doi:10.3389/fimmu.2025.1691347)

Trait	Method	nSNP	*P*−Value	**OR (95% CI)**	FDR
FCGR2B	IVW	24	<0.001	1.032 (1.017 − 1.047)	0.003
FGFR1	IVW	6	<0.001	1.047 (1.020 − 1.073)	0.029
FLT1P1	IVW	7	0.001	1.022 (1.009 − 1.036)	0.044
FNBP4	IVW	2	<0.001	1.194 (1.097 − 1.300)	0.006
FRAT2	IVW	10	0.001	1.034 (1.014 − 1.054)	0.043
GDPD3	IVW	2	<0.001	1.111 (1.056 − 1.168)	0.006
GNA12	IVW	10	0.001	0.948 (0.918 − 0.979)	0.048
GPD2	IVW	6	<0.001	0.926 (0.890 − 0.963)	0.011
GTF2I	IVW	2	<0.001	1.074 (1.049 − 1.100)	<0.001
GUK1	IVW	2	<0.001	1.196 (1.089 − 1.314)	0.017
HARS2	IVW	2	<0.001	0.862 (0.809 − 0.918)	0.002
HIST1H3E	IVW	4	<0.001	1.060 (1.032 − 1.089)	0.004
HSD17B8	IVW	5	<0.001	0.946 (0.923 − 0.971)	0.004
HTR3A	IVW	2	0.001	0.847 (0.771 − 0.931)	0.035
IDUA	IVW	4	<0.001	0.937 (0.913 − 0.963)	0.001
IK	IVW	5	<0.001	0.932 (0.904 − 0.960)	0.002
ING5	IVW	11	<0.001	0.961 (0.940 − 0.981)	0.018
INVS	IVW	8	<0.001	1.064 (1.029 − 1.099)	0.020
ITIH2	IVW	5	0.001	0.944 (0.912 − 0.977)	0.049
ITK	IVW	5	<0.001	0.929 (0.891 − 0.968)	0.029
JAGN1	IVW	2	<0.001	0.939 (0.907 − 0.973)	0.030
KANSL1−AS1	IVW	13	<0.001	1.017 (1.010 − 1.025)	0.002
KDM5A	IVW	11	<0.001	1.060 (1.033 − 1.088)	0.003
KIAA0247	IVW	6	0.001	0.921 (0.878 − 0.965)	0.037
KIAA1407	IVW	3	<0.001	0.906 (0.858 − 0.956)	0.026
KPNB1	IVW	2	0.001	0.878 (0.813 − 0.948)	0.045
KRT2	IVW	2	<0.001	0.893 (0.839 − 0.950)	0.025
L3MBTL3	IVW	21	<0.001	1.039 (1.022 − 1.055)	0.002
LGR4	IVW	3	<0.001	0.883 (0.826 − 0.944)	0.021
LGSN	IVW	11	<0.001	1.045 (1.025 − 1.065)	0.003
LRRC37A2	IVW	11	<0.001	1.021 (1.013 − 1.029)	<0.001
LRRC37A4P	IVW	13	<0.001	0.985 (0.978 − 0.992)	0.004
LRRC56	IVW	8	0.001	0.967 (0.948 − 0.985)	0.034
LY6G5B	IVW	7	<0.001	0.946 (0.920 − 0.973)	0.011
LY6G5C	IVW	10	0.001	0.965 (0.945 − 0.986)	0.044
MACF1	IVW	9	<0.001	1.073 (1.046 − 1.101)	<0.001
MAP3K9	IVW	3	<0.001	1.063 (1.027 − 1.099)	0.030
MAP7D1	IVW	7	0.001	0.960 (0.936 − 0.983)	0.047
MAPK8IP3	IVW	2	<0.001	1.186 (1.085 − 1.297)	0.016
MIDN	IVW	2	<0.001	0.879 (0.823 − 0.939)	0.013
MLF1IP	IVW	4	<0.001	0.972 (0.958 − 0.987)	0.016
MPV17L2	IVW	3	<0.001	1.157 (1.075 − 1.245)	0.011
MTL5	IVW	5	0.001	1.044 (1.018 − 1.072)	0.048
MYO1F	IVW	2	0.001	0.890 (0.833 − 0.951)	0.035
N4BP2	IVW	15	<0.001	1.054 (1.027 − 1.081)	0.007
NCF1	IVW	5	<0.001	1.089 (1.051 − 1.129)	0.001
NCOA3	IVW	7	0.001	1.068 (1.028 − 1.109)	0.036
NDUFA2	IVW	8	<0.001	1.049 (1.028 − 1.069)	0.001
NDUFS5	IVW	22	0.001	1.040 (1.016 − 1.063)	0.041
NFKBIA	IVW	6	<0.001	0.952 (0.930 − 0.976)	0.009
NMI	IVW	3	0.001	1.048 (1.020 − 1.078)	0.044
NPDC1	IVW	5	<0.001	1.033 (1.015 − 1.053)	0.031
NPIP	IVW	16	<0.001	0.982 (0.973 − 0.991)	0.017
NUCKS1	IVW	6	<0.001	1.045 (1.022 − 1.068)	0.009
OARD1	IVW	2	0.001	1.240 (1.095 − 1.403)	0.038
PABPC4	IVW	2	<0.001	0.880 (0.838 − 0.924)	<0.001
PHF13	IVW	4	<0.001	0.850 (0.801 − 0.902)	<0.001
PLEC	IVW	4	<0.001	0.949 (0.921 − 0.977)	0.029
PODXL2	IVW	6	<0.001	0.929 (0.894 − 0.965)	0.015
PPAPDC2	IVW	9	<0.001	1.048 (1.026 − 1.070)	0.003
PPIP5K2	IVW	14	<0.001	1.024 (1.011 − 1.037)	0.020
PRIM1	IVW	3	<0.001	1.112 (1.058 − 1.169)	0.005
PRKCH	IVW	4	<0.001	0.874 (0.811 − 0.942)	0.028
PSEN1	IVW	6	<0.001	0.954 (0.929 − 0.978)	0.023
PTPMT1	IVW	2	<0.001	0.832 (0.768 − 0.901)	0.002

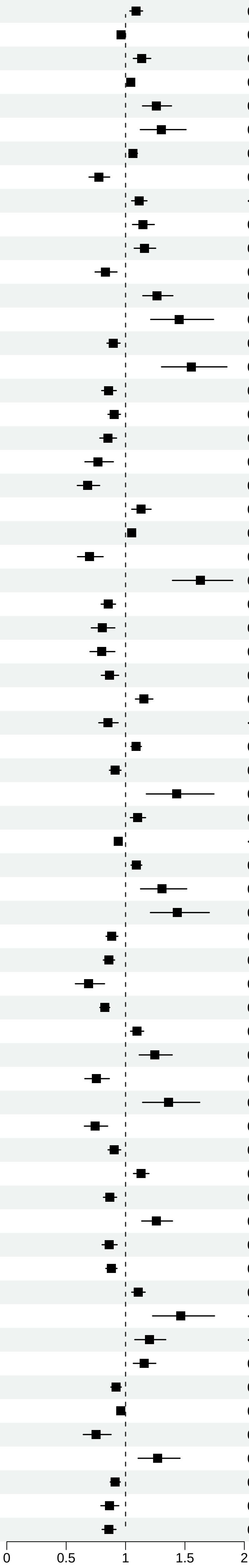

Supplement: Supplementary file 1 [file Supplementaryfile1.zip › Supplementary files/S10.pdf]
