# Supplementary material for: Dynamic genetic regulation of CD4+ T cells in obstructive sleep apnea: integrating context-specific eQTL, Mendelian randomization, single-cell sequencing, and experimental validation
Source: Front Immunol. 2025 Dec 17;16:1691347. doi: 10.3389/fimmu.2025.1691347 (PMC12753881; doi:10.3389/fimmu.2025.1691347)

| | | | | | |
|---|---|---|---|---|---|
| SH3YL1_nk | SH3YL1 | Wald ratio | 1 | <0.001 | 1.062 (1.030 − 1.094) |
| SHFM1_cd4nc | SHFM1 | Wald ratio | 1 | 0.001 | 1.045 (1.019 − 1.072) |
| SHFM1_cd8et | SHFM1 | Wald ratio | 1 | 0.001 | 1.042 (1.017 − 1.067) |
| SHFM1_nk | SHFM1 | Wald ratio | 1 | 0.001 | 1.054 (1.022 − 1.086) |
| SPNS1_cd4nc | SPNS1 | Wald ratio | 1 | 0.002 | 0.960 (0.936 − 0.985) |
| SPNS1_nk | SPNS1 | Wald ratio | 1 | 0.002 | 0.952 (0.923 − 0.981) |
| STOX1_cd4nc | STOX1 | Wald ratio | 1 | <0.001 | 0.962 (0.944 − 0.979) |
| STOX1_cd8nc | STOX1 | Wald ratio | 1 | <0.001 | 1.041 (1.023 − 1.059) |
| SULT1A1_mononc | SULT1A1 | Wald ratio | 1 | 0.001 | 1.056 (1.022 − 1.090) |
| TAPBP_bin | TAPBP | Wald ratio | 1 | <0.001 | 0.954 (0.930 − 0.978) |
| TAPBP_bmem | TAPBP | Wald ratio | 1 | <0.001 | 0.942 (0.912 − 0.973) |
| TAPBP_cd4nc | TAPBP | Wald ratio | 1 | <0.001 | 0.970 (0.954 − 0.986) |
| TAPBP_cd8et | TAPBP | Wald ratio | 1 | <0.001 | 0.943 (0.913 − 0.974) |
| TAPBP_cd8nc | TAPBP | Wald ratio | 1 | <0.001 | 0.955 (0.931 − 0.979) |
| TAPBP_nk | TAPBP | Wald ratio | 1 | <0.001 | 0.923 (0.890 − 0.958) |
| TECPR1_cd4nc | TECPR1 | Wald ratio | 1 | <0.001 | 1.054 (1.027 − 1.083) |
| TMEM204_bin | TMEM204 | Wald ratio | 1 | <0.001 | 0.943 (0.918 − 0.968) |
| TMEM204_cd4et | TMEM204 | Wald ratio | 1 | <0.001 | 0.950 (0.924 − 0.976) |
| TMEM204_cd4nc | TMEM204 | Wald ratio | 1 | <0.001 | 0.962 (0.945 − 0.979) |
| TMEM204_cd8et | TMEM204 | Wald ratio | 1 | <0.001 | 0.967 (0.953 − 0.982) |
| TMEM204_cd8nc | TMEM204 | Wald ratio | 1 | <0.001 | 0.951 (0.926 − 0.976) |
| TMEM204_cd8s100b | TMEM204 | Wald ratio | 1 | <0.001 | 0.931 (0.897 − 0.967) |
| TMEM204_nk | TMEM204 | Wald ratio | 1 | <0.001 | 0.969 (0.955 − 0.983) |
| TMEM45B_cd4nc | TMEM45B | Wald ratio | 1 | <0.001 | 1.034 (1.020 − 1.049) |
| TNFRSF14_cd4et | TNFRSF14 | Wald ratio | 1 | 0.002 | 0.944 (0.911 − 0.979) |
| TNFRSF14_cd4nc | TNFRSF14 | Wald ratio | 1 | <0.001 | 0.959 (0.940 − 0.978) |
| TNFRSF14_cd8et | TNFRSF14 | Wald ratio | 1 | <0.001 | 0.949 (0.922 − 0.977) |
| TNFRSF14_cd8nc | TNFRSF14 | Wald ratio | 1 | <0.001 | 0.941 (0.915 − 0.969) |
| TNFRSF14_nk | TNFRSF14 | Wald ratio | 1 | 0.001 | 0.951 (0.924 − 0.978) |
| TNRC6A_cd4nc | TNRC6A | Wald ratio | 1 | <0.001 | 1.075 (1.038 − 1.113) |
| TPK1_cd4nc | TPK1 | Wald ratio | 1 | 0.001 | 0.949 (0.919 − 0.979) |
| TRAPPC2L_cd4nc | TRAPPC2L | Wald ratio | 1 | <0.001 | 0.968 (0.953 − 0.984) |
| TRAPPC2L_cd8et | TRAPPC2L | Wald ratio | 1 | <0.001 | 0.955 (0.934 − 0.977) |
| TRAPPC2L_cd8nc | TRAPPC2L | Wald ratio | 1 | <0.001 | 0.954 (0.933 − 0.976) |
| TTC12_cd8s100b | TTC12 | Wald ratio | 1 | 0.001 | 1.035 (1.014 − 1.057) |
| TUFM_bin | TUFM | Wald ratio | 1 | <0.001 | 0.974 (0.963 − 0.985) |
| TUFM_bmem | TUFM | Wald ratio | 1 | <0.001 | 0.966 (0.951 − 0.980) |
| TUFM_cd4et | TUFM | Wald ratio | 1 | <0.001 | 0.968 (0.955 − 0.982) |
| TUFM_cd4nc | TUFM | Wald ratio | 1 | <0.001 | 0.981 (0.973 − 0.989) |
| TUFM_cd8et | TUFM | Wald ratio | 1 | <0.001 | 0.974 (0.963 − 0.985) |
| TUFM_cd8nc | TUFM | Wald ratio | 1 | <0.001 | 0.974 (0.962 − 0.985) |
| TUFM_cd8s100b | TUFM | Wald ratio | 1 | <0.001 | 0.956 (0.938 − 0.975) |
| TUFM_dc | TUFM | Wald ratio | 1 | <0.001 | 0.955 (0.936 − 0.974) |
| TUFM_monoc | TUFM | Wald ratio | 1 | <0.001 | 0.953 (0.933 − 0.973) |
| TUFM_mononc | TUFM | Wald ratio | 1 | <0.001 | 0.953 (0.933 − 0.973) |
| TUFM_nk | TUFM | Wald ratio | 1 | <0.001 | 0.968 (0.954 − 0.982) |
| UBXN6_cd4nc | UBXN6 | Wald ratio | 1 | <0.001 | 1.047 (1.023 − 1.071) |
| UBXN6_cd8et | UBXN6 | Wald ratio | 1 | 0.001 | 1.052 (1.022 − 1.083) |
| UBXN6_cd8nc | UBXN6 | Wald ratio | 1 | <0.001 | 1.063 (1.031 − 1.097) |
| UQCRH_bin | UQCRH | Wald ratio | 1 | <0.001 | 1.057 (1.027 − 1.087) |
| UQCRH_bmem | UQCRH | Wald ratio | 1 | <0.001 | 1.056 (1.031 − 1.083) |
| UQCRH_cd4et | UQCRH | Wald ratio | 1 | <0.001 | 1.050 (1.024 − 1.077) |
| UQCRH_cd4nc | UQCRH | Wald ratio | 1 | <0.001 | 1.028 (1.013 − 1.042) |
| UQCRH_cd8et | UQCRH | Wald ratio | 1 | <0.001 | 1.037 (1.018 − 1.056) |
| UQCRH_cd8nc | UQCRH | Wald ratio | 1 | <0.001 | 1.035 (1.017 − 1.053) |
| UQCRH_nk | UQCRH | Wald ratio | 1 | <0.001 | 1.047 (1.023 − 1.072) |
| USP36_cd4nc | USP36 | Wald ratio | 1 | 0.001 | 0.952 (0.926 − 0.979) |
| USP36_cd8nc | USP36 | Wald ratio | 1 | 0.001 | 0.945 (0.913 − 0.979) |
| ZFAND3_nk | ZFAND3 | Wald ratio | 1 | 0.001 | 1.061 (1.023 − 1.101) |
| ZKSCAN3_monoc | ZKSCAN3 | Wald ratio | 1 | <0.001 | 0.936 (0.905 − 0.969) |
| ZKSCAN8_nkr | ZKSCAN8 | Wald ratio | 1 | <0.001 | 1.051 (1.023 − 1.080) |
| ZNF266_cd8et | ZNF266 | Wald ratio | 1 | 0.001 | 0.952 (0.924 − 0.981) |
| ZNF419_cd4nc | ZNF419 | Wald ratio | 1 | <0.001 | 1.079 (1.040 − 1.120) |
| ZNF568_cd4nc | ZNF568 | Wald ratio | 1 | <0.001 | 0.946 (0.923 − 0.969) |
| CD53_cd4nc | CD53 | IVW | 2 | <0.001 | 0.965 (0.946 − 0.984) |
| EBPL_bmem | EBPL | IVW | 2 | 0.001 | 1.034 (1.013 − 1.055) |
| EBPL_cd4et | EBPL | IVW | 2 | 0.001 | 1.022 (1.009 − 1.034) |
| EBPL_cd4nc | EBPL | IVW | 3 | 0.001 | 1.014 (1.006 − 1.023) |
| EBPL_cd8et | EBPL | IVW | 3 | <0.001 | 1.022 (1.010 − 1.033) |
| EBPL_cd8nc | EBPL | IVW | 2 | 0.001 | 1.019 (1.008 − 1.030) |
| EBPL_nk | EBPL | IVW | 2 | 0.001 | 1.022 (1.009 − 1.035) |
| EGFL8_cd8et | EGFL8 | IVW | 3 | <0.001 | 0.964 (0.950 − 0.978) |
| HCG25_cd8nc | HCG25 | IVW | 2 | <0.001 | 1.035 (1.017 − 1.053) |
| HLA−DPA1_bin | HLA−DPA1 | IVW | 3 | <0.001 | 0.974 (0.961 − 0.986) |
| HLA−DPB1_bin | HLA−DPB1 | IVW | 2 | 0.001 | 0.981 (0.970 − 0.992) |
| HLA−DQA1_nk | HLA−DQA1 | IVW | 2 | 0.001 | 1.048 (1.020 − 1.078) |
| HLA−DQA2_cd8nc | HLA−DQA2 | IVW | 2 | 0.001 | 0.973 (0.959 − 0.988) |
| HLA−DQA2_mononc | HLA−DQA2 | IVW | 3 | <0.001 | 1.017 (1.008 − 1.027) |
| ZBTB9_cd8s100b | ZBTB9 | IVW | 3 | <0.001 | 1.026 (1.012 − 1.041) |
| ZNF165_cd8et | ZNF165 | IVW | 2 | <0.001 | 1.035 (1.019 − 1.050) |

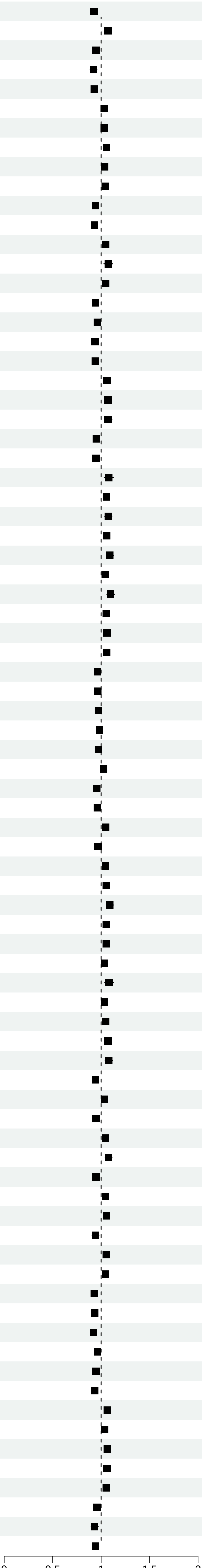

Supplement: Supplementary file 1 [file Supplementaryfile1.zip › Supplementary files/S15.pdf]
