# Supplementary material for: Dynamic genetic regulation of CD4+ T cells in obstructive sleep apnea: integrating context-specific eQTL, Mendelian randomization, single-cell sequencing, and experimental validation
Source: Front Immunol. 2025 Dec 17;16:1691347. doi: 10.3389/fimmu.2025.1691347 (PMC12753881; doi:10.3389/fimmu.2025.1691347)

Trait	Method	nSNP	pval	FDR	OR (95% CI)
KANSL1_CD4_Memory_stim_5d	Wald ratio	1	<0.001	0.002	1.030 (1.016 − 1.044)
KANSL1_CD4_Memory_uns_0h	Wald ratio	1	<0.001	0.002	1.032 (1.018 − 1.046)
KANSL1_CD4_Naive_stim_5d	Wald ratio	1	<0.001	0.002	1.024 (1.013 − 1.035)
KANSL1_CD4_Naive_uns_0h	Wald ratio	1	<0.001	0.002	1.029 (1.016 − 1.042)
KANSL1_T_ER−stress_5d	Wald ratio	1	<0.001	0.002	1.028 (1.015 − 1.040)
KANSL1_TCM_0h	Wald ratio	1	<0.001	0.002	1.028 (1.016 − 1.041)
KANSL1_TCM_5d	Wald ratio	1	<0.001	0.002	1.033 (1.018 − 1.048)
KANSL1_TN_0h	Wald ratio	1	<0.001	0.002	1.029 (1.016 − 1.042)
KANSL1_TN_5d	Wald ratio	1	<0.001	0.002	1.028 (1.015 − 1.041)
KANSL1_TN_HSP_5d	Wald ratio	1	<0.001	0.002	1.026 (1.014 − 1.038)
KANSL1_TN_IFN_5d	Wald ratio	1	<0.001	0.002	1.046 (1.025 − 1.068)
WASHC3_CD4_Memory_stim_40h	Wald ratio	1	<0.001	0.002	1.108 (1.057 − 1.161)
WASHC3_CD4_Naive_stim_16h	Wald ratio	1	0.001	0.027	1.026 (1.011 − 1.042)
WASHC3_CD4_Naive_stim_40h	Wald ratio	1	0.001	0.027	1.033 (1.013 − 1.053)
WASHC3_TN_40h	Wald ratio	1	0.001	0.027	1.034 (1.014 − 1.054)
EBPL_CD4_Memory_stim_16h	Wald ratio	1	0.002	0.041	0.987 (0.979 − 0.995)
EBPL_CD4_Memory_stim_40h	Wald ratio	1	0.002	0.041	0.964 (0.942 − 0.987)
EBPL_CD4_Memory_stim_5d	Wald ratio	1	0.002	0.041	0.982 (0.971 − 0.993)
EBPL_CD4_Memory_uns_0h	Wald ratio	1	0.002	0.041	0.985 (0.976 − 0.995)
EBPL_CD4_Naive_stim_5d	Wald ratio	1	0.002	0.041	0.984 (0.974 − 0.994)
EBPL_CD4_Naive_uns_0h	Wald ratio	1	0.002	0.047	0.984 (0.974 − 0.994)
EBPL_TCM_0h	Wald ratio	1	0.002	0.041	0.985 (0.976 − 0.995)
EBPL_TCM_40h	Wald ratio	1	0.002	0.041	0.983 (0.973 − 0.994)
EBPL_TCM_5d	Wald ratio	1	0.002	0.041	0.982 (0.970 − 0.993)
EBPL_TEM_0h	Wald ratio	1	0.002	0.041	0.983 (0.973 − 0.994)
EBPL_TEM_40h	Wald ratio	1	0.002	0.041	0.979 (0.966 − 0.992)
EBPL_TM_ER−stress_40h	Wald ratio	1	0.002	0.036	0.974 (0.958 − 0.990)
EBPL_TN_0h	Wald ratio	1	0.002	0.047	0.984 (0.973 − 0.994)
EBPL_TN_16h	Wald ratio	1	0.002	0.047	0.982 (0.971 − 0.994)
EBPL_TN_cycling_5d	Wald ratio	1	0.002	0.041	0.980 (0.967 − 0.992)
EBPL_TN_HSP_5d	Wald ratio	1	0.002	0.041	0.980 (0.968 − 0.993)
EBPL_TN_IFN_5d	Wald ratio	1	0.001	0.030	0.979 (0.967 − 0.992)
NMI_CD4_Memory_stim_16h	Wald ratio	1	0.001	0.019	1.042 (1.018 − 1.066)
NMI_CD4_Memory_stim_40h	Wald ratio	1	<0.001	0.018	1.049 (1.021 − 1.078)
NMI_CD4_Memory_stim_5d	Wald ratio	1	<0.001	0.018	1.044 (1.019 − 1.069)
NMI_CD4_Naive_stim_16h	Wald ratio	1	<0.001	0.018	1.045 (1.020 − 1.071)
NMI_CD4_Naive_uns_0h	Wald ratio	1	<0.001	0.018	1.023 (1.010 − 1.036)
NMI_TCM_16h	Wald ratio	1	<0.001	0.015	1.028 (1.013 − 1.044)
NMI_TEM_40h	Wald ratio	1	<0.001	0.018	1.019 (1.008 − 1.030)
NMI_TN_40h	Wald ratio	1	0.001	0.028	1.028 (1.011 − 1.044)
SRSF6_CD4_Memory_stim_40h	Wald ratio	1	0.002	0.047	1.063 (1.022 − 1.106)
SRSF6_TCM_40h	Wald ratio	1	0.002	0.047	1.029 (1.010 − 1.048)
SRSF6_TN_40h	Wald ratio	1	0.002	0.047	1.029 (1.010 − 1.048)
PEPD_CD4_Naive_stim_40h	Wald ratio	1	<0.001	0.017	1.025 (1.011 − 1.040)
PEPD_TN_16h	Wald ratio	1	<0.001	0.017	1.025 (1.011 − 1.038)
PEPD_TN_40h	Wald ratio	1	<0.001	0.017	1.026 (1.012 − 1.041)
RPS10_HSP_16h	Wald ratio	1	<0.001	0.018	1.025 (1.011 − 1.038)
RPS10_TN_cycling_40h	Wald ratio	1	0.001	0.022	1.021 (1.009 − 1.034)
PGPEP1_CD4_Memory_stim_40h	Wald ratio	1	<0.001	0.003	1.044 (1.023 − 1.066)
TMEM204_CD4_Memory_uns_0h	Wald ratio	1	<0.001	0.002	1.024 (1.013 − 1.034)
TMEM204_CD4_Naive_uns_0h	Wald ratio	1	<0.001	0.002	1.025 (1.014 − 1.037)
TMEM204_TCM_0h	Wald ratio	1	<0.001	0.002	1.023 (1.013 − 1.034)
TMEM204_TN_0h	Wald ratio	1	<0.001	0.002	1.023 (1.013 − 1.034)
MYH11_CD4_Memory_uns_0h	Wald ratio	1	0.002	0.045	0.985 (0.976 − 0.995)
MYH11_TCM_0h	Wald ratio	1	0.002	0.045	0.980 (0.967 − 0.993)
MYH11_TEM_0h	Wald ratio	1	0.002	0.045	0.981 (0.968 − 0.993)
MYH11_TN_0h	Wald ratio	1	0.002	0.045	0.984 (0.973 − 0.994)
LRRC1_CD4_Naive_stim_5d	Wald ratio	1	<0.001	0.002	1.027 (1.015 − 1.040)
FAM117B_CD4_Memory_uns_0h	Wald ratio	1	0.001	0.034	0.982 (0.971 − 0.993)
FAM117B_CD4_Naive_uns_0h	Wald ratio	1	<0.001	0.010	0.983 (0.974 − 0.992)
FAM117B_TN_16h	Wald ratio	1	<0.001	0.010	0.976 (0.964 − 0.989)
SAT2_CD4_Memory_uns_0h	Wald ratio	1	0.002	0.042	0.979 (0.966 − 0.992)
SAT2_TN_0h	Wald ratio	1	0.001	0.032	0.977 (0.963 − 0.991)
ARHGEF19_CD4_Memory_stim_40h	Wald ratio	1	0.001	0.034	0.983 (0.973 − 0.994)
ARHGEF19_CD4_Memory_stim_5d	Wald ratio	1	0.002	0.036	0.984 (0.975 − 0.994)
ARHGEF19_CD4_Naive_stim_16h	Wald ratio	1	0.002	0.036	0.983 (0.973 − 0.994)
ARHGEF19_TEM_40h	Wald ratio	1	0.001	0.034	0.982 (0.971 − 0.993)
ARHGEF19_TN_16h	Wald ratio	1	0.001	0.034	0.985 (0.976 − 0.994)
ARHGEF19_TN_40h	Wald ratio	1	0.001	0.034	0.987 (0.979 − 0.995)
PPIP5K2_TN_16h	Wald ratio	1	<0.001	0.008	0.962 (0.943 − 0.981)
PPIP5K2_TN_40h	Wald ratio	1	<0.001	0.008	0.966 (0.949 − 0.983)
C4orf33_CD4_Memory_stim_5d	Wald ratio	1	0.001	0.027	0.976 (0.962 − 0.990)
C4orf33_CD4_Naive_stim_5d	Wald ratio	1	<0.001	0.005	0.968 (0.952 − 0.984)
C4orf33_TCM_5d	Wald ratio	1	0.001	0.027	0.976 (0.962 − 0.990)
C4orf33_TEM_5d	Wald ratio	1	<0.001	0.008	0.967 (0.950 − 0.984)

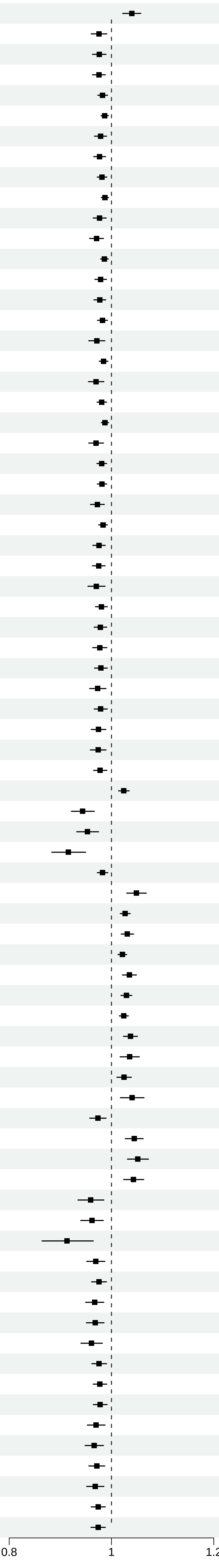

Supplement: Supplementary file 1 [file Supplementaryfile1.zip › Supplementary files/S2.pdf]
