# Supplementary material for: Dynamic genetic regulation of CD4+ T cells in obstructive sleep apnea: integrating context-specific eQTL, Mendelian randomization, single-cell sequencing, and experimental validation
Source: Front Immunol. 2025 Dec 17;16:1691347. doi: 10.3389/fimmu.2025.1691347 (PMC12753881; doi:10.3389/fimmu.2025.1691347)

Trait	Method	nSNP	pval	FDR	OR (95% CI)
C4orf33_TN_5d	Wald ratio	1	<0.001	0.017	0.970 (0.954 − 0.986)
TMEM45B_CD4_Memory_stim_16h	Wald ratio	1	<0.001	0.007	0.958 (0.937 − 0.979)
TMEM45B_CD4_Memory_uns_0h	Wald ratio	1	<0.001	0.005	0.980 (0.971 − 0.990)
TMEM45B_CD4_Naive_stim_16h	Wald ratio	1	<0.001	0.006	0.959 (0.939 − 0.979)
TMEM45B_CD4_Naive_stim_5d	Wald ratio	1	<0.001	0.007	0.974 (0.961 − 0.987)
TMEM45B_CD4_Naive_uns_0h	Wald ratio	1	<0.001	0.007	0.973 (0.960 − 0.987)
TMEM45B_TCM_0h	Wald ratio	1	<0.001	0.007	0.981 (0.971 − 0.990)
TMEM45B_TN_0h	Wald ratio	1	<0.001	0.007	0.976 (0.963 − 0.988)
CENPU_TEM_HLApositive_5d	Wald ratio	1	<0.001	0.019	0.970 (0.953 − 0.987)
CENPU_TN_IFN_40h	Wald ratio	1	<0.001	0.019	0.969 (0.952 − 0.986)
MIDEAS_CD4_Memory_stim_16h	Wald ratio	1	0.002	0.049	0.985 (0.975 − 0.995)
MIDEAS_CD4_Naive_stim_40h	Wald ratio	1	0.002	0.041	0.979 (0.966 − 0.992)
MIDEAS_TCM_5d	Wald ratio	1	0.002	0.041	0.983 (0.972 − 0.994)
MIDEAS_TEM_16h	Wald ratio	1	0.002	0.049	0.970 (0.951 − 0.989)
MIDEAS_TN_40h	Wald ratio	1	0.002	0.049	0.981 (0.969 − 0.993)
MED19_CD4_Naive_stim_16h	Wald ratio	1	0.002	0.038	0.974 (0.959 − 0.990)
TNFRSF14_CD4_Memory_stim_40h	Wald ratio	1	0.001	0.034	1.069 (1.026 − 1.114)
TNFRSF14_CD4_Memory_stim_5d	Wald ratio	1	0.001	0.019	1.029 (1.013 − 1.046)
TNFRSF14_CD4_Naive_stim_40h	Wald ratio	1	0.002	0.042	1.047 (1.017 − 1.077)
TNFRSF14_TCM_40h	Wald ratio	1	0.002	0.037	1.030 (1.011 − 1.050)
TNFRSF14_TN_40h	Wald ratio	1	0.001	0.019	1.040 (1.017 − 1.063)
NBL1_CD4_Memory_stim_40h	Wald ratio	1	0.001	0.030	1.026 (1.010 − 1.041)
NBL1_TCM_40h	Wald ratio	1	0.001	0.030	1.019 (1.007 − 1.030)
NBL1_TN_40h	Wald ratio	1	0.001	0.030	1.019 (1.007 − 1.030)
MED8_CD4_Memory_stim_40h	Wald ratio	1	0.002	0.045	0.916 (0.867 − 0.969)
MED8_CD4_Naive_stim_40h	Wald ratio	1	<0.001	0.018	0.972 (0.956 − 0.987)
FUBP1_CD4_Memory_stim_16h	Wald ratio	1	<0.001	0.005	0.970 (0.955 − 0.985)
FUBP1_CD4_Naive_stim_16h	Wald ratio	1	<0.001	0.005	0.960 (0.940 − 0.979)
FUBP1_TCM_16h	Wald ratio	1	<0.001	0.005	0.979 (0.968 − 0.989)
FUBP1_TN_16h	Wald ratio	1	<0.001	0.005	0.978 (0.967 − 0.989)
PBRM1_CD4_Memory_stim_40h	Wald ratio	1	<0.001	0.003	0.904 (0.861 − 0.948)
PBRM1_CD4_Naive_stim_16h	Wald ratio	1	<0.001	0.002	0.948 (0.926 − 0.971)
ANKS6_CD4_Naive_stim_40h	Wald ratio	1	0.002	0.038	1.031 (1.012 − 1.051)
ANKS6_TN_40h	Wald ratio	1	0.002	0.038	1.033 (1.012 − 1.053)
TRAPPC2L_CD4_Memory_stim_16h	Wald ratio	1	<0.001	0.004	1.051 (1.026 − 1.077)
TRAPPC2L_CD4_Memory_stim_40h	Wald ratio	1	<0.001	0.004	1.151 (1.076 − 1.231)
TRAPPC2L_CD4_Memory_stim_5d	Wald ratio	1	<0.001	0.004	1.049 (1.025 − 1.074)
TRAPPC2L_CD4_Memory_uns_0h	Wald ratio	1	<0.001	0.004	1.029 (1.015 − 1.043)
TRAPPC2L_CD4_Naive_stim_16h	Wald ratio	1	<0.001	0.004	1.059 (1.030 − 1.089)
TRAPPC2L_CD4_Naive_stim_40h	Wald ratio	1	<0.001	0.004	1.040 (1.021 − 1.060)
TRAPPC2L_CD4_Naive_stim_5d	Wald ratio	1	<0.001	0.004	1.044 (1.023 − 1.066)
TRAPPC2L_TCM_40h	Wald ratio	1	<0.001	0.004	1.034 (1.018 − 1.051)
TRAPPC2L_TN_0h	Wald ratio	1	<0.001	0.004	1.030 (1.015 − 1.044)
TRAPPC2L_TN_16h	Wald ratio	1	<0.001	0.004	1.029 (1.015 − 1.043)
TRAPPC2L_TN_40h	Wald ratio	1	<0.001	0.004	1.033 (1.017 − 1.050)
UBXN6_CD4_Memory_stim_16h	Wald ratio	1	<0.001	0.007	0.979 (0.969 − 0.990)
UBXN6_CD4_Memory_stim_40h	Wald ratio	1	0.001	0.034	0.967 (0.948 − 0.987)
UBXN6_CD4_Memory_stim_5d	Wald ratio	1	0.001	0.019	0.979 (0.967 − 0.991)
UBXN6_CD4_Naive_stim_16h	Wald ratio	1	0.001	0.034	0.980 (0.969 − 0.992)
UBXN6_CD4_Naive_stim_40h	Wald ratio	1	0.001	0.032	0.983 (0.972 − 0.993)
UBXN6_CD4_Naive_stim_5d	Wald ratio	1	0.001	0.034	0.982 (0.972 − 0.993)
UBXN6_CD4_Naive_uns_0h	Wald ratio	1	<0.001	0.007	0.976 (0.964 − 0.988)
UBXN6_TCM_16h	Wald ratio	1	<0.001	0.007	0.965 (0.948 − 0.983)
UBXN6_TCM_40h	Wald ratio	1	0.001	0.026	0.976 (0.962 − 0.990)
UBXN6_TEM_40h	Wald ratio	1	0.001	0.026	0.976 (0.962 − 0.990)
UBXN6_TN_0h	Wald ratio	1	<0.001	0.007	0.975 (0.963 − 0.988)
UBXN6_TN_16h	Wald ratio	1	0.001	0.034	0.979 (0.966 − 0.992)
UBXN6_TN_40h	Wald ratio	1	0.001	0.027	0.979 (0.966 − 0.991)
UBXN6_TN_IFN_5d	Wald ratio	1	0.001	0.032	0.981 (0.969 − 0.992)
NT5DC2_CD4_Memory_stim_16h	Wald ratio	1	<0.001	0.002	0.981 (0.972 − 0.989)
NT5DC2_CD4_Memory_stim_40h	Wald ratio	1	<0.001	0.002	0.980 (0.972 − 0.989)
NT5DC2_CD4_Memory_stim_5d	Wald ratio	1	<0.001	0.003	0.970 (0.956 − 0.984)
NT5DC2_CD4_Naive_stim_16h	Wald ratio	1	<0.001	0.003	0.979 (0.969 − 0.989)
NT5DC2_CD4_Naive_stim_40h	Wald ratio	1	<0.001	0.002	0.980 (0.972 − 0.989)
NT5DC2_CD4_Naive_stim_5d	Wald ratio	1	<0.001	0.003	0.971 (0.958 − 0.985)
NT5DC2_TCM_16h	Wald ratio	1	<0.001	0.002	0.981 (0.972 − 0.989)
NT5DC2_TCM_40h	Wald ratio	1	<0.001	0.002	0.982 (0.974 − 0.990)
NT5DC2_TCM_5d	Wald ratio	1	<0.001	0.002	0.966 (0.952 − 0.981)
NT5DC2_TEM_16h	Wald ratio	1	<0.001	0.002	0.973 (0.961 − 0.985)
NT5DC2_TEM_40h	Wald ratio	1	<0.001	0.002	0.981 (0.973 − 0.990)
NT5DC2_TEM_HLApositive_40h	Wald ratio	1	<0.001	0.002	0.980 (0.971 − 0.989)
NT5DC2_TM_ER−stress_40h	Wald ratio	1	<0.001	0.002	0.972 (0.959 − 0.984)
NT5DC2_TN_16h	Wald ratio	1	<0.001	0.003	0.982 (0.973 − 0.990)
NT5DC2_TN_40h	Wald ratio	1	<0.001	0.002	0.981 (0.973 − 0.989)
NT5DC2_TN_cycling_40h	Wald ratio	1	<0.001	0.002	0.980 (0.972 − 0.989)

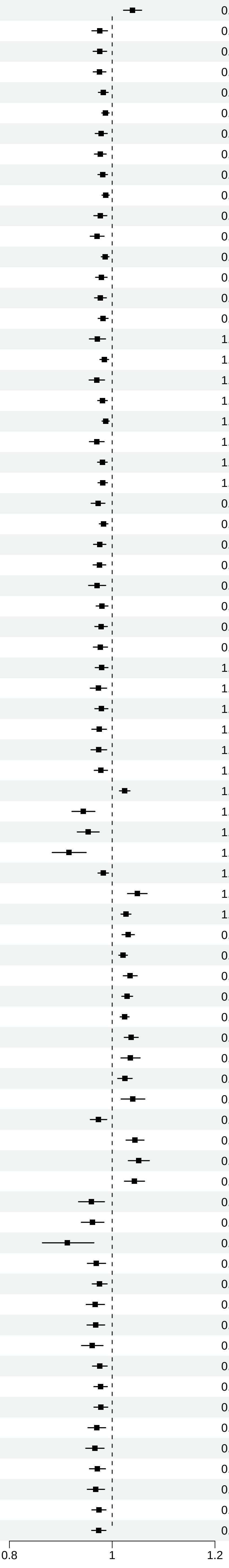

Supplement: Supplementary file 1 [file Supplementaryfile1.zip › Supplementary files/S3.pdf]
