# Supplementary material for: Dynamic genetic regulation of CD4+ T cells in obstructive sleep apnea: integrating context-specific eQTL, Mendelian randomization, single-cell sequencing, and experimental validation
Source: Front Immunol. 2025 Dec 17;16:1691347. doi: 10.3389/fimmu.2025.1691347 (PMC12753881; doi:10.3389/fimmu.2025.1691347)

Trait	Method	nSNP	pval	FDR	OR (95% CI)
NT5DC2_TN_IFN_40h	Wald ratio	1	<0.001	0.003	0.977 (0.966 − 0.988)
NT5DC2_TN2_40h	Wald ratio	1	<0.001	0.002	0.976 (0.965 − 0.987)
NDUFS5_CD4_Memory_stim_16h	Wald ratio	1	0.001	0.019	1.026 (1.011 − 1.041)
NDUFS5_CD4_Memory_stim_40h	Wald ratio	1	0.001	0.019	1.091 (1.039 − 1.145)
NDUFS5_CD4_Memory_stim_5d	Wald ratio	1	0.001	0.019	1.033 (1.014 − 1.053)
NDUFS5_CD4_Memory_uns_0h	Wald ratio	1	0.001	0.019	1.025 (1.011 − 1.040)
NDUFS5_CD4_Naive_stim_16h	Wald ratio	1	0.001	0.019	1.032 (1.014 − 1.050)
NDUFS5_CD4_Naive_stim_40h	Wald ratio	1	0.001	0.019	1.021 (1.009 − 1.033)
NDUFS5_CD4_Naive_stim_5d	Wald ratio	1	0.001	0.019	1.023 (1.010 − 1.036)
NDUFS5_CD4_Naive_uns_0h	Wald ratio	1	0.001	0.027	1.028 (1.011 − 1.045)
NDUFS5_nTreg_16h	Wald ratio	1	0.001	0.019	1.031 (1.014 − 1.049)
NDUFS5_nTreg_40h	Wald ratio	1	<0.001	0.002	1.031 (1.017 − 1.045)
NDUFS5_TCM_0h	Wald ratio	1	0.001	0.028	1.022 (1.009 − 1.035)
NDUFS5_TCM_16h	Wald ratio	1	0.001	0.019	1.020 (1.009 − 1.031)
NDUFS5_TCM_40h	Wald ratio	1	0.001	0.019	1.017 (1.007 − 1.027)
NDUFS5_TCM_5d	Wald ratio	1	0.001	0.019	1.037 (1.016 − 1.058)
NDUFS5_TEM_16h	Wald ratio	1	0.001	0.019	1.024 (1.010 − 1.038)
NDUFS5_TEM_40h	Wald ratio	1	0.001	0.019	1.019 (1.008 − 1.031)
NDUFS5_TEM_5d	Wald ratio	1	0.001	0.019	1.026 (1.011 − 1.040)
NDUFS5_TEM_HLApositive_40h	Wald ratio	1	0.001	0.027	1.091 (1.036 − 1.149)
NDUFS5_TN_0h	Wald ratio	1	0.001	0.027	1.027 (1.011 − 1.043)
NDUFS5_TN_16h	Wald ratio	1	0.001	0.019	1.022 (1.009 − 1.034)
NDUFS5_TN_40h	Wald ratio	1	0.001	0.019	1.018 (1.008 − 1.029)
NDUFS5_TN_5d	Wald ratio	1	0.001	0.019	1.022 (1.010 − 1.035)
NDUFS5_TN_cycling_40h	Wald ratio	1	0.001	0.019	1.020 (1.009 − 1.032)
NDUFS5_TN_cycling_5d	Wald ratio	1	0.001	0.019	1.025 (1.011 − 1.040)
NDUFS5_TN_HSP_5d	Wald ratio	1	0.001	0.019	1.020 (1.009 − 1.032)
NDUFS5_TN_IFN_40h	Wald ratio	1	0.001	0.019	1.021 (1.009 − 1.033)
NDUFS5_TN_IFN_5d	Wald ratio	1	0.001	0.027	1.023 (1.009 − 1.037)
NDUFS5_TN_NFKB	Wald ratio	1	0.001	0.027	1.022 (1.009 − 1.036)
SPNS1_CD4_Memory_stim_16h	Wald ratio	1	0.002	0.036	1.023 (1.008 − 1.037)
SPNS1_CD4_Memory_stim_40h	Wald ratio	1	0.002	0.036	1.032 (1.012 − 1.052)
SPNS1_CD4_Memory_stim_5d	Wald ratio	1	0.002	0.036	1.022 (1.008 − 1.036)
SPNS1_CD4_Naive_stim_16h	Wald ratio	1	0.002	0.036	1.025 (1.010 − 1.041)
SPNS1_CD4_Naive_stim_40h	Wald ratio	1	0.002	0.036	1.016 (1.006 − 1.026)
SPNS1_CD4_Naive_stim_5d	Wald ratio	1	0.002	0.036	1.018 (1.007 − 1.030)
SPNS1_TCM_16h	Wald ratio	1	0.001	0.032	1.025 (1.010 − 1.041)
SPNS1_TCM_40h	Wald ratio	1	0.001	0.032	1.025 (1.010 − 1.040)
SPNS1_TCM_5d	Wald ratio	1	0.002	0.036	1.029 (1.011 − 1.047)
SPNS1_TEM_5d	Wald ratio	1	0.001	0.032	1.028 (1.011 − 1.046)
SPNS1_TN_16h	Wald ratio	1	0.001	0.032	1.021 (1.008 − 1.035)
SPNS1_TN_40h	Wald ratio	1	0.002	0.036	1.016 (1.006 − 1.026)
SPNS1_TN_5d	Wald ratio	1	0.002	0.036	1.019 (1.007 − 1.030)
SPNS1_TN_cycling_40h	Wald ratio	1	<0.001	0.002	1.036 (1.020 − 1.052)
SPNS1_TN_IFN_5d	Wald ratio	1	0.002	0.036	1.032 (1.012 − 1.052)
TMEM126A_CD4_Memory_stim_16h	Wald ratio	1	<0.001	0.008	0.956 (0.934 − 0.979)
TMEM126A_CD4_Naive_stim_16h	Wald ratio	1	<0.001	0.004	0.956 (0.935 − 0.977)
TMEM126A_CD4_Naive_stim_40h	Wald ratio	1	<0.001	0.004	0.973 (0.960 − 0.986)
TMEM126A_TCM_16h	Wald ratio	1	<0.001	0.004	0.980 (0.971 − 0.990)
TMEM126A_TCM_40h	Wald ratio	1	<0.001	0.002	0.978 (0.968 − 0.987)
TMEM126A_TN_16h	Wald ratio	1	<0.001	0.004	0.982 (0.974 − 0.991)
TMEM126A_TN_40h	Wald ratio	1	<0.001	0.004	0.974 (0.961 − 0.986)
UQCRH_CD4_Memory_stim_16h	Wald ratio	1	<0.001	0.012	0.937 (0.905 − 0.970)
UQCRH_CD4_Memory_stim_40h	Wald ratio	1	<0.001	0.002	0.912 (0.875 − 0.951)
UQCRH_CD4_Memory_stim_5d	Wald ratio	1	<0.001	0.002	0.954 (0.934 − 0.975)
UQCRH_CD4_Memory_uns_0h	Wald ratio	1	<0.001	0.012	0.968 (0.952 − 0.985)
UQCRH_CD4_Naive_stim_16h	Wald ratio	1	<0.001	0.002	0.938 (0.911 − 0.966)
UQCRH_CD4_Naive_stim_40h	Wald ratio	1	<0.001	0.002	0.925 (0.893 − 0.958)
UQCRH_CD4_Naive_stim_5d	Wald ratio	1	<0.001	0.012	0.959 (0.939 − 0.981)
UQCRH_CD4_Naive_uns_0h	Wald ratio	1	<0.001	0.012	0.969 (0.953 − 0.985)
UQCRH_TCM_16h	Wald ratio	1	<0.001	0.002	0.965 (0.950 − 0.981)
UQCRH_TCM_40h	Wald ratio	1	<0.001	0.019	0.969 (0.951 − 0.986)
UQCRH_TCM_5d	Wald ratio	1	<0.001	0.002	0.955 (0.935 − 0.975)
UQCRH_TEM_40h	Wald ratio	1	<0.001	0.002	0.948 (0.926 − 0.971)
UQCRH_TN_0h	Wald ratio	1	<0.001	0.012	0.970 (0.954 − 0.986)
UQCRH_TN_16h	Wald ratio	1	<0.001	0.002	0.967 (0.953 − 0.982)
UQCRH_TN_40h	Wald ratio	1	<0.001	0.002	0.959 (0.941 − 0.977)
UQCRH_TN_5d	Wald ratio	1	<0.001	0.012	0.960 (0.940 − 0.981)
UQCRH_TN_cycling_40h	Wald ratio	1	<0.001	0.012	0.968 (0.952 − 0.985)
AKIRIN1_CD4_Memory_stim_40h	Wald ratio	1	<0.001	0.003	1.129 (1.067 − 1.195)
AKIRIN1_CD4_Naive_stim_16h	Wald ratio	1	<0.001	0.002	1.036 (1.019 − 1.052)
AKIRIN1_CD4_Naive_stim_40h	Wald ratio	1	<0.001	0.003	1.050 (1.026 − 1.074)
AKIRIN1_TCM_16h	Wald ratio	1	<0.001	0.002	1.035 (1.019 − 1.051)
AKIRIN1_TN_16h	Wald ratio	1	<0.001	0.002	1.028 (1.015 − 1.041)
AKIRIN1_TN_40h	Wald ratio	1	<0.001	0.003	1.045 (1.024 − 1.067)

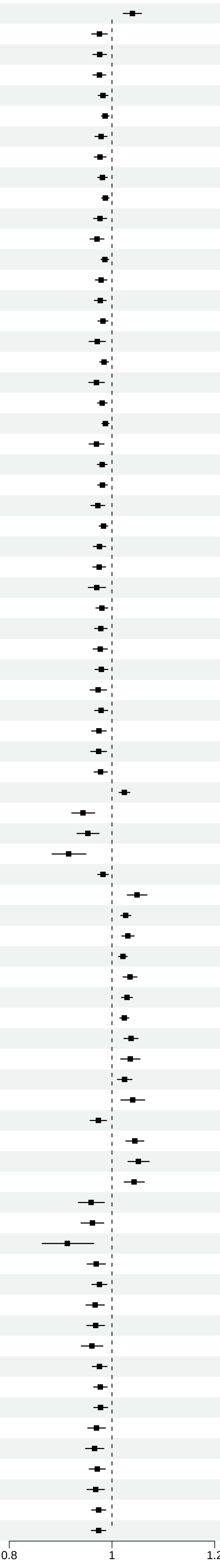

Supplement: Supplementary file 1 [file Supplementaryfile1.zip › Supplementary files/S4.pdf]
