# Supplementary material for: Dynamic genetic regulation of CD4+ T cells in obstructive sleep apnea: integrating context-specific eQTL, Mendelian randomization, single-cell sequencing, and experimental validation
Source: Front Immunol. 2025 Dec 17;16:1691347. doi: 10.3389/fimmu.2025.1691347 (PMC12753881; doi:10.3389/fimmu.2025.1691347)

Trait	Method	nSNP	pval	FDR	OR (95% CI)
SGF29_CD4_Memory_stim_5d	Wald ratio	1	<0.001	<0.001	0.965 (0.952 − 0.978)
SGF29_CD4_Memory_uns_0h	Wald ratio	1	<0.001	<0.001	0.973 (0.962 − 0.983)
SGF29_CD4_Naive_stim_5d	Wald ratio	1	<0.001	<0.001	0.971 (0.959 − 0.982)
SGF29_CD4_Naive_uns_0h	Wald ratio	1	<0.001	<0.001	0.971 (0.961 − 0.982)
SGF29_TCM_5d	Wald ratio	1	<0.001	<0.001	0.965 (0.951 − 0.978)
SGF29_TN_0h	Wald ratio	1	<0.001	<0.001	0.974 (0.964 − 0.984)
KCNA3_TCM_16h	Wald ratio	1	<0.001	0.015	0.969 (0.953 − 0.986)
PLEC_CD4_Memory_stim_16h	Wald ratio	1	<0.001	0.018	0.974 (0.960 − 0.988)
PLEC_CD4_Memory_stim_40h	Wald ratio	1	<0.001	0.018	0.974 (0.960 − 0.989)
PLEC_CD4_Memory_stim_5d	Wald ratio	1	<0.001	0.018	0.981 (0.970 − 0.991)
PLEC_CD4_Memory_uns_0h	Wald ratio	1	<0.001	0.018	0.985 (0.977 − 0.993)
PLEC_CD4_Naive_stim_16h	Wald ratio	1	<0.001	0.018	0.980 (0.969 − 0.991)
PLEC_CD4_Naive_stim_40h	Wald ratio	1	<0.001	0.018	0.981 (0.970 − 0.991)
PLEC_CD4_Naive_stim_5d	Wald ratio	1	<0.001	0.011	0.983 (0.974 − 0.992)
PLEC_CD4_Naive_uns_0h	Wald ratio	1	0.001	0.028	0.981 (0.970 − 0.992)
PLEC_TCM_0h	Wald ratio	1	<0.001	0.018	0.982 (0.973 − 0.992)
PLEC_TCM_40h	Wald ratio	1	<0.001	0.018	0.979 (0.967 − 0.991)
PLEC_TN_0h	Wald ratio	1	<0.001	0.011	0.980 (0.970 − 0.991)
PLEC_TN_40h	Wald ratio	1	<0.001	0.018	0.983 (0.973 − 0.992)
PLEC_TN_5d	Wald ratio	1	<0.001	0.018	0.979 (0.968 − 0.991)
PLEC_TN_IFN_40h	Wald ratio	1	<0.001	0.009	0.978 (0.967 − 0.990)
PLEC_TN_IFN_5d	Wald ratio	1	<0.001	0.018	0.977 (0.964 − 0.990)
ZFAND2A_TEM_40h	Wald ratio	1	0.001	0.026	1.018 (1.008 − 1.029)
EFCAB13_CD4_Naive_uns_0h	Wald ratio	1	0.001	0.028	0.973 (0.957 − 0.989)
EFCAB13_TN_0h	Wald ratio	1	0.001	0.028	0.972 (0.956 − 0.989)
TUFM_CD4_Memory_uns_0h	Wald ratio	1	<0.001	0.002	1.019 (1.011 − 1.027)
TUFM_CD4_Naive_uns_0h	Wald ratio	1	<0.001	0.002	1.020 (1.011 − 1.029)
TUFM_TCM_0h	Wald ratio	1	<0.001	0.002	1.020 (1.011 − 1.029)
TUFM_TEM_0h	Wald ratio	1	0.001	0.028	1.021 (1.008 − 1.033)
TUFM_TEM_5d	Wald ratio	1	0.001	0.028	1.049 (1.019 − 1.079)
TUFM_TN_0h	Wald ratio	1	<0.001	0.002	1.021 (1.012 − 1.030)
TOP1MT_CD4_Naive_stim_40h	Wald ratio	1	0.001	0.021	1.030 (1.013 − 1.047)
TOP1MT_TCM_40h	Wald ratio	1	0.001	0.022	1.027 (1.011 − 1.043)
TOP1MT_TN_40h	Wald ratio	1	<0.001	0.008	1.028 (1.014 − 1.043)
RBM43_CD4_Memory_uns_0h	Wald ratio	1	0.001	0.021	0.982 (0.972 − 0.992)
RBM43_CD4_Naive_uns_0h	Wald ratio	1	0.001	0.028	0.983 (0.973 − 0.993)
RBM43_TN_0h	Wald ratio	1	0.002	0.043	0.983 (0.972 − 0.994)
IMMP2L_CD4_Memory_stim_16h	Wald ratio	1	0.001	0.027	1.020 (1.008 − 1.033)
IMMP2L_CD4_Memory_stim_40h	Wald ratio	1	0.001	0.027	1.035 (1.014 − 1.057)
IMMP2L_CD4_Naive_stim_40h	Wald ratio	1	0.001	0.027	1.016 (1.006 − 1.026)
IMMP2L_TCM_16h	Wald ratio	1	0.001	0.027	1.023 (1.009 − 1.037)
IMMP2L_TN_40h	Wald ratio	1	0.001	0.027	1.018 (1.007 − 1.030)
POLR1D_CD4_Memory_stim_40h	Wald ratio	1	0.001	0.019	1.101 (1.042 − 1.162)
POLR1D_CD4_Memory_uns_0h	Wald ratio	1	0.001	0.019	1.025 (1.011 − 1.039)
POLR1D_CD4_Naive_stim_16h	Wald ratio	1	0.001	0.019	1.022 (1.009 − 1.035)
POLR1D_CD4_Naive_stim_40h	Wald ratio	1	0.001	0.019	1.028 (1.012 − 1.045)
POLR1D_CD4_Naive_stim_5d	Wald ratio	1	0.001	0.019	1.021 (1.009 − 1.033)
POLR1D_TCM_0h	Wald ratio	1	0.001	0.019	1.024 (1.010 − 1.038)
POLR1D_TN_0h	Wald ratio	1	0.001	0.019	1.024 (1.010 − 1.037)
POLR1D_TN_16h	Wald ratio	1	0.001	0.019	1.027 (1.012 − 1.042)
POLR1D_TN_40h	Wald ratio	1	0.001	0.019	1.025 (1.011 − 1.039)
POLR1D_TN_5d	Wald ratio	1	0.001	0.019	1.022 (1.010 − 1.035)
POLR1D_TN_cycling_40h	Wald ratio	1	0.001	0.019	1.030 (1.013 − 1.047)
POLR1D_TN_HSP_5d	Wald ratio	1	0.001	0.019	1.036 (1.015 − 1.057)
RPL14_CD4_Memory_uns_0h	Wald ratio	1	0.002	0.048	1.024 (1.009 − 1.040)
NMB_TN_40h	Wald ratio	1	0.001	0.031	0.980 (0.969 − 0.992)
STIMATE_CD4_Naive_stim_40h	Wald ratio	1	0.001	0.026	1.021 (1.009 − 1.034)
STIMATE_TN_16h	Wald ratio	1	0.001	0.026	1.018 (1.007 − 1.028)
KANSL1−AS1_CD4_Naive_stim_16h	Wald ratio	1	<0.001	0.002	1.019 (1.011 − 1.028)
KANSL1−AS1_TCM_40h	Wald ratio	1	<0.001	0.002	1.020 (1.011 − 1.029)
KANSL1−AS1_TCM_LA	Wald ratio	1	<0.001	0.006	1.027 (1.013 − 1.041)
KANSL1−AS1_TEM_40h	Wald ratio	1	<0.001	0.002	1.021 (1.012 − 1.031)
CCNT2−AS1_TCM_40h	Wald ratio	1	0.001	0.024	1.032 (1.013 − 1.051)
NPIPB2_CD4_Naive_uns_0h	Wald ratio	1	0.002	0.045	1.013 (1.005 − 1.021)
NPIPB2_TN_0h	Wald ratio	1	0.002	0.045	1.012 (1.004 − 1.020)
KIAA0040_T_ER−stress_5d	Wald ratio	1	0.002	0.048	1.022 (1.008 − 1.036)
KIAA0040_TN_16h	Wald ratio	1	0.001	0.027	1.025 (1.010 − 1.040)
KIAA0040_TN_40h	Wald ratio	1	0.002	0.048	1.016 (1.006 − 1.027)
KIAA0040_TN_HSP_5d	Wald ratio	1	0.002	0.048	1.017 (1.006 − 1.029)
NCK1−DT_CD4_Memory_stim_40h	Wald ratio	1	<0.001	0.018	1.023 (1.010 − 1.035)
NCK1−DT_CD4_Naive_stim_40h	Wald ratio	1	<0.001	0.002	1.026 (1.014 − 1.037)
NCK1−DT_TCM_40h	Wald ratio	1	<0.001	0.002	1.035 (1.019 − 1.052)
NCK1−DT_TN_40h	Wald ratio	1	<0.001	0.002	1.022 (1.012 − 1.032)
FAM13A−AS1_CD4_Naive_uns_0h	Wald ratio	1	<0.001	0.002	0.972 (0.960 − 0.984)
FAM13A−AS1_TN_0h	Wald ratio	1	<0.001	0.002	0.972 (0.960 − 0.984)

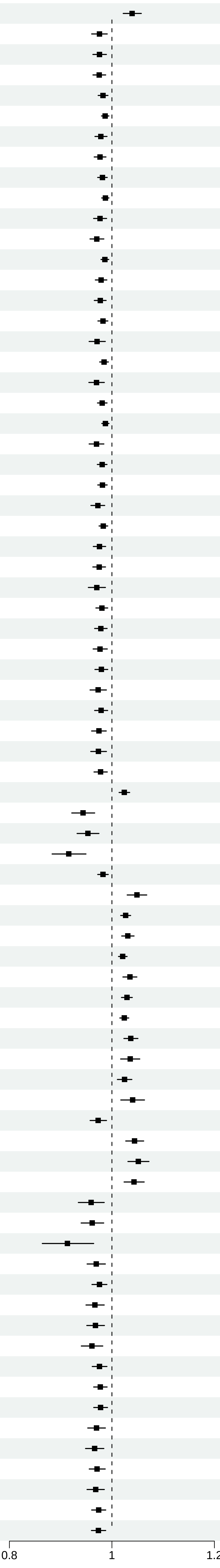

Supplement: Supplementary file 1 [file Supplementaryfile1.zip › Supplementary files/S5.pdf]
