# Supplementary material for: Dynamic genetic regulation of CD4+ T cells in obstructive sleep apnea: integrating context-specific eQTL, Mendelian randomization, single-cell sequencing, and experimental validation
Source: Front Immunol. 2025 Dec 17;16:1691347. doi: 10.3389/fimmu.2025.1691347 (PMC12753881; doi:10.3389/fimmu.2025.1691347)

Trait	Method	nSNP	pval	FDR	OR (95% CI)
AL118516.1_CD4_Memory_stim_40h	Wald ratio	1	0.002	0.036	1.022 (1.008 − 1.036)
AL118516.1_CD4_Memory_stim_5d	Wald ratio	1	0.002	0.036	1.023 (1.009 − 1.037)
AL118516.1_TN_cycling_40h	Wald ratio	1	0.002	0.036	1.044 (1.017 − 1.073)
AC008731.1_CD4_Memory_uns_0h	Wald ratio	1	0.001	0.019	0.979 (0.968 − 0.991)
AC008731.1_TN_0h	Wald ratio	1	0.001	0.019	0.977 (0.965 − 0.990)
AC004148.1_CD4_Memory_stim_16h	Wald ratio	1	0.001	0.027	1.018 (1.007 − 1.029)
AC004148.1_CD4_Memory_stim_40h	Wald ratio	1	0.001	0.027	1.019 (1.008 − 1.031)
AC004148.1_CD4_Memory_stim_5d	Wald ratio	1	0.001	0.027	1.014 (1.006 − 1.023)
AC004148.1_CD4_Memory_uns_0h	Wald ratio	1	<0.001	0.007	1.022 (1.011 − 1.034)
AC004148.1_CD4_Naive_stim_16h	Wald ratio	1	0.001	0.027	1.018 (1.007 − 1.029)
AC004148.1_CD4_Naive_stim_40h	Wald ratio	1	0.001	0.027	1.015 (1.006 − 1.023)
AC004148.1_CD4_Naive_stim_5d	Wald ratio	1	0.001	0.027	1.014 (1.005 − 1.022)
AC004148.1_CD4_Naive_uns_0h	Wald ratio	1	0.001	0.027	1.016 (1.006 − 1.026)
AC004148.1_HSP_16h	Wald ratio	1	0.001	0.027	1.025 (1.010 − 1.041)
AC004148.1_nTreg_16h	Wald ratio	1	0.001	0.027	1.027 (1.011 − 1.043)
AC004148.1_nTreg_40h	Wald ratio	1	0.001	0.027	1.020 (1.008 − 1.032)
AC004148.1_T_ER−stress_5d	Wald ratio	1	0.001	0.027	1.021 (1.008 − 1.034)
AC004148.1_TCM_0h	Wald ratio	1	<0.001	0.007	1.024 (1.012 − 1.036)
AC004148.1_TCM_16h	Wald ratio	1	0.001	0.027	1.013 (1.005 − 1.021)
AC004148.1_TCM_40h	Wald ratio	1	0.001	0.027	1.014 (1.005 − 1.022)
AC004148.1_TCM_5d	Wald ratio	1	0.001	0.027	1.015 (1.006 − 1.025)
AC004148.1_TEM_0h	Wald ratio	1	0.001	0.027	1.021 (1.009 − 1.035)
AC004148.1_TEM_16h	Wald ratio	1	0.001	0.027	1.023 (1.009 − 1.037)
AC004148.1_TEM_40h	Wald ratio	1	0.001	0.027	1.016 (1.006 − 1.026)
AC004148.1_TEM_5d	Wald ratio	1	0.001	0.027	1.018 (1.007 − 1.029)
AC004148.1_TEM_HLApositive_40h	Wald ratio	1	0.001	0.027	1.023 (1.009 − 1.036)
AC004148.1_TM_ER−stress_40h	Wald ratio	1	0.001	0.027	1.020 (1.008 − 1.032)
AC004148.1_TN_0h	Wald ratio	1	0.001	0.027	1.018 (1.007 − 1.028)
AC004148.1_TN_16h	Wald ratio	1	0.001	0.027	1.014 (1.006 − 1.023)
AC004148.1_TN_40h	Wald ratio	1	0.001	0.027	1.013 (1.005 − 1.021)
AC004148.1_TN_5d	Wald ratio	1	0.001	0.027	1.015 (1.006 − 1.025)
AC004148.1_TN_cycling_40h	Wald ratio	1	0.001	0.027	1.014 (1.006 − 1.023)
AC004148.1_TN_cycling_5d	Wald ratio	1	0.001	0.027	1.016 (1.007 − 1.026)
AC004148.1_TN_HSP_5d	Wald ratio	1	0.001	0.027	1.015 (1.006 − 1.024)
AC004148.1_TN_IFN_40h	Wald ratio	1	0.001	0.027	1.022 (1.009 − 1.035)
AC004148.1_TN_IFN_5d	Wald ratio	1	0.001	0.027	1.018 (1.007 − 1.029)
AC004148.1_TN_NFKB	Wald ratio	1	0.001	0.027	1.021 (1.008 − 1.033)
LINC02210−CRHR1_CD4_Naive_uns_0h	Wald ratio	1	<0.001	0.002	1.025 (1.014 − 1.037)
LINC02210−CRHR1_TN_0h	Wald ratio	1	<0.001	0.002	1.029 (1.016 − 1.043)
ASB16−AS1_CD4_Memory_stim_40h	Wald ratio	1	<0.001	0.001	0.961 (0.945 − 0.977)
ASB16−AS1_CD4_Memory_stim_5d	Wald ratio	1	<0.001	0.002	0.974 (0.963 − 0.985)
ASB16−AS1_CD4_Naive_stim_16h	Wald ratio	1	<0.001	0.004	0.970 (0.956 − 0.985)
ASB16−AS1_CD4_Naive_stim_40h	Wald ratio	1	<0.001	0.007	0.981 (0.971 − 0.990)
ASB16−AS1_CD4_Naive_stim_5d	Wald ratio	1	<0.001	0.004	0.980 (0.970 − 0.990)
ASB16−AS1_TCM_40h	Wald ratio	1	<0.001	0.001	0.967 (0.954 − 0.981)
ASB16−AS1_TEM_5d	Wald ratio	1	<0.001	0.004	0.982 (0.973 − 0.990)
ASB16−AS1_TN_16h	Wald ratio	1	<0.001	0.001	0.972 (0.961 − 0.984)
ASB16−AS1_TN_40h	Wald ratio	1	<0.001	0.002	0.977 (0.967 − 0.987)
ASB16−AS1_TN_5d	Wald ratio	1	<0.001	0.007	0.969 (0.953 − 0.984)
ZNHIT3_CD4_Memory_stim_40h	Wald ratio	1	<0.001	0.015	1.086 (1.038 − 1.136)
ZNHIT3_CD4_Memory_stim_5d	Wald ratio	1	0.001	0.032	1.024 (1.009 − 1.038)
ZNHIT3_CD4_Naive_stim_16h	Wald ratio	1	<0.001	0.015	1.018 (1.008 − 1.027)
ZNHIT3_CD4_Naive_stim_40h	Wald ratio	1	<0.001	0.015	1.015 (1.007 − 1.023)
ZNHIT3_CD4_Naive_stim_5d	Wald ratio	1	0.001	0.032	1.017 (1.007 − 1.028)
ZNHIT3_TCM_16h	Wald ratio	1	<0.001	0.015	1.019 (1.009 − 1.030)
ZNHIT3_TCM_40h	Wald ratio	1	<0.001	0.015	1.027 (1.012 − 1.042)
ZNHIT3_TCM_5d	Wald ratio	1	<0.001	0.015	1.026 (1.012 − 1.040)
ZNHIT3_TN_16h	Wald ratio	1	<0.001	0.015	1.015 (1.007 − 1.024)
ZNHIT3_TN_40h	Wald ratio	1	<0.001	0.015	1.015 (1.007 − 1.023)
ZNHIT3_TN_cycling_40h	Wald ratio	1	<0.001	0.015	1.023 (1.010 − 1.035)
ZNHIT3_TN_IFN_40h	Wald ratio	1	<0.001	0.015	1.022 (1.010 − 1.034)
ZNHIT3_TN_IFN_5d	Wald ratio	1	<0.001	0.015	1.023 (1.010 − 1.035)

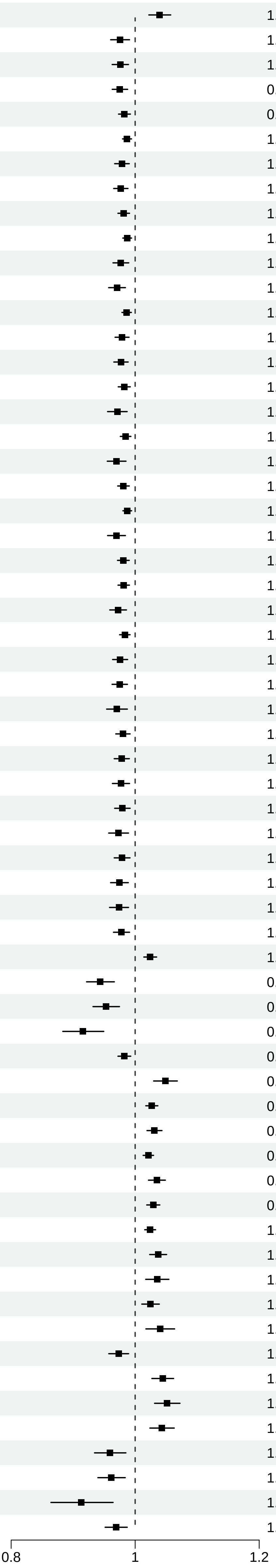

Supplement: Supplementary file 1 [file Supplementaryfile1.zip › Supplementary files/S6.pdf]
