# Supplementary material for: Dynamic genetic regulation of CD4+ T cells in obstructive sleep apnea: integrating context-specific eQTL, Mendelian randomization, single-cell sequencing, and experimental validation
Source: Front Immunol. 2025 Dec 17;16:1691347. doi: 10.3389/fimmu.2025.1691347 (PMC12753881; doi:10.3389/fimmu.2025.1691347)

Trait	Method	nSNP	*P*−Value	**OR (95% CI)**	FDR
A−575C2.4	Wald ratio	1	0.001	1.088 (1.035 − 1.144)	0.044
AC007401.2	Wald ratio	1	0.001	0.963 (0.942 − 0.985)	0.045
AC016725.4	Wald ratio	1	<0.001	1.136 (1.065 − 1.212)	0.011
AC018720.10	Wald ratio	1	<0.001	1.045 (1.029 − 1.060)	<0.001
AC068610.3	Wald ratio	1	<0.001	1.259 (1.142 − 1.387)	0.002
ACSS2	Wald ratio	1	<0.001	1.303 (1.124 − 1.509)	0.029
ACTBP14	Wald ratio	1	<0.001	1.063 (1.027 − 1.100)	0.031
AL049757.3	Wald ratio	1	<0.001	0.774 (0.692 − 0.866)	0.002
ANKRD42	Wald ratio	1	<0.001	1.114 (1.051 − 1.180)	0.022
ANKS6	Wald ratio	1	0.001	1.147 (1.058 − 1.242)	0.043
AP006216.11	Wald ratio	1	<0.001	1.160 (1.073 − 1.254)	0.017
ARID4B	Wald ratio	1	0.001	0.831 (0.744 − 0.928)	0.048
ATP2A1	Wald ratio	1	<0.001	1.266 (1.145 − 1.399)	0.002
ATXN7	Wald ratio	1	<0.001	1.452 (1.211 − 1.741)	0.007
BAIAP2L1	Wald ratio	1	0.001	0.897 (0.843 − 0.954)	0.034
C15orf27	Wald ratio	1	<0.001	1.554 (1.303 − 1.854)	0.001
C17orf53	Wald ratio	1	<0.001	0.858 (0.799 − 0.922)	0.005
C19orf24	Wald ratio	1	0.001	0.903 (0.852 − 0.957)	0.035
C1orf54	Wald ratio	1	<0.001	0.851 (0.783 − 0.924)	0.013
C1QBP	Wald ratio	1	0.001	0.768 (0.658 − 0.897)	0.044
C2orf47	Wald ratio	1	<0.001	0.681 (0.594 − 0.782)	<0.001
C3orf38	Wald ratio	1	0.001	1.130 (1.052 − 1.215)	0.044
C9orf169	Wald ratio	1	0.001	1.052 (1.021 − 1.083)	0.041
CACYBP	Wald ratio	1	<0.001	0.695 (0.596 − 0.811)	0.002
CADM2	Wald ratio	1	<0.001	1.629 (1.395 − 1.902)	<0.001
CCDC17	Wald ratio	1	<0.001	0.853 (0.795 − 0.916)	0.003
CDC20	Wald ratio	1	0.001	0.804 (0.711 − 0.910)	0.033
CIRBP	Wald ratio	1	0.001	0.798 (0.700 − 0.910)	0.041
CLP1	Wald ratio	1	0.001	0.866 (0.796 − 0.942)	0.043
CORO6	Wald ratio	1	<0.001	1.154 (1.083 − 1.230)	0.003
CPEB3	Wald ratio	1	0.001	0.852 (0.774 − 0.938)	0.049
CRAMP1L	Wald ratio	1	<0.001	1.088 (1.045 − 1.133)	0.006
CREB3L4	Wald ratio	1	0.001	0.912 (0.864 − 0.962)	0.041
CREBZF	Wald ratio	1	<0.001	1.432 (1.175 − 1.744)	0.027
CTD−2574D22.2	Wald ratio	1	0.001	1.103 (1.040 − 1.169)	0.048
CTD−3065J16.6	Wald ratio	1	<0.001	0.938 (0.910 − 0.966)	0.004
DTX2P1	Wald ratio	1	<0.001	1.091 (1.046 − 1.137)	0.006
DUSP11	Wald ratio	1	<0.001	1.306 (1.126 − 1.515)	0.029
DYNLRB1	Wald ratio	1	<0.001	1.436 (1.209 − 1.705)	0.005
EFCAB5	Wald ratio	1	<0.001	0.884 (0.836 − 0.935)	0.003
ELF3	Wald ratio	1	<0.001	0.859 (0.812 − 0.908)	<0.001
ERBB2IP	Wald ratio	1	<0.001	0.689 (0.577 − 0.823)	0.005
ETV5	Wald ratio	1	<0.001	0.824 (0.784 − 0.867)	<0.001
EVI5	Wald ratio	1	<0.001	1.096 (1.042 − 1.153)	0.027
FASTK	Wald ratio	1	<0.001	1.246 (1.115 − 1.393)	0.011
FBXO46	Wald ratio	1	<0.001	0.753 (0.657 − 0.864)	0.006
FILIP1L	Wald ratio	1	0.001	1.363 (1.143 − 1.624)	0.034
GAPVD1	Wald ratio	1	<0.001	0.745 (0.653 − 0.849)	0.003
GBE1	Wald ratio	1	0.001	0.904 (0.852 − 0.959)	0.043
GIN1	Wald ratio	1	<0.001	1.130 (1.066 − 1.197)	0.005
GNL3	Wald ratio	1	<0.001	0.867 (0.814 − 0.925)	0.003
HDGFRP2	Wald ratio	1	<0.001	1.259 (1.136 − 1.395)	0.003
HMGCR	Wald ratio	1	<0.001	0.863 (0.802 − 0.929)	0.010
HN1L	Wald ratio	1	<0.001	0.880 (0.834 − 0.928)	0.001
IGHV4−55	Wald ratio	1	<0.001	1.107 (1.052 − 1.165)	0.011
IL20RB	Wald ratio	1	<0.001	1.465 (1.227 − 1.749)	0.004
IPPK	Wald ratio	1	0.001	1.201 (1.077 − 1.338)	0.045
ITIH3	Wald ratio	1	0.001	1.156 (1.065 − 1.254)	0.033
KBTBD7	Wald ratio	1	0.001	0.919 (0.876 − 0.964)	0.035
LINC00342	Wald ratio	1	0.001	0.960 (0.937 − 0.983)	0.040
LOXL4	Wald ratio	1	<0.001	0.752 (0.644 − 0.878)	0.024
LSM5	Wald ratio	1	0.001	1.270 (1.106 − 1.458)	0.040
MAK16	Wald ratio	1	<0.001	0.912 (0.871 − 0.956)	0.011
MAPKAP1	Wald ratio	1	0.001	0.864 (0.792 − 0.943)	0.048
MAST2	Wald ratio	1	<0.001	0.859 (0.802 − 0.920)	0.003

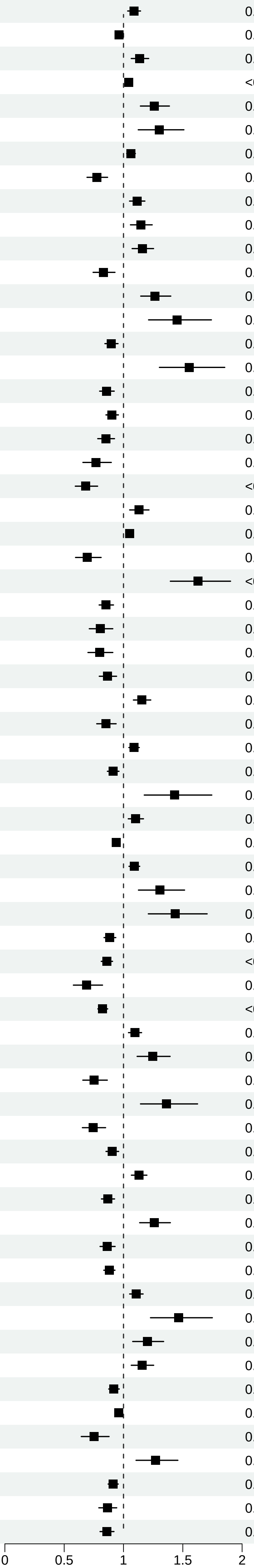

Supplement: Supplementary file 1 [file Supplementaryfile1.zip › Supplementary files/S7.pdf]
