# Supplementary material for: Dynamic genetic regulation of CD4+ T cells in obstructive sleep apnea: integrating context-specific eQTL, Mendelian randomization, single-cell sequencing, and experimental validation
Source: Front Immunol. 2025 Dec 17;16:1691347. doi: 10.3389/fimmu.2025.1691347 (PMC12753881; doi:10.3389/fimmu.2025.1691347)

Trait	Method	nSNP	*P*−Value	OR (95% CI)	FDR
MITF	Wald ratio	1	<0.001	1.268 (1.124 − 1.431)	0.011
MOV10	Wald ratio	1	<0.001	0.915 (0.874 − 0.957)	0.013
MPL	Wald ratio	1	0.001	0.919 (0.875 − 0.964)	0.033
MRM1	Wald ratio	1	0.001	1.245 (1.094 − 1.416)	0.044
MSL3P1	Wald ratio	1	0.001	1.110 (1.046 − 1.178)	0.035
MSTO1	Wald ratio	1	<0.001	1.218 (1.115 − 1.331)	0.003
MYO19	Wald ratio	1	<0.001	1.113 (1.050 − 1.180)	0.024
NEK4	Wald ratio	1	<0.001	0.743 (0.645 − 0.855)	0.005
NKAPL	Wald ratio	1	<0.001	1.420 (1.219 − 1.653)	0.002
NUDT19	Wald ratio	1	0.001	1.213 (1.081 − 1.361)	0.048
NUP160	Wald ratio	1	<0.001	0.860 (0.801 − 0.923)	0.005
PAQR5	Wald ratio	1	<0.001	1.294 (1.136 − 1.474)	0.011
PARPBP	Wald ratio	1	0.001	0.802 (0.703 − 0.915)	0.048
PIGW	Wald ratio	1	<0.001	0.833 (0.756 − 0.917)	0.017
PPP2R3A	Wald ratio	1	<0.001	1.196 (1.098 − 1.302)	0.005
PRPS1P2	Wald ratio	1	<0.001	1.081 (1.042 − 1.121)	0.004
PTPRJ	Wald ratio	1	<0.001	1.091 (1.044 − 1.140)	0.011
PYCRL	Wald ratio	1	0.001	1.341 (1.132 − 1.590)	0.040
QPCTL	Wald ratio	1	<0.001	0.792 (0.716 − 0.876)	0.002
RALY	Wald ratio	1	<0.001	1.483 (1.230 − 1.788)	0.005
RBM34	Wald ratio	1	<0.001	1.322 (1.146 − 1.524)	0.012
RDH11	Wald ratio	1	0.001	1.204 (1.078 − 1.345)	0.048
REST	Wald ratio	1	0.001	0.769 (0.657 − 0.900)	0.049
RP1−130H16.16	Wald ratio	1	<0.001	0.908 (0.863 − 0.956)	0.020
RP1−265C24.5	Wald ratio	1	<0.001	0.944 (0.918 − 0.969)	0.004
RP1−265C24.9	Wald ratio	1	<0.001	0.888 (0.838 − 0.940)	0.007
RP1−92O14.3	Wald ratio	1	0.001	0.943 (0.912 − 0.975)	0.035
RP1−92O14.6	Wald ratio	1	0.001	0.925 (0.884 − 0.967)	0.035
RP11−1348G14.4	Wald ratio	1	<0.001	0.935 (0.909 − 0.963)	0.002
RP11−219B17.1	Wald ratio	1	<0.001	0.928 (0.893 − 0.963)	0.011
RP11−318C24.2	Wald ratio	1	<0.001	0.965 (0.946 − 0.984)	0.023
RP11−395I6.3	Wald ratio	1	<0.001	1.103 (1.070 − 1.138)	<0.001
RP11−50I19.1	Wald ratio	1	<0.001	1.111 (1.066 − 1.157)	<0.001
RP11−574K11.5	Wald ratio	1	<0.001	0.875 (0.813 − 0.943)	0.029
RP11−767N6.7	Wald ratio	1	<0.001	0.855 (0.796 − 0.918)	0.004
RP11−893F2.14	Wald ratio	1	0.001	1.082 (1.035 − 1.131)	0.032
RP13−46H24.1	Wald ratio	1	0.001	0.951 (0.924 − 0.980)	0.043
RP4−756G23.5	Wald ratio	1	<0.001	1.066 (1.031 − 1.104)	0.019
RP5−1014C4.3	Wald ratio	1	0.001	1.050 (1.020 − 1.081)	0.049
RPAIN	Wald ratio	1	0.001	0.889 (0.829 − 0.953)	0.044
SH2B1	Wald ratio	1	<0.001	0.697 (0.598 − 0.812)	0.002
SHISA5	Wald ratio	1	<0.001	1.313 (1.129 − 1.527)	0.028
SIGLEC19P	Wald ratio	1	<0.001	0.891 (0.842 − 0.943)	0.009
SLC35G2	Wald ratio	1	<0.001	0.739 (0.645 − 0.848)	0.003
SNF8	Wald ratio	1	<0.001	1.222 (1.094 − 1.366)	0.028
SNORA66	Wald ratio	1	<0.001	0.778 (0.694 − 0.872)	0.003
SORBS1	Wald ratio	1	<0.001	1.279 (1.121 − 1.459)	0.020
SORT1	Wald ratio	1	<0.001	0.791 (0.710 − 0.880)	0.003
SPATC1	Wald ratio	1	<0.001	0.897 (0.852 − 0.944)	0.005
SRSF6	Wald ratio	1	0.001	0.829 (0.743 − 0.925)	0.042
STARD7	Wald ratio	1	0.001	0.766 (0.655 − 0.897)	0.045
SWI5	Wald ratio	1	<0.001	1.418 (1.195 − 1.684)	0.008
TIMM17A	Wald ratio	1	<0.001	0.784 (0.687 − 0.896)	0.025
TMEM106B	Wald ratio	1	<0.001	1.151 (1.064 − 1.246)	0.030
TMUB2	Wald ratio	1	<0.001	0.836 (0.764 − 0.914)	0.010
TMX2	Wald ratio	1	0.001	1.049 (1.020 − 1.078)	0.044
UBXN7	Wald ratio	1	<0.001	0.788 (0.711 − 0.874)	0.002
URB2	Wald ratio	1	<0.001	1.289 (1.132 − 1.467)	0.012
VPS52	Wald ratio	1	<0.001	0.842 (0.779 − 0.911)	0.004
WBSCR16	Wald ratio	1	0.001	0.820 (0.730 − 0.922)	0.044
WDR46	Wald ratio	1	<0.001	1.275 (1.145 − 1.420)	0.003
WNT3	Wald ratio	1	<0.001	1.175 (1.095 − 1.262)	0.002
ZCCHC8	Wald ratio	1	<0.001	1.231 (1.099 − 1.379)	0.024
ZNF311	Wald ratio	1	<0.001	1.266 (1.156 − 1.387)	<0.001
ZNF865	Wald ratio	1	<0.001	0.750 (0.639 − 0.881)	0.030

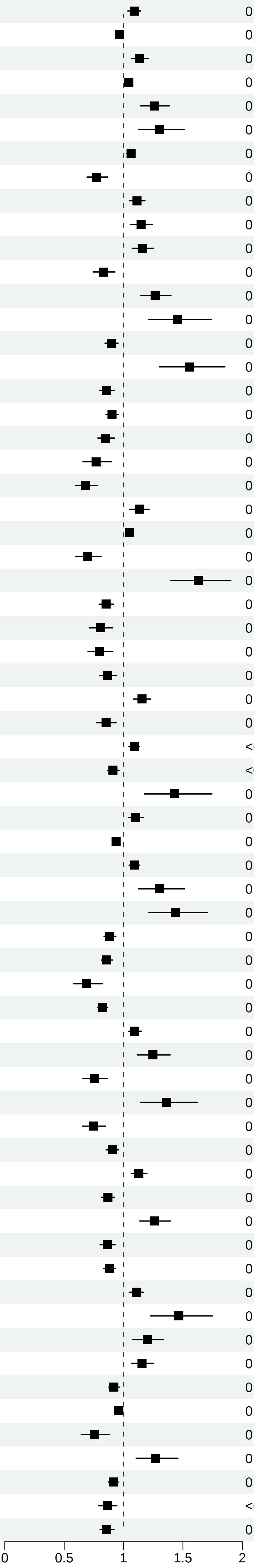

Supplement: Supplementary file 1 [file Supplementaryfile1.zip › Supplementary files/S8.pdf]
