# Supplementary material for: Dynamic genetic regulation of CD4+ T cells in obstructive sleep apnea: integrating context-specific eQTL, Mendelian randomization, single-cell sequencing, and experimental validation
Source: Front Immunol. 2025 Dec 17;16:1691347. doi: 10.3389/fimmu.2025.1691347 (PMC12753881; doi:10.3389/fimmu.2025.1691347)

Trait	Method	nSNP	*P*−Value	**OR (95% CI)**	FDR
ZUFSP	Wald ratio	1	0.001	0.900 (0.846 − 0.958)	0.044
A4GALT	IVW	8	0.001	1.039 (1.016 − 1.063)	0.048
AC010883.5	IVW	2	<0.001	1.072 (1.048 − 1.098)	<0.001
AC011893.3	IVW	5	<0.001	0.977 (0.965 − 0.990)	0.031
AC011899.9	IVW	4	0.001	1.026 (1.010 − 1.041)	0.048
AC132872.1	IVW	2	<0.001	1.092 (1.041 − 1.145)	0.022
ACOT1	IVW	7	<0.001	1.070 (1.034 − 1.108)	0.011
ACTN1	IVW	2	<0.001	1.169 (1.090 − 1.253)	0.003
AMPD3	IVW	6	<0.001	1.070 (1.035 − 1.106)	0.008
ANKRD36B	IVW	5	<0.001	1.023 (1.011 − 1.036)	0.020
ANKRD36C	IVW	2	<0.001	0.852 (0.795 − 0.914)	0.002
ARFGAP3	IVW	3	<0.001	0.923 (0.882 − 0.965)	0.031
ARID4A	IVW	2	0.001	1.119 (1.047 − 1.196)	0.045
ASB16	IVW	3	<0.001	0.839 (0.774 − 0.910)	0.004
ASB16−AS1	IVW	14	0.001	0.956 (0.932 − 0.981)	0.038
ATP9B	IVW	10	<0.001	1.067 (1.033 − 1.102)	0.011
BRI3	IVW	4	<0.001	0.941 (0.914 − 0.968)	0.005
C12orf39	IVW	5	<0.001	1.040 (1.017 − 1.063)	0.030
C15orf40	IVW	6	<0.001	1.081 (1.040 − 1.123)	0.010
C18orf8	IVW	4	<0.001	0.927 (0.898 − 0.957)	0.001
C1orf145	IVW	2	<0.001	1.051 (1.025 − 1.077)	0.009
C3orf18	IVW	2	<0.001	0.829 (0.761 − 0.902)	0.003
C4orf32	IVW	11	<0.001	1.053 (1.028 − 1.079)	0.005
C4orf33	IVW	5	<0.001	0.954 (0.934 − 0.975)	0.004
C5orf51	IVW	2	<0.001	0.864 (0.798 − 0.937)	0.027
CA8	IVW	5	0.001	0.940 (0.907 − 0.975)	0.043
CALCRL	IVW	4	<0.001	1.115 (1.057 − 1.175)	0.007
CAPNS1	IVW	5	<0.001	1.093 (1.045 − 1.142)	0.011
CAST	IVW	15	0.001	0.974 (0.960 − 0.989)	0.045
CBFA2T3	IVW	2	<0.001	0.865 (0.815 − 0.919)	0.001
CBLN3	IVW	17	<0.001	0.966 (0.947 − 0.984)	0.024
CCDC101	IVW	18	<0.001	0.959 (0.942 − 0.977)	0.002
CCDC157	IVW	3	<0.001	0.864 (0.809 − 0.922)	0.003
CCDC77	IVW	12	0.001	1.061 (1.026 − 1.097)	0.034
CCNT2	IVW	2	<0.001	0.944 (0.915 − 0.974)	0.022
CLEC16A	IVW	2	<0.001	0.884 (0.830 − 0.943)	0.015
CLIC6	IVW	2	<0.001	0.895 (0.850 − 0.944)	0.005
CLN3	IVW	5	<0.001	0.901 (0.866 − 0.938)	<0.001
CNOT2	IVW	2	0.001	0.839 (0.757 − 0.929)	0.043
CRHR1−IT1	IVW	19	<0.001	1.026 (1.014 − 1.038)	0.002
CRYBG3	IVW	2	<0.001	1.080 (1.042 − 1.120)	0.005
CSNK1D	IVW	3	<0.001	1.045 (1.019 − 1.071)	0.030
CTB−50L17.9	IVW	3	0.001	0.980 (0.968 − 0.992)	0.049
CTC−479C5.10	IVW	2	<0.001	1.133 (1.069 − 1.201)	0.005
CTD−2260A17.1	IVW	5	<0.001	1.028 (1.015 − 1.040)	0.003
CTSK	IVW	14	0.001	0.971 (0.954 − 0.988)	0.048
CXCR6	IVW	7	<0.001	1.079 (1.043 − 1.115)	0.003
DDX20	IVW	3	0.001	1.125 (1.051 − 1.205)	0.039
DDX55	IVW	11	<0.001	1.042 (1.020 − 1.064)	0.016
DERA	IVW	2	0.001	1.160 (1.062 − 1.268)	0.048
DND1P1	IVW	5	<0.001	1.021 (1.012 − 1.030)	0.002
DOC2GP	IVW	13	0.001	1.021 (1.009 − 1.034)	0.044
DOCK3	IVW	2	<0.001	0.901 (0.853 − 0.953)	0.019
DPP8	IVW	2	<0.001	1.189 (1.116 − 1.266)	<0.001
EFCAB1	IVW	2	<0.001	1.148 (1.069 − 1.232)	0.013
EFCAB4A	IVW	15	<0.001	1.039 (1.018 − 1.061)	0.021
EPM2AIP1	IVW	4	<0.001	0.900 (0.856 − 0.947)	0.006
FAHD2B	IVW	3	0.001	0.960 (0.938 − 0.983)	0.036
FAM103A1	IVW	2	<0.001	0.917 (0.874 − 0.961)	0.023
FAM135A	IVW	3	<0.001	0.927 (0.890 − 0.967)	0.026
FAM13A−AS1	IVW	3	<0.001	0.935 (0.903 − 0.969)	0.017
FAM157B	IVW	2	0.001	0.956 (0.931 − 0.982)	0.044
FAM172A	IVW	2	<0.001	0.880 (0.824 − 0.940)	0.014
FAM185BP	IVW	2	0.001	1.047 (1.020 − 1.075)	0.036
FAM63B	IVW	4	<0.001	0.943 (0.914 − 0.972)	0.014

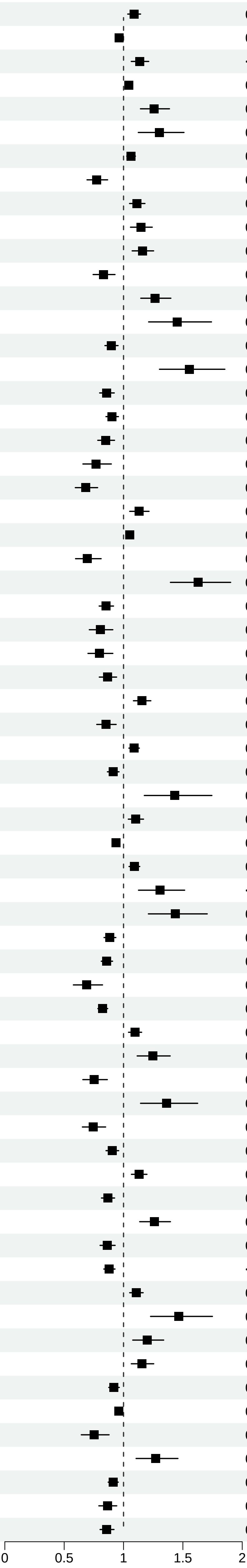

Supplement: Supplementary file 1 [file Supplementaryfile1.zip › Supplementary files/S9.pdf]
