# Supplementary figures and images for: Dynamic genetic regulation of CD4+ T cells in obstructive sleep apnea: integrating context-specific eQTL, Mendelian randomization, single-cell sequencing, and experimental validation
Source: Front Immunol. 2025 Dec 17;16:1691347. doi: 10.3389/fimmu.2025.1691347 (PMC12753881; doi:10.3389/fimmu.2025.1691347)

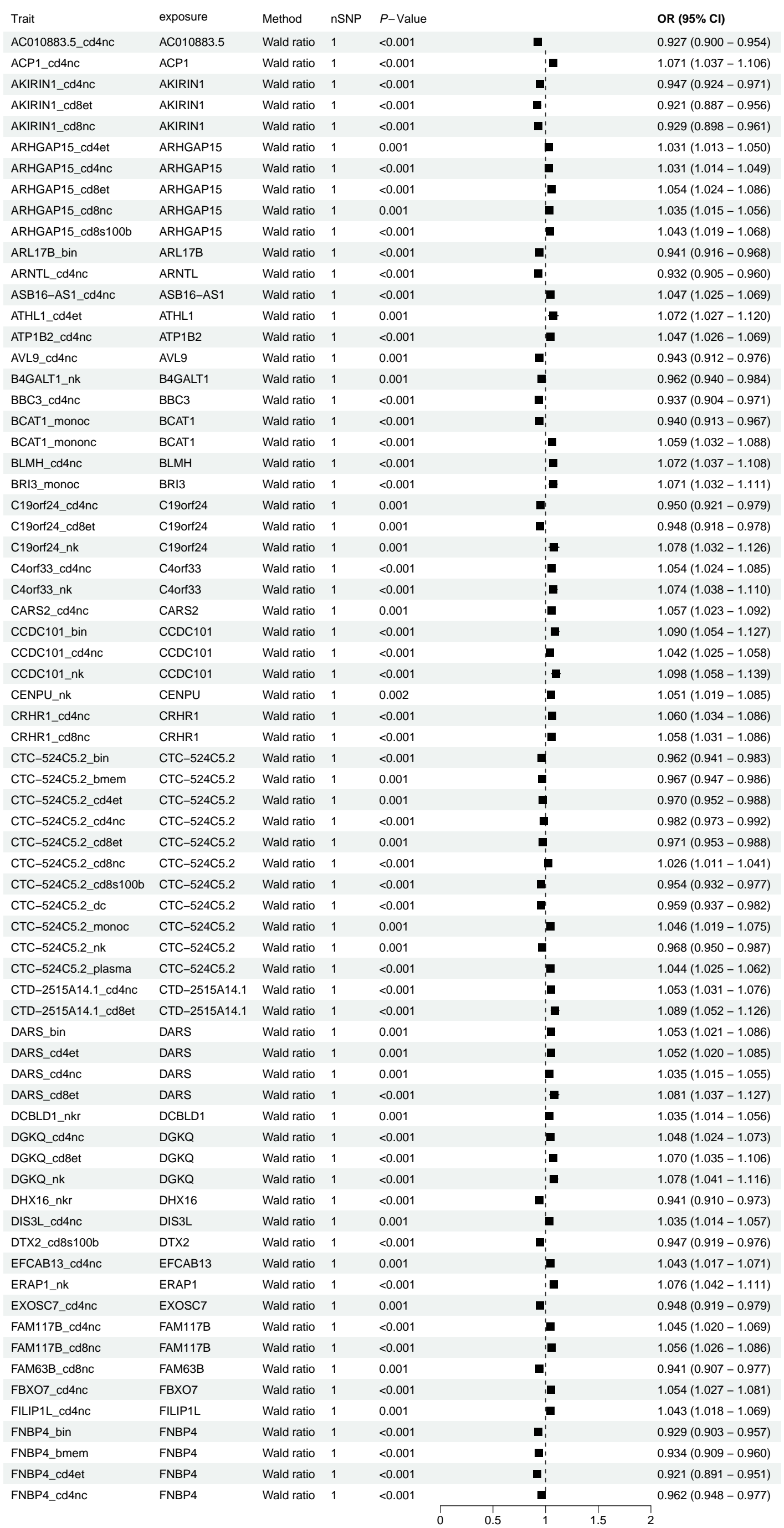

Supplement: Supplementary file 1 [file Supplementaryfile1.zip › Supplementary files/S12.pdf]

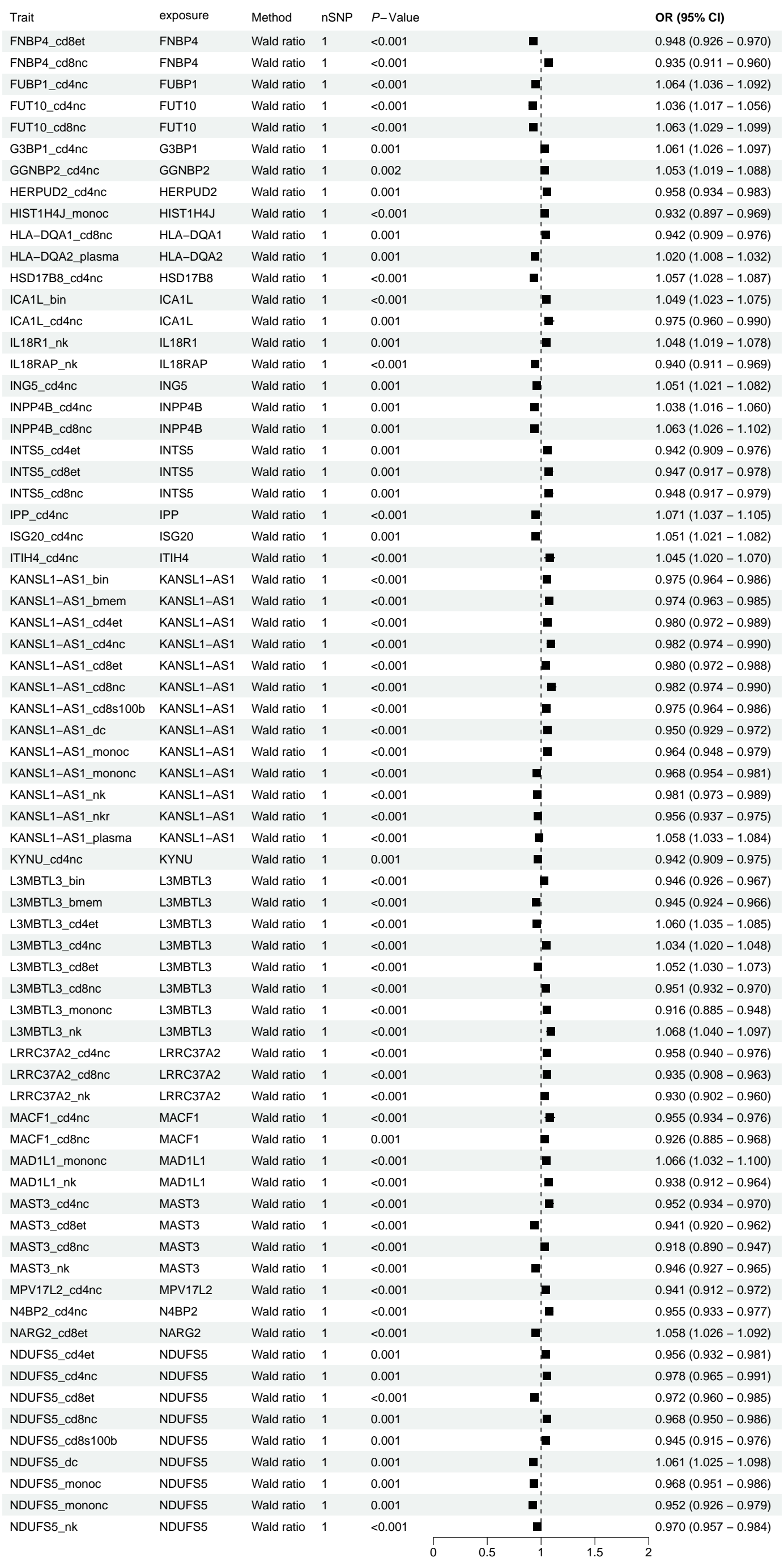

Supplement: Supplementary file 1 [file Supplementaryfile1.zip › Supplementary files/S13.pdf]

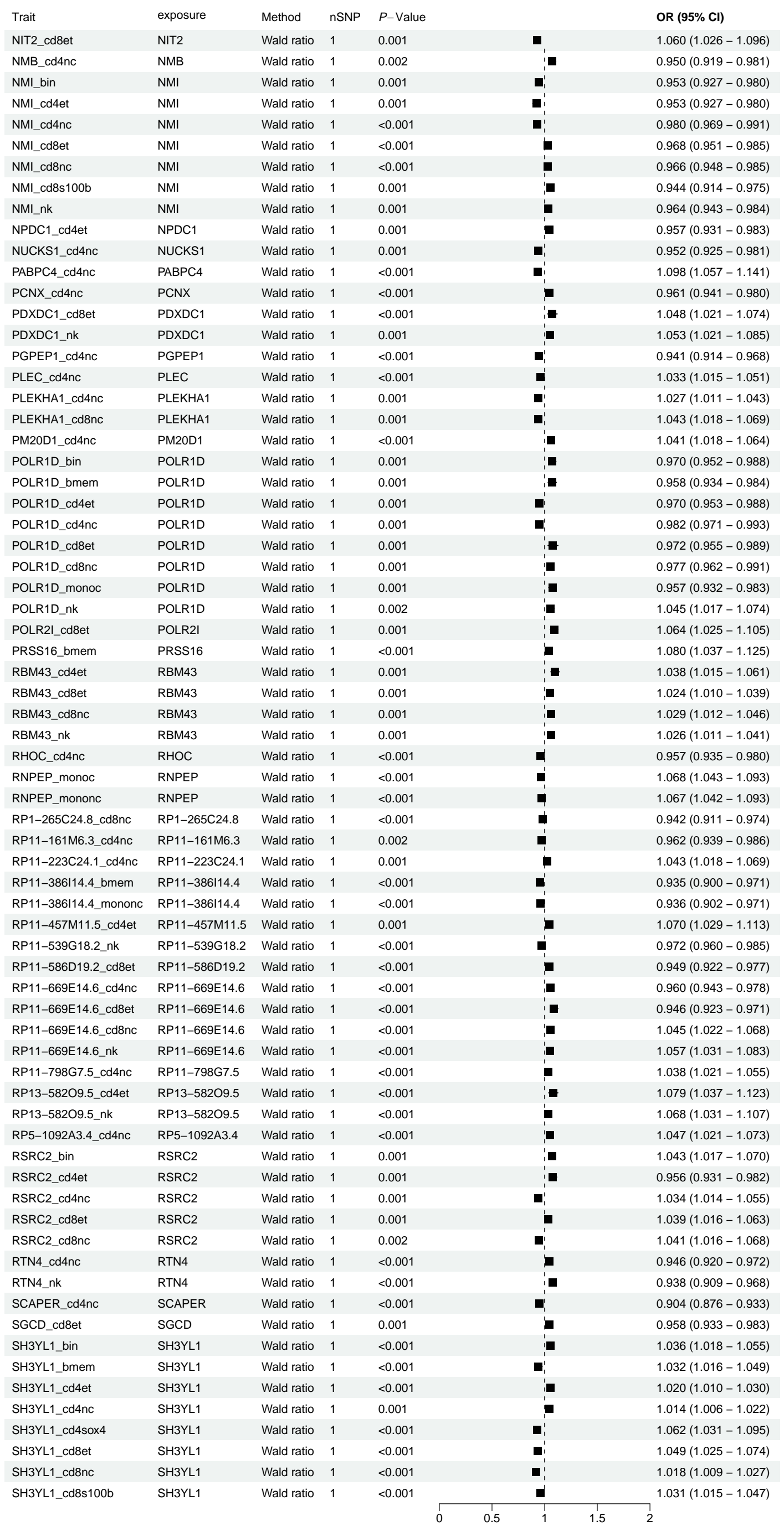

Supplement: Supplementary file 1 [file Supplementaryfile1.zip › Supplementary files/S14.pdf]

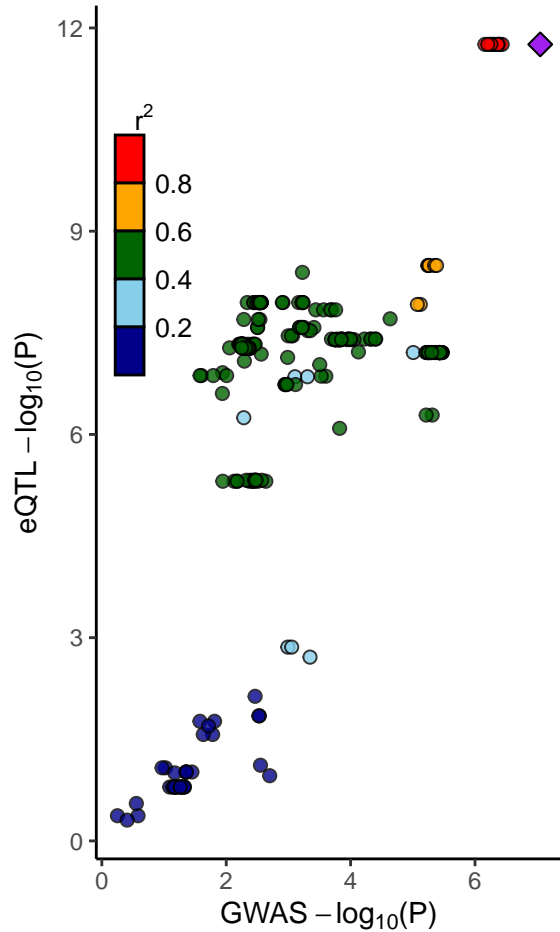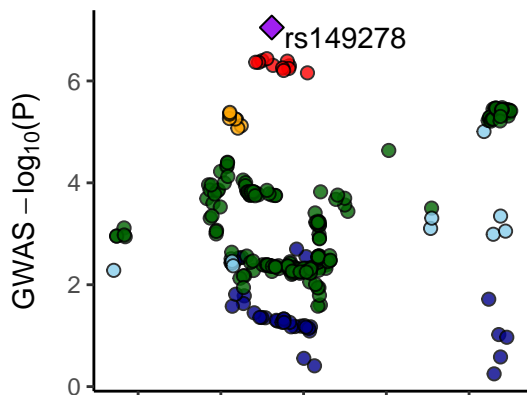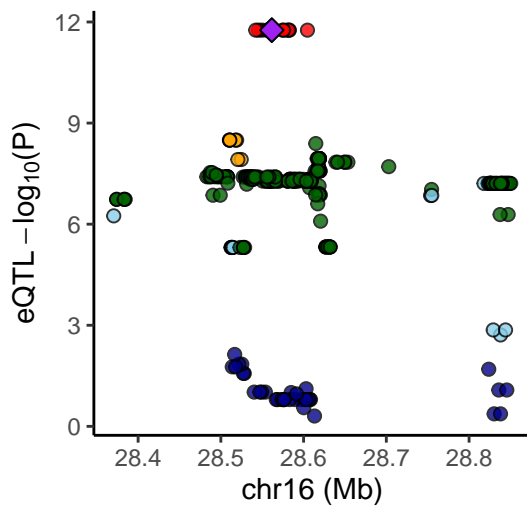

Supplement: Supplementary file 1 [file Supplementaryfile1.zip › Supplementary files/Supplementary Material 13/CD4_Memory_stim_5d_500kb_combined_ENSG00000176476.pdf]

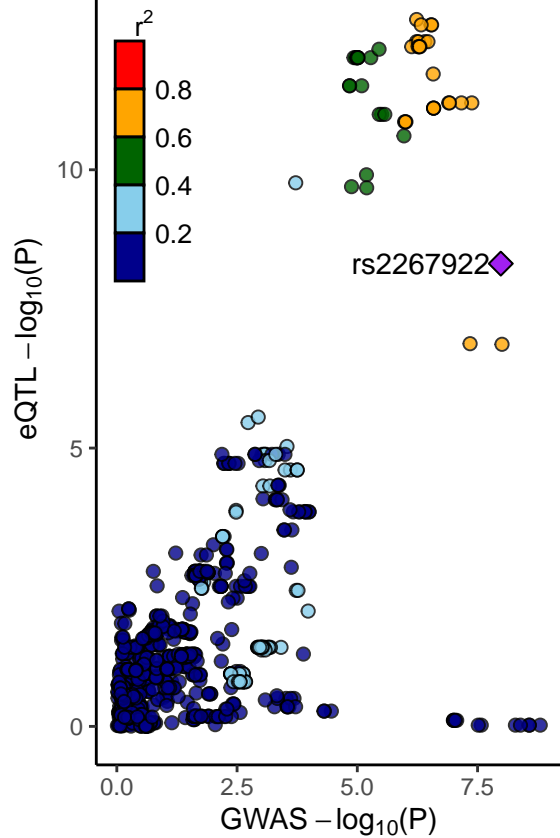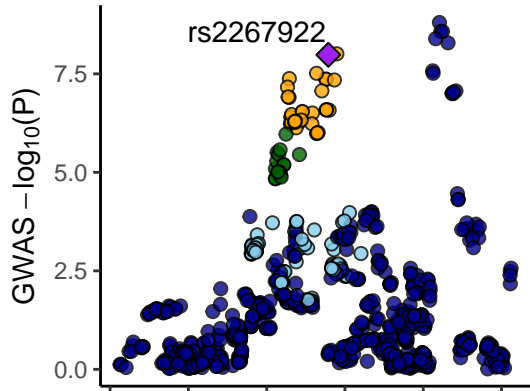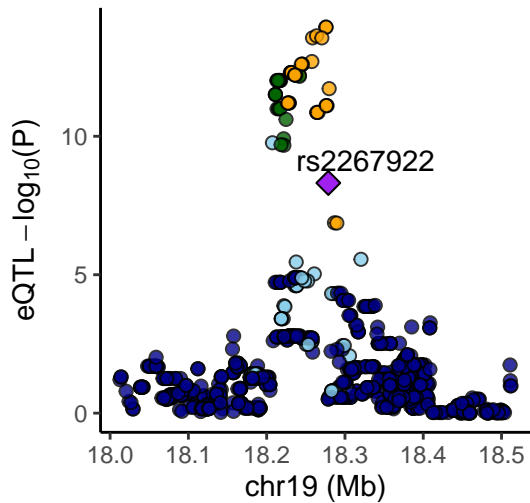

Supplement: Supplementary file 1 [file Supplementaryfile1.zip › Supplementary files/Supplementary Material 13/CD4_Memory_uns_0h_500kb_combined_ENSG00000099308.pdf]

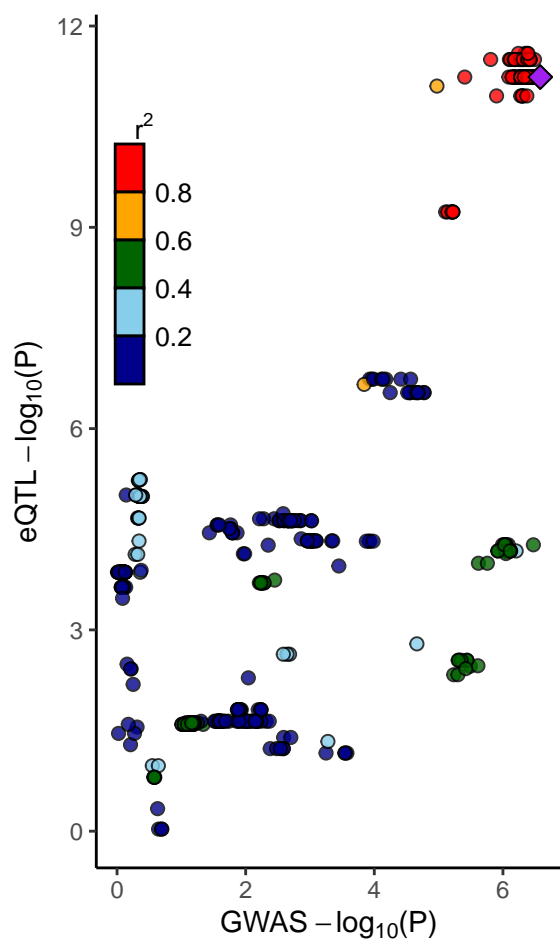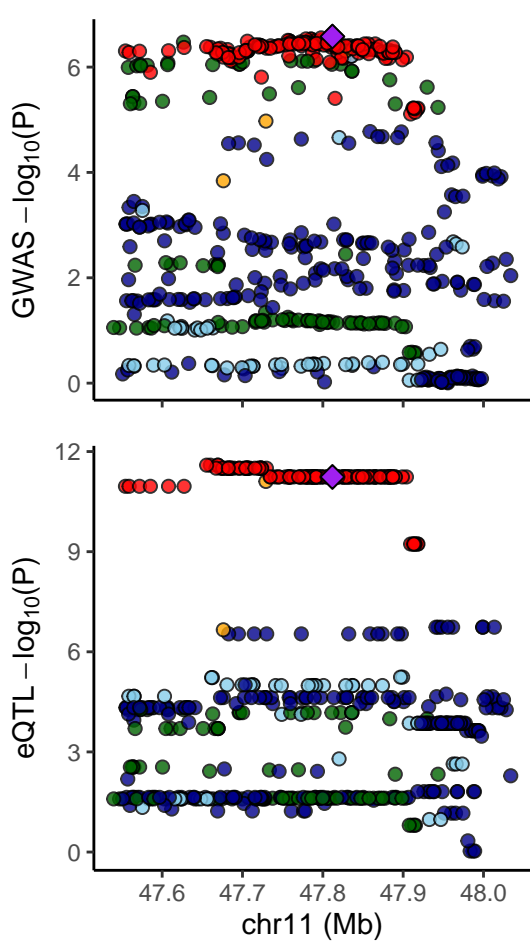

Supplement: Supplementary file 1 [file Supplementaryfile1.zip › Supplementary files/Supplementary Material 13/CD4_Memory_uns_0h_500kb_combined_ENSG00000109920.pdf]

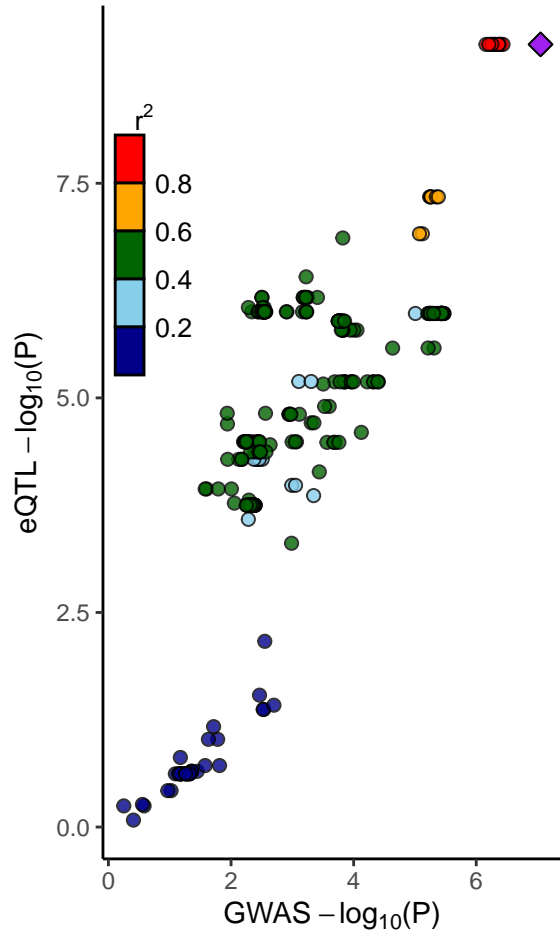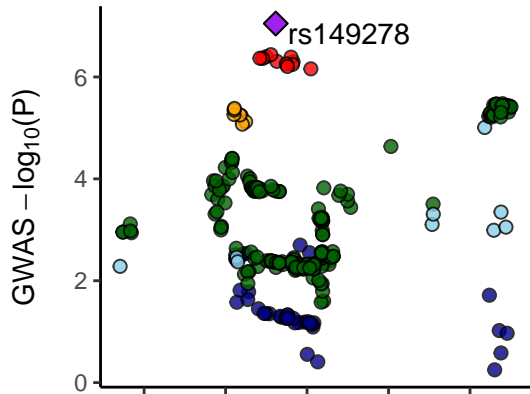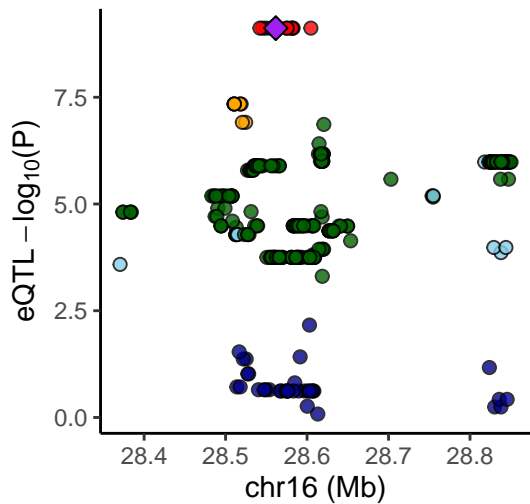

Supplement: Supplementary file 1 [file Supplementaryfile1.zip › Supplementary files/Supplementary Material 13/CD4_Memory_uns_0h_500kb_combined_ENSG00000176476.pdf]

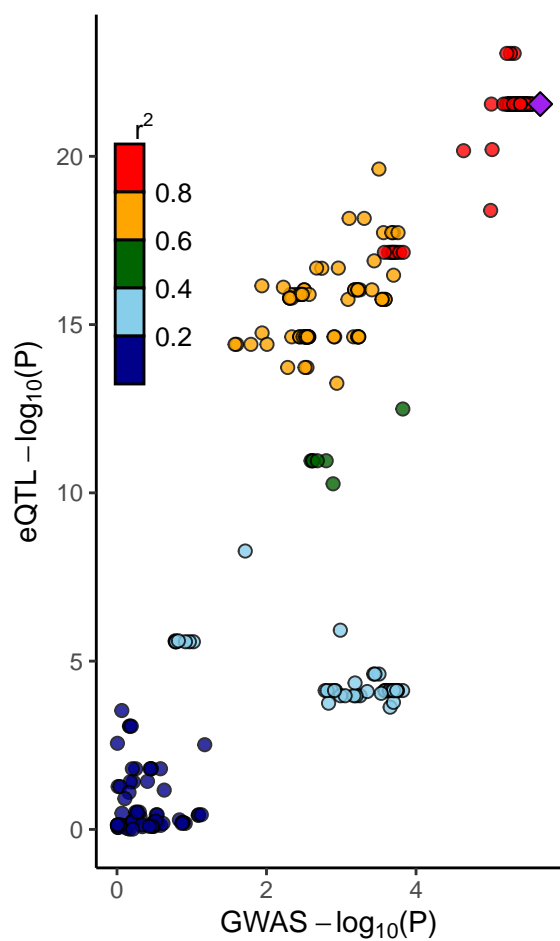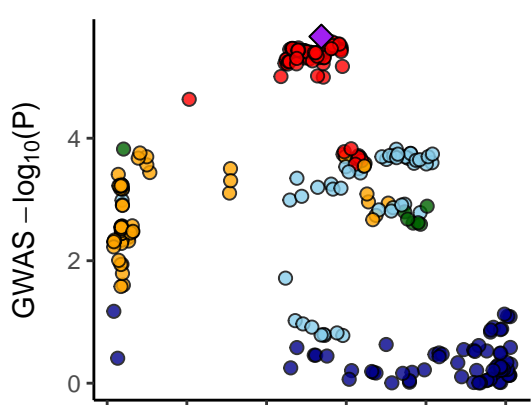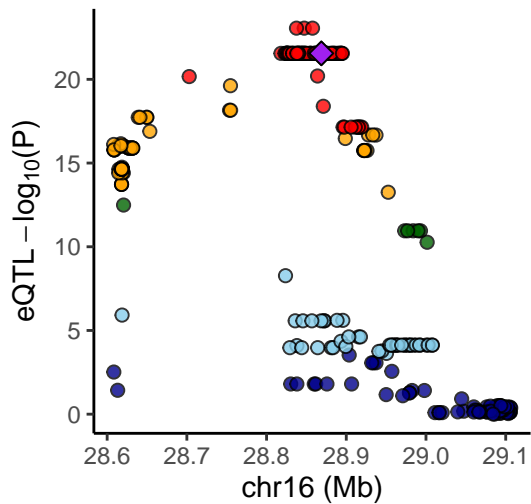

Supplement: Supplementary file 1 [file Supplementaryfile1.zip › Supplementary files/Supplementary Material 13/CD4_Memory_uns_0h_500kb_combined_ENSG00000178952.pdf]

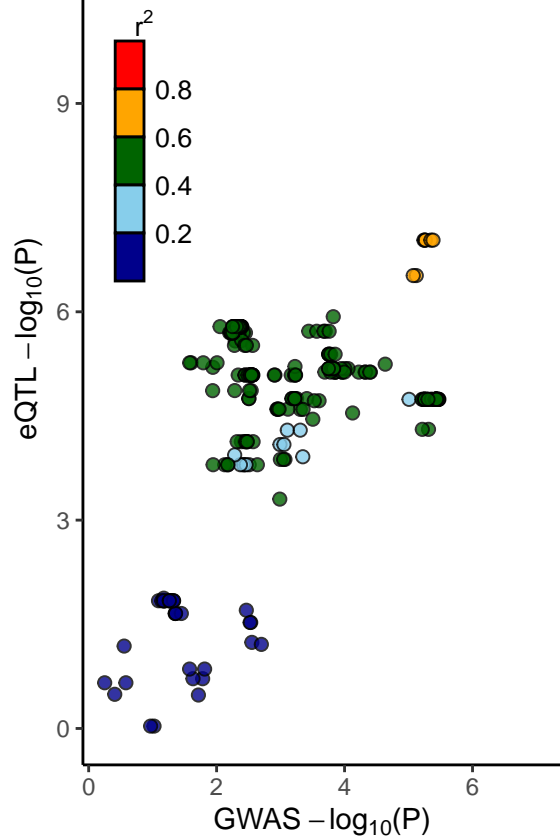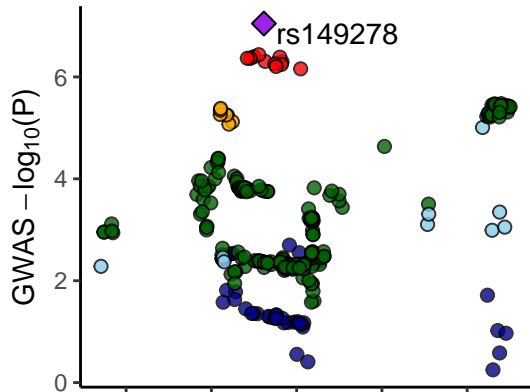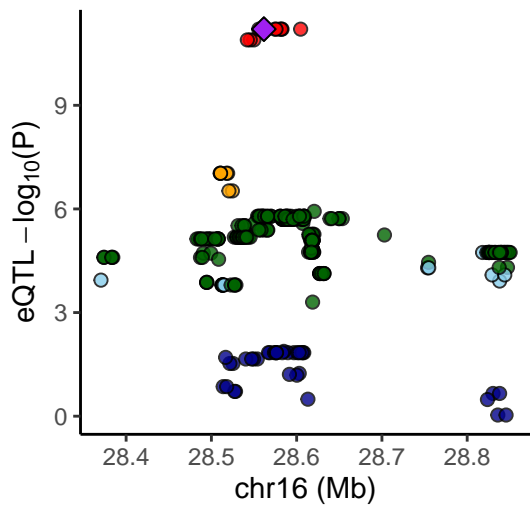

Supplement: Supplementary file 1 [file Supplementaryfile1.zip › Supplementary files/Supplementary Material 13/CD4_Naive_stim_5d_500kb_combined_ENSG00000176476.pdf]

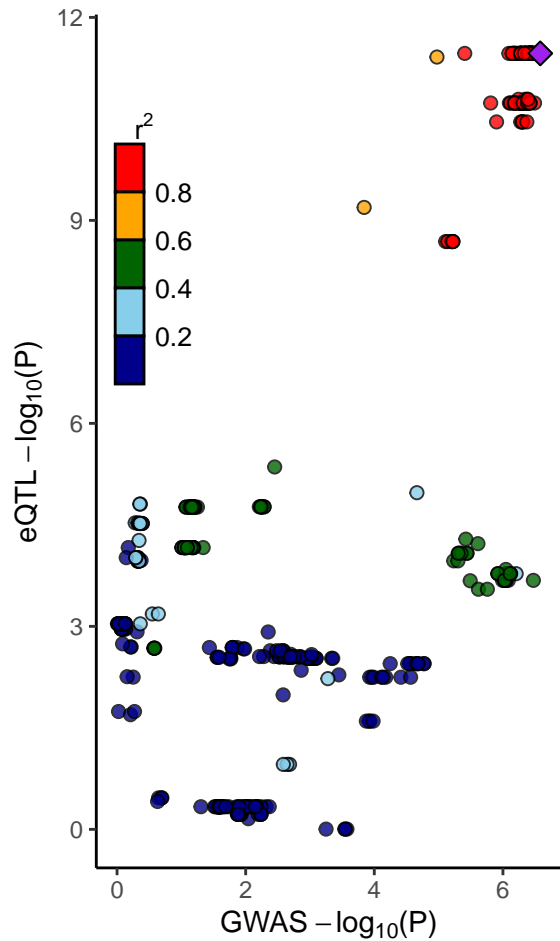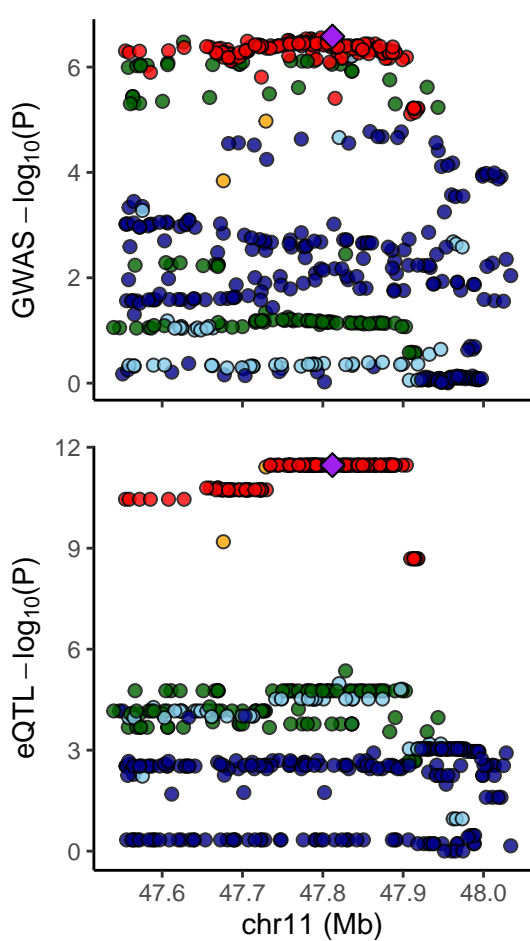

Supplement: Supplementary file 1 [file Supplementaryfile1.zip › Supplementary files/Supplementary Material 13/CD4_Naive_uns_0h_500kb_combined_ENSG00000109920.pdf]

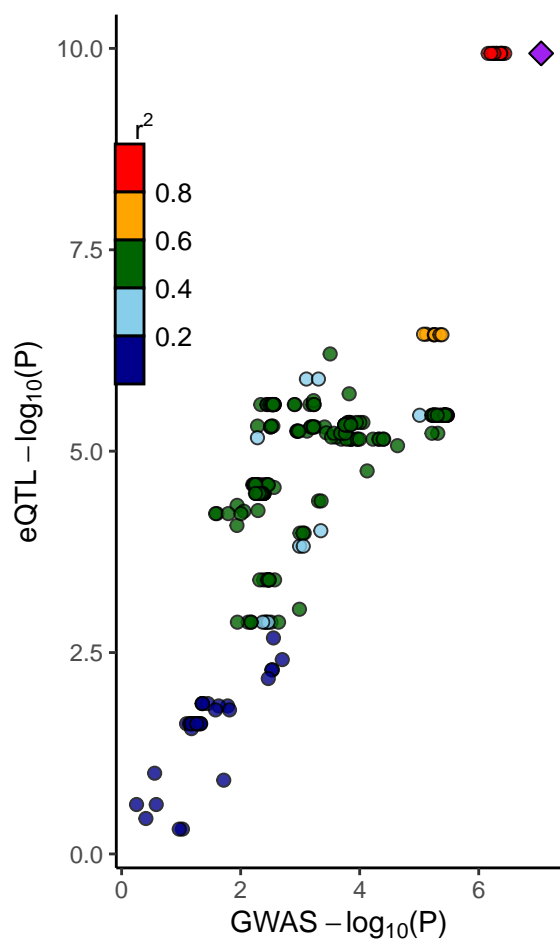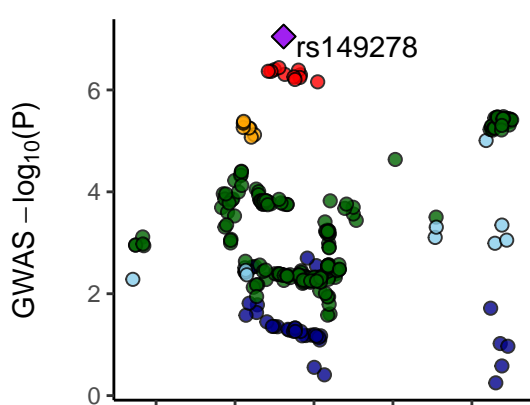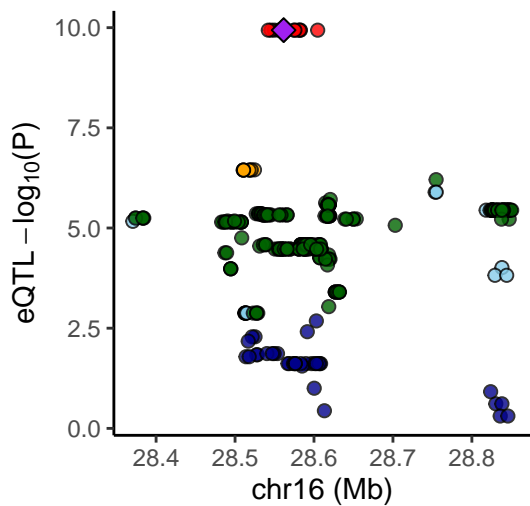

Supplement: Supplementary file 1 [file Supplementaryfile1.zip › Supplementary files/Supplementary Material 13/CD4_Naive_uns_0h_500kb_combined_ENSG00000176476.pdf]

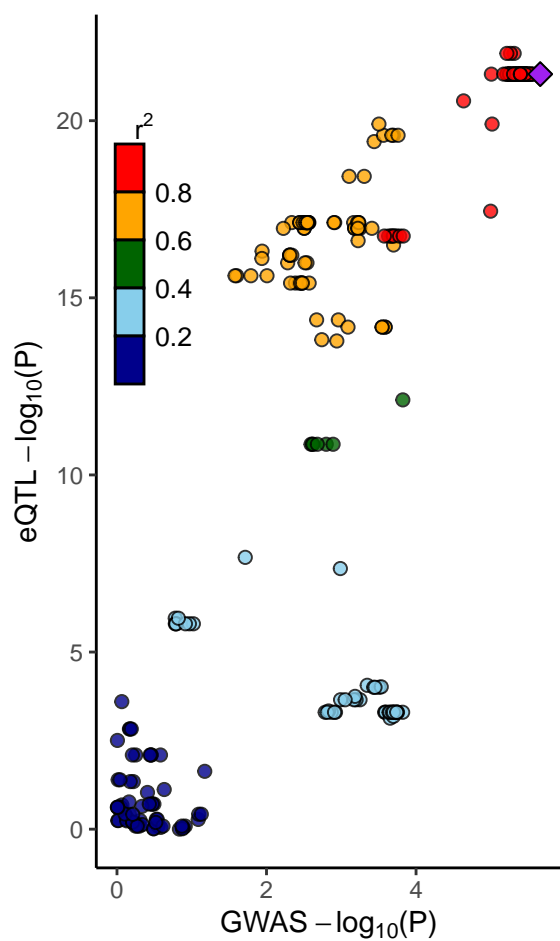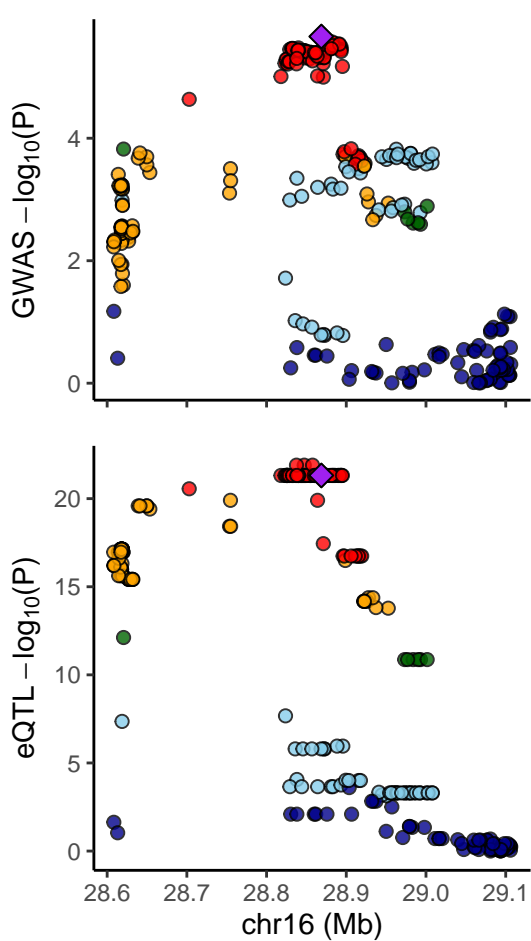

Supplement: Supplementary file 1 [file Supplementaryfile1.zip › Supplementary files/Supplementary Material 13/CD4_Naive_uns_0h_500kb_combined_ENSG00000178952.pdf]

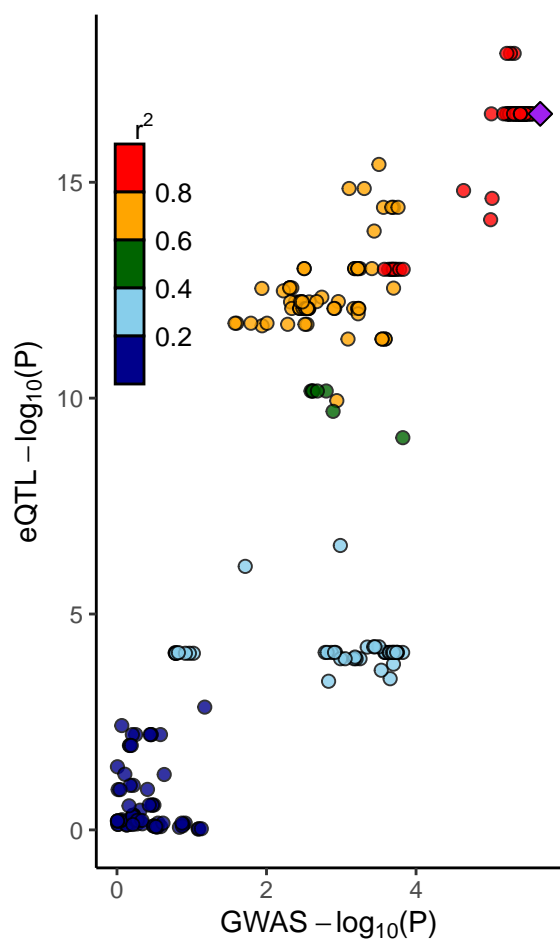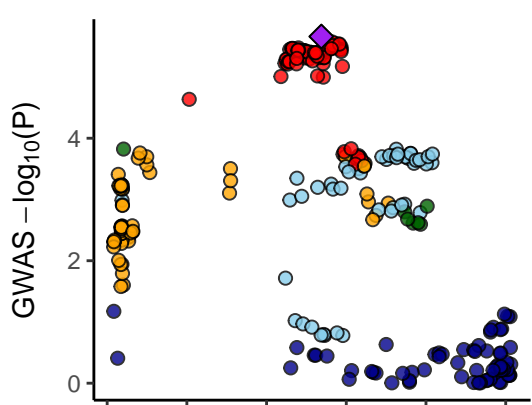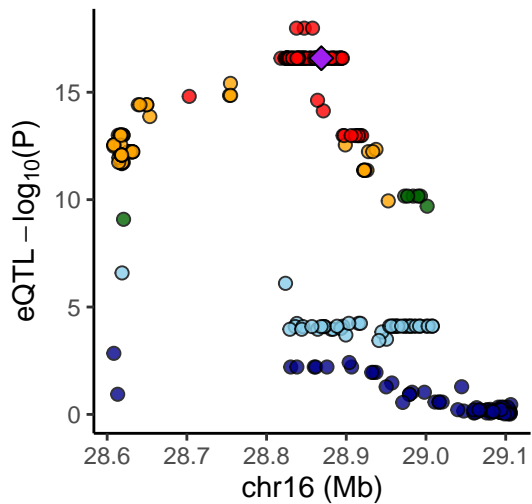

Supplement: Supplementary file 1 [file Supplementaryfile1.zip › Supplementary files/Supplementary Material 13/TCM_0h_500kb_combined_ENSG00000178952.pdf]

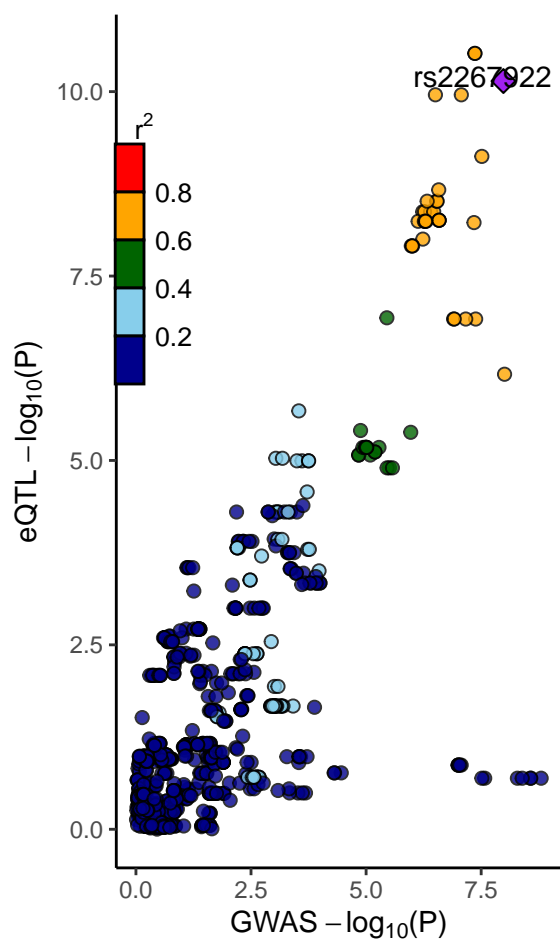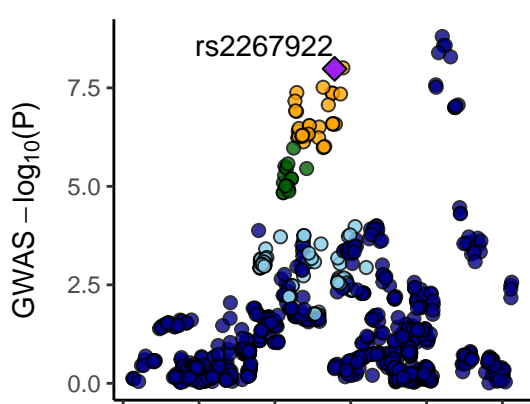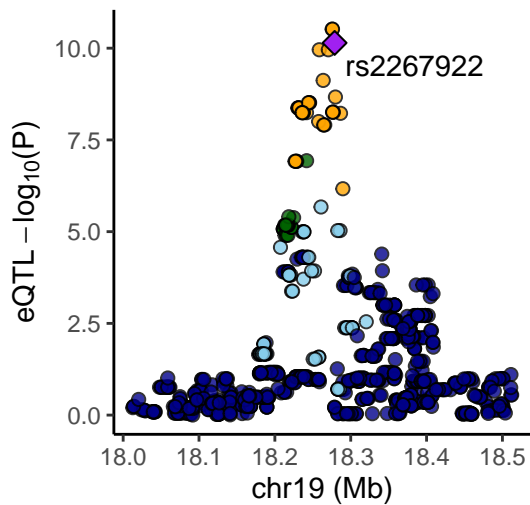

Supplement: Supplementary file 1 [file Supplementaryfile1.zip › Supplementary files/Supplementary Material 13/TCM_5d_500kb_combined_ENSG00000099308.pdf]

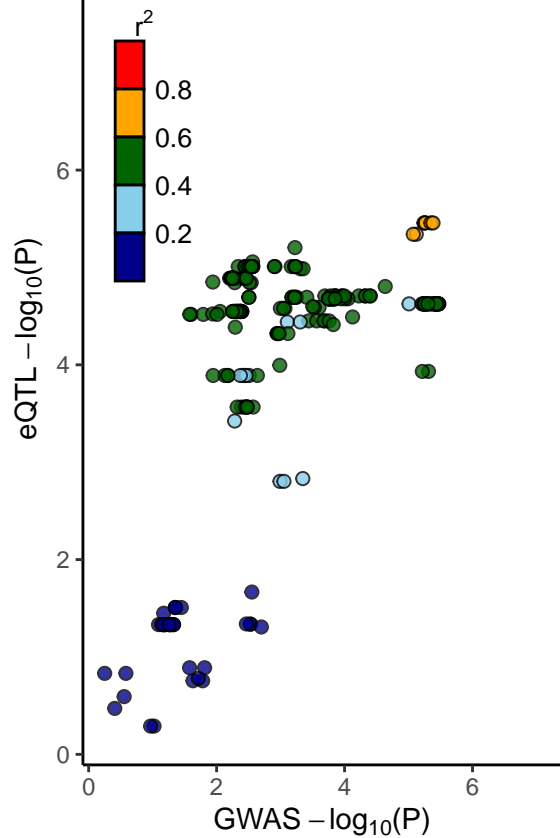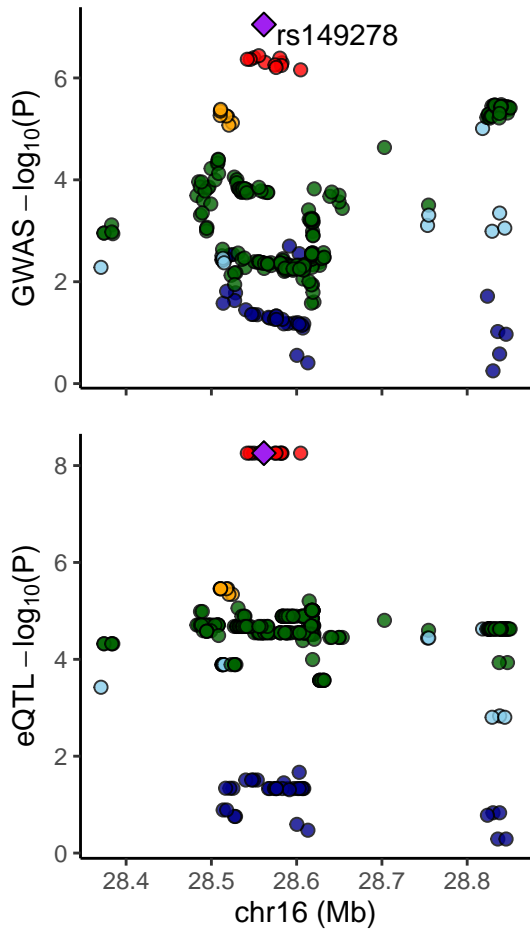

Supplement: Supplementary file 1 [file Supplementaryfile1.zip › Supplementary files/Supplementary Material 13/TCM_5d_500kb_combined_ENSG00000176476.pdf]

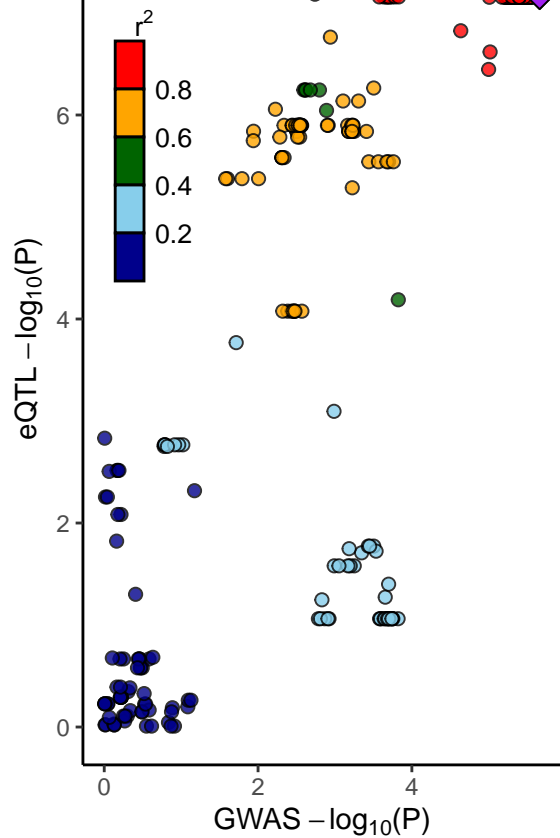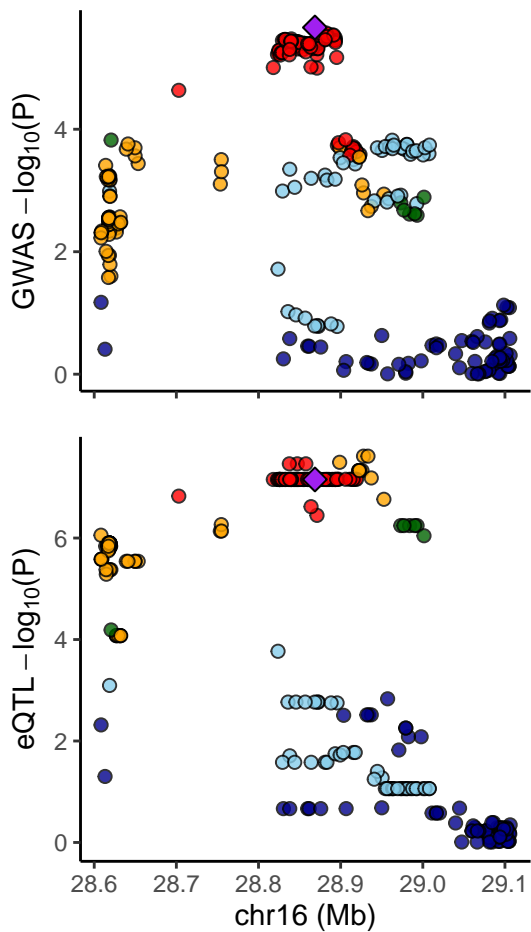

Supplement: Supplementary file 1 [file Supplementaryfile1.zip › Supplementary files/Supplementary Material 13/TEM_5d_500kb_combined_ENSG00000178952.pdf]

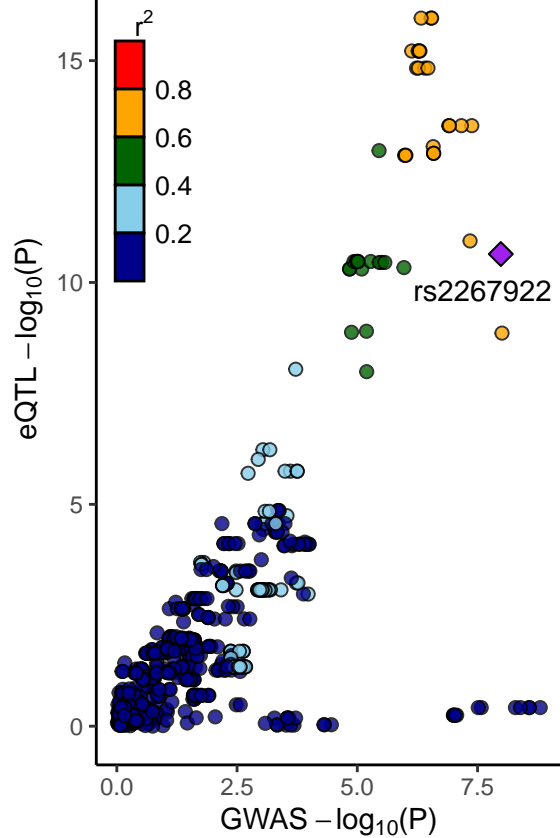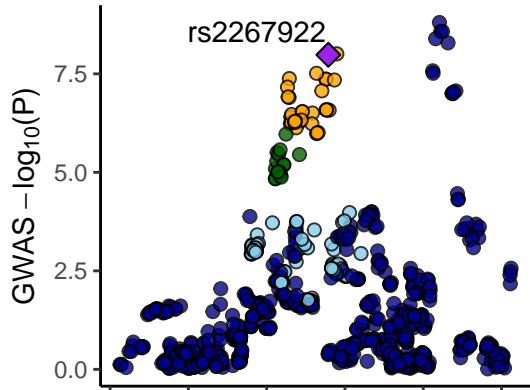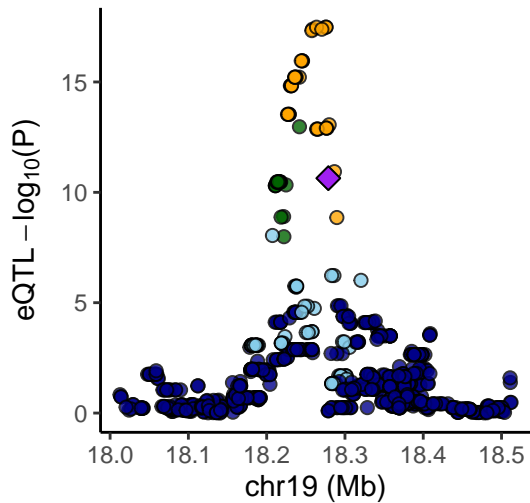

Supplement: Supplementary file 1 [file Supplementaryfile1.zip › Supplementary files/Supplementary Material 13/TN_0h_500kb_combined_ENSG00000099308.pdf]

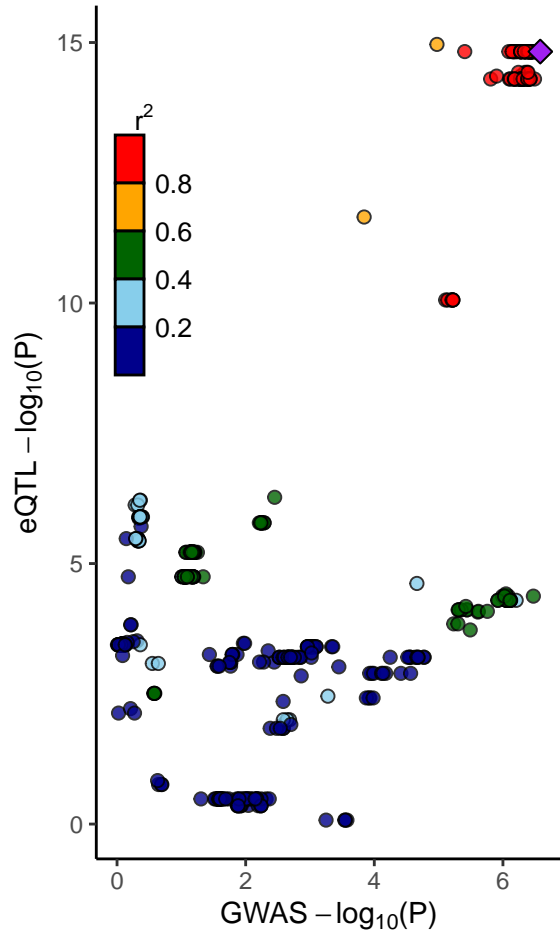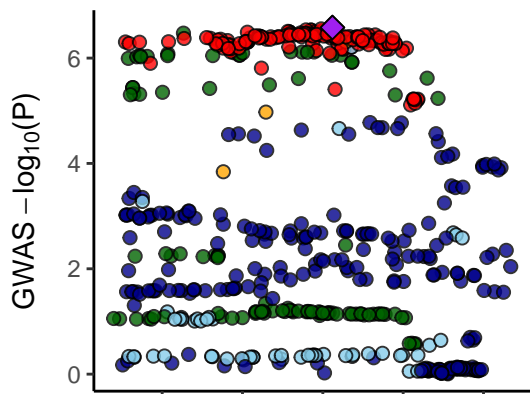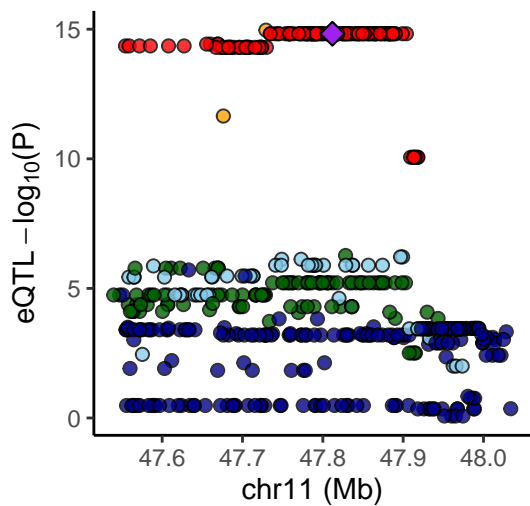

Supplement: Supplementary file 1 [file Supplementaryfile1.zip › Supplementary files/Supplementary Material 13/TN_0h_500kb_combined_ENSG00000109920.pdf]

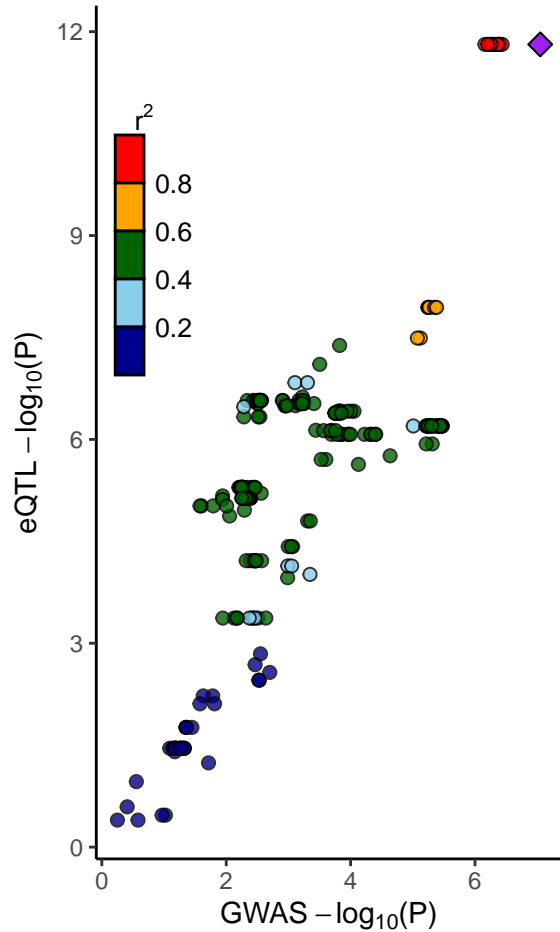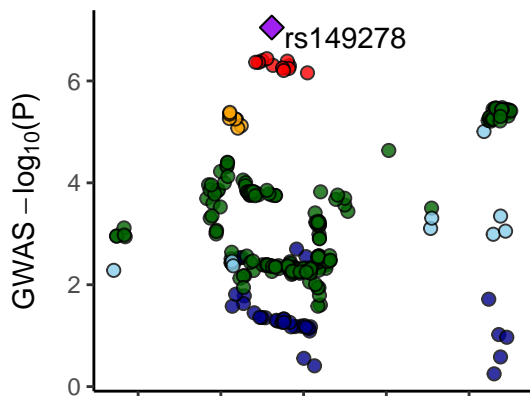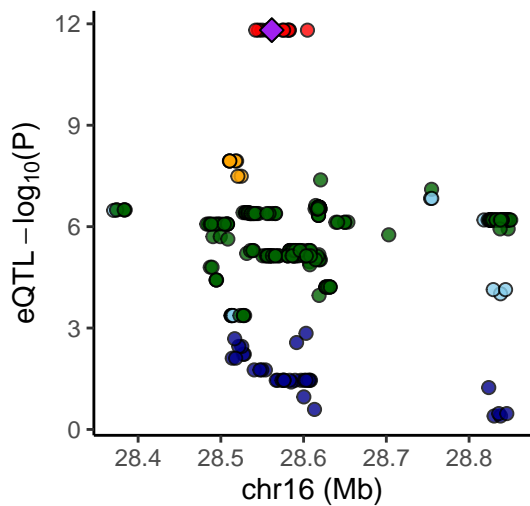

Supplement: Supplementary file 1 [file Supplementaryfile1.zip › Supplementary files/Supplementary Material 13/TN_0h_500kb_combined_ENSG00000176476.pdf]

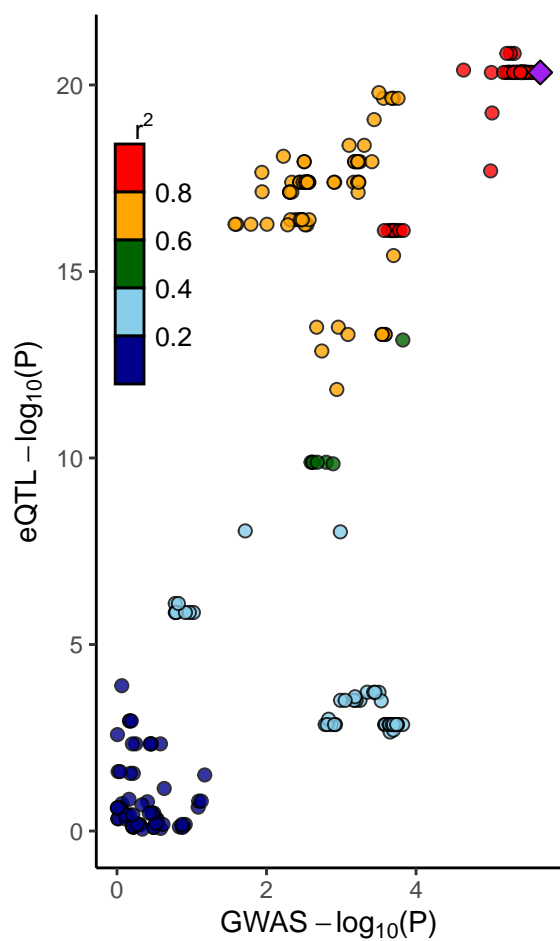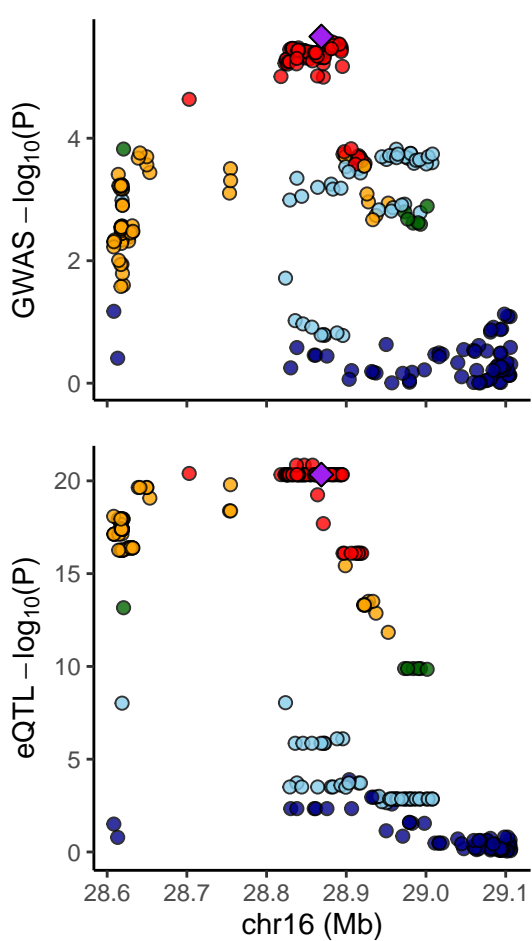

Supplement: Supplementary file 1 [file Supplementaryfile1.zip › Supplementary files/Supplementary Material 13/TN_0h_500kb_combined_ENSG00000178952.pdf]

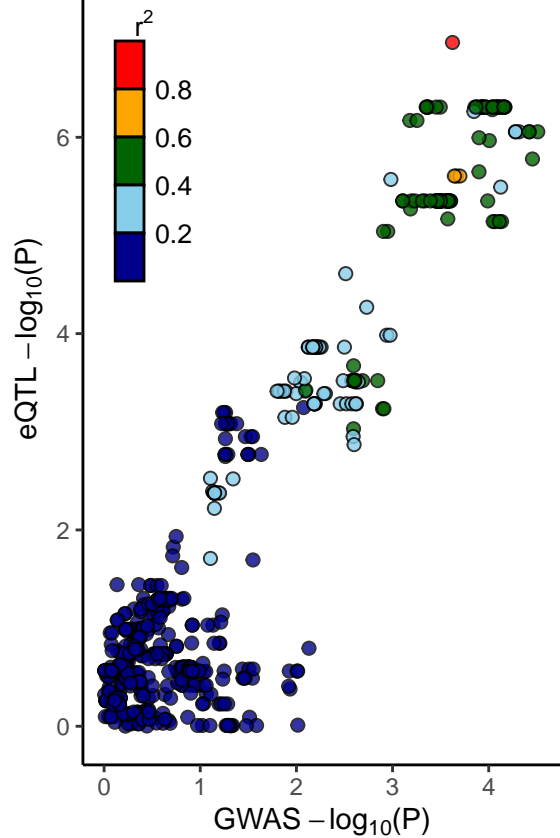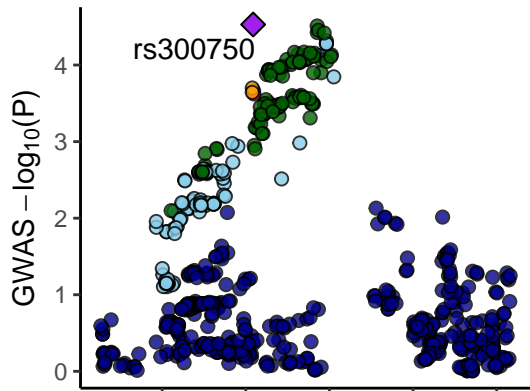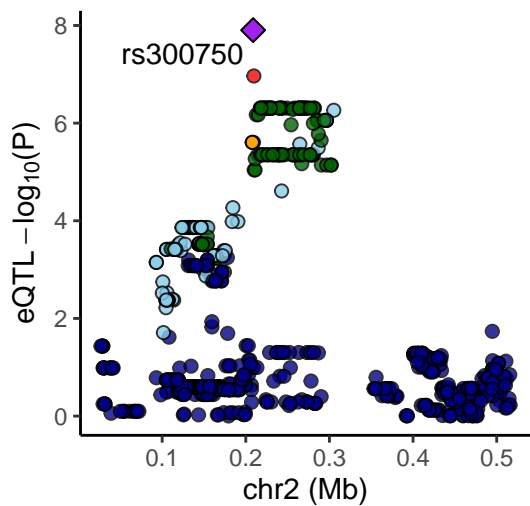

Supplement: Supplementary file 1 [file Supplementaryfile1.zip › Supplementary files/Supplementary Material 13/T_ER-stress_5d_500kb_combined_ENSG00000035115.pdf]
